# A reverse phase protein array based phospho-antibody characterization approach and its applicability for clinical derived tissue specimens

**DOI:** 10.1038/s41598-022-26715-9

**Published:** 2022-12-26

**Authors:** Nan Wang, Li Zhang, Qi Ying, Zhentao Song, Aiping Lu, Achim Treumann, Zhaojian Liu, Tao Sun, Zhiyong Ding

**Affiliations:** 1 Mills Institute for Personalized Cancer Care, Fynn Biotechnologies, Floor 22, Overseas Chinese Innovation Zone, Gangxing 3rd Rd, High-Tech and Innovation Zone, Jinan, 250100 China; 2 Department of Pathology, Beijing Cancer Hospital, No 52. Fucheng Rd, Haidian District, Beijing, 100142 China; 3 Newcastle University Protein and Proteome Analysis, Newcastle University, Devonshire Building, Newcastle upon Tyne, NE1 7RU UK; 4 KBI Biopharma BV, Leuven, Flanders Belgium; 5 Department of Cell Biology, School of Basic Medical Sciences, Cheeloo College of Medicine, Shandong University, Jinan, 250012 China; 6 Department of Haematology, Qilu Hospital, Cheeloo College of Medicine, Shandong University, Jinan, 250012 China

**Keywords:** Biotechnology, Cancer, Molecular medicine, Oncology

## Abstract

Systematic quantification of phosphoprotein within cell signaling networks in solid tissues remains challenging and precise quantification in large scale samples has great potential for biomarker identification and validation. We developed a reverse phase protein array (RPPA) based phosphor-antibody characterization approach by taking advantage of the lysis buffer compatible with alkaline phosphatase (AP) treatment that differs from the conventional RPPA antibody validation procedure and applied it onto fresh frozen (FF) and formalin-fixed and paraffin-embedded tissue (FFPE) to test its applicability. By screening 106 phospho-antibodies using RPPA, we demonstrated that AP treatment could serve as an independent factor to be adopted for rapid phospho-antibody selection. We also showed desirable reproducibility and specificity in clincical specimens indicating its potential for tissue-based phospho-protein profiling. Of further clinical significance, using the same approach, based on melanoma and lung cancer FFPE samples, we showed great interexperimental reproducibility and significant correlation with pathological markers in both tissues generating meaningful data that match clinical features. Our findings set a benchmark of an efficient workflow for phospho-antibody characterization that is compatible with high-plex clinical proteomics in precison oncology.

## Introduction

Reverse Phase Protein Array (RPPA) serves as a powerful tool particularly for quantitative proteomic from finite amount of materials such as patient tissues and is especially useful for post-translational modifications (PTMs) profiling^1^. It is used to elucidate underlying oncogenic mechanisms and allows parallel multi-omics profiling incorporating other omics data on the same set of samples.

However, notwithstanding the effort made in fine-tuning the tissue based RPPA application, several technical bottlenecks still exist. Validation of RPPA-applicable antibodies is a prerequisite for accurate quantification. This relates to the composition of the lysis buffer having significant impact on the tissue solubility, signal intensity, reproducibility and dynamic range as well as downstream processes such as blocking and detection methods that may induce unpredictable impact on the quantification^2–4^. It is generally accepted to perform RPPA antibody pre-screening using western blots (WB) where the standard criteria are predominant or explainable bands at correct molecular weights which should also correlate well with RPPA performed simoutaniously^5–9^. For phospho-antibody validation, more stringent approaches have to be adopted such as perturbation with various stimuli or specific inhibitors^3,4^. In addition, for tissue based profiling, separate antibody validation is recommended as certain discrepancy may exist between tissues and cell lines due to heterogeneity causing decreased specificity as well as preanalytical variables to affect phosphoprotein^9,10^. These altogether complicate the validation process and a better phospho-antibody characterization strategy is in paramount need. Alkaline phosphatase (AP) serves as negative controls in various experimental settings, however its application in high throughput phospho-antibody screening using RPPA has merely been suggested and theoratically this could serve as an ideal control to evaluate antibody performance directly^7,11^. Nevertheless, apart from a study where tyrosine phosphatase was applied as a potential negative control for phospho-peptide characterization^12^, there has been no systematic evidence yet to prove its applicability either in cell line or tissue based profiling.

Formalin-fixed paraffin-embedding (FFPE) is a universal tissue preparation method for pathological processing and this also lies in the scope of RPPA application wherein researchers have made tremendous dedication on improving the clinical applicability of the technology^13–18^. Many have established methods for efficient protein extraction from FFPE samples for downstream RPPA profiling using SDS-based denaturation to allow solubilization of hydrophobic proteins and a heating step for crosslinking reversal to obtain optimal condition for full-length protein extraction^13,19^. Side-by-side comparison between RPPA and immunohistochemistry (IHC) were underway. Many researchers evaluated the quantitative capability of RPPA using several pathological markers and found that human epidermal growth factor receptor 2 (HER2) exhibited nearly 100% concordance rate based on a SuperCurve signal intensity cutoff of 1600 in a cohort of 35 breast cancer samples but not for estrogen receptor (ERα) and progesterone receptor (PR) assayed simultaneously^19^. Similar studies found good correlations of elevatedHER2 expression between IHC and RPPA^18^. Another study assessed 19 breast cancer specimens with HER2 IHC score 0, 1+ , 2+ , 3+ and built a logistic model to predict on an independent testing set, which showed its generalizatable performance^19^. By exploring non-small cell lung cancer (NSCLC) patient, the expression of NapsinA and cytokeratin5 exhibited concordant pattern between IHC and RPPA and upon profiling 150 protein expression using RPPA, researchers discovered elevated expression of PAK2 in squamous carcinoma compared to adanocacinoma indicating its potential role during tumorigenesis^20^. Interestingly, another study demonstrated around 40% concordance rate for 300 antibodies profiled on xenograft prepared FFPE samples compared to their matching FF^21^ and by profiling panels of antibodies on three sample sets (cell lines, breast cancer tissues, renal cancer tissues) consisting of FFPE and counterpart FF, researchers observed varied correlation between these two sample types^22^. These point at the context for successful antibodies application in RPPA and rigorous validation across sample types is of great importance to meet the clinical needs.

In this study, we intended to develope a RPPA phospho-antibody validation workflow using an in-house prepared lysis buffer keeping enzymatic activity of AP and thereby facilitates global phospho-group removal from protein residues and these were served as negative controls for phospho-antibody validation for RPPA directly on-chip. We term this fast-screening procedure as a bottom-up method starting off from RPPA and then cross-validating using WB. We also compared this buffer with other available extraction methods demonstrating its feasibility and advantages on FFPE and FF samples strengthening potential in translational research.

## Results

### Phospho-antibody profiling using alkaline phosphatase treatment in cell lines

**Figure 1:**
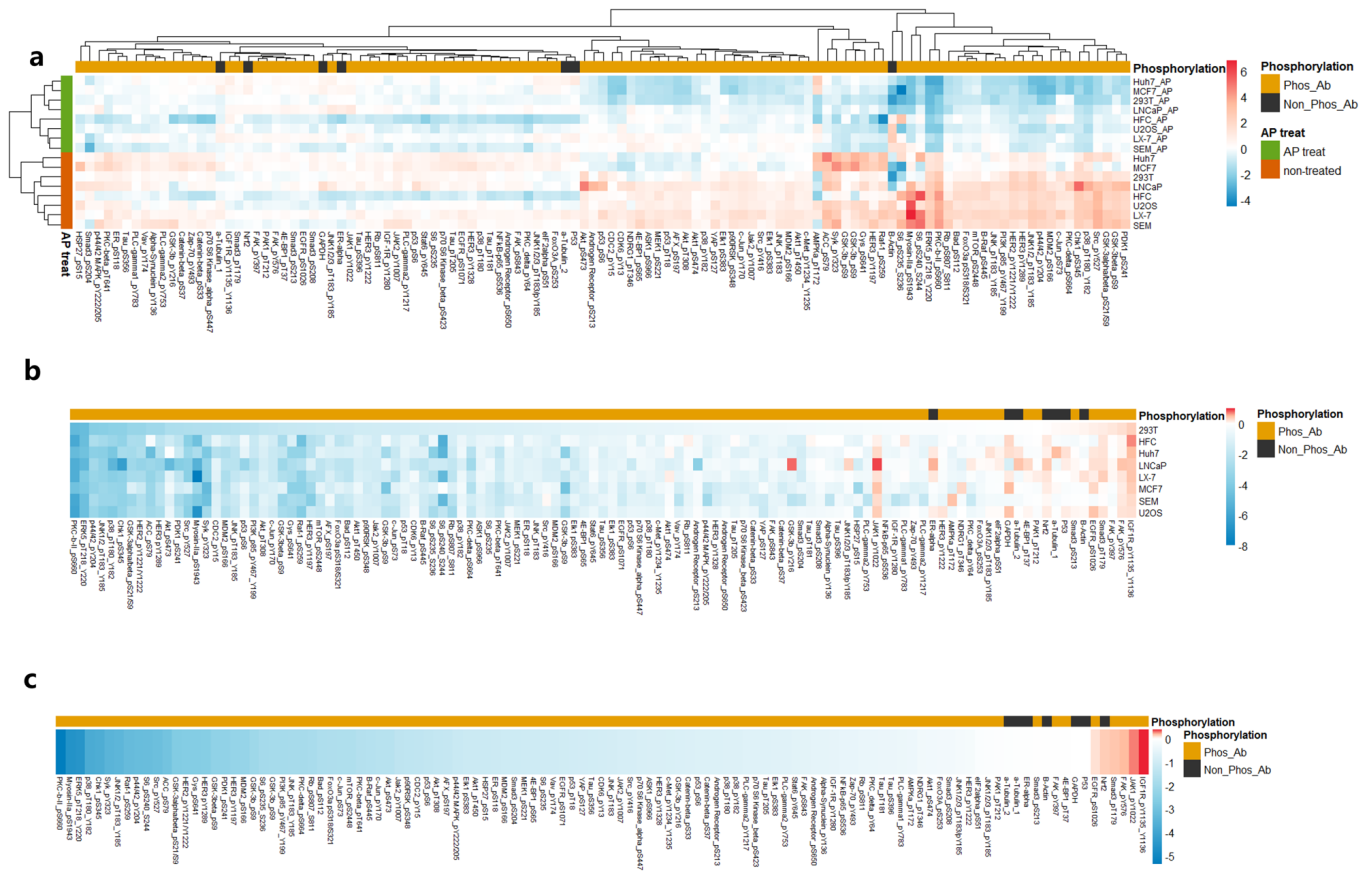
Assessment of alkaline phosphatase treatment induced effect across 8 cell lines versus non-treated controls. (**a**) Unsupervised hierarchical clustering of lysates generated with AGLyse with or without additional AP treatment across 8 cell lines (SEM, LX-7, U2OS, HFC, LNCaP, 293T, MCF7 and Huh7). Phospho-antibodies are marked in yellow and total antibodies (non-phosphor) are marked in black. (**b**) LogFC reduction level of individual antibodies across cell lines. Individual target LogFC is ranked by 293T cells from the lowest to highest (left to right) and rest of cell lines are matched according to LogFC positions of individual targets. c. Averaged logFC across 8 cell lines and data are ranked from lowest to highest (left to right).

Alkaline phosphatase (AP), an enzyme that indiscriminately removes phosphate from phosphorylated Ser, Thr or Tyr residues in proteins. We have previously developed a lysis buffer (AGLyse) that is highly efficient at lysing cells while maintaining APactivity. A panel of 113 antibodies (106 anti-phospho-antibodies and seven protein-specific, but not phosphorylation-specific antibodies) was selected from our antibody library and probed against 8 cell lysates treated or non-treated with AP prior to RPPA printing . As expected, the removal of phosphates from the proteins in cellular lysates resulted in a decrease of the binding of anti-phospho antibodies which was reflected by a clear separation of the observed signals between two groups (Fig. 1a). Ranked logFC of 113 antibodies also demonstrated an enrichment pattern of total antibody in one direction as expected (Fig. 1b,c). Phospho-antibodies that did not show the expected response to AP-induced deduction include those such as pIGF1R (Tyr^1135^/Tyr^1136^), pFAK (Tyr^576^), pJAK (Tyr^1022^), and pSmad3 (Thr^179^) probably due to either antibodies not working for RPPA/WB application or cross-reactivity (Supplementary data S-3).

We then asked whether the AP treatment could serve as an independent predictive factor to assess the phosphorylation antibodies without considering other spot quality measures. Previously, we determined the suitability of antibodies for RPPA purposes using an antibody score that was calculated by taking into account six different factors: (1) spot quality score (percentage of the total sum of RFI excluding “poor” spots defined by ZeptoVIEW software); (2) signal-to-noise ratio (the average fold difference between the RNFI of individual spots and the background obtained from individual spots); (3) dilution linearity score (averaged linearity generated by 8-point dilution across all samples); (4) fold reduction score (average fold reduction in response to AP across all samples); (5) positive reference score (binary score to visually determination of the positive reference quality); 6. Spot graininess/donut effect (binary score to visually determination of homogenous staining and other signal related effects.). Factors 1 to 4 are equally weighted and categorized into three classes respectively (scored 1, 2, 3) with higher having the better performance. Factors 5 and 6 are also Boolean value that determine whether a particular antibody can be used or not. The sum of factors 1–4 multiplied by the two binary values from factors 5 and 6 gave rise to a ranked antibody score ranging from 0 to 12 (Supplementary Data S-4).

Categorizing all antibodies into “Good” or “Bad” according to their scores at a cut-off value of 8 resulted in 67 antibodies being “Good” and 46 antibodies being “Bad”. We then asked whether the AP-treatment induced logFC value could serve as an independent predictor of antibody quality measure. The receiver operating characteristic curve (ROC curve) calculated for an antibody-score cut-off value of 8 and a logFC cut-off value of −0.792 resulted in an area under the curve of 0.825, indicating a reasonable ability for predicting the suitability of phosphorylation-specific antibodies based on the AP-treatment dependent logFC value alone (Chi-square test p < 0.001). To verify independently from the RPPA the veracity of results based on these antibodies, we selected 42 antibodies with logFC ≤ −0.792 and performed western blots in a panel of cell lines wherein. 36 out of 42 antibodies (85%) showed meaningful single bands at the expected sizes, confirming the suitability of this method for high-throughput phospho-antibody screening (Supplementary data S-3 and Supplementary S-5).

### Protein extraction optimization in FFPE and FF tissue specimen

**Figure 2:**
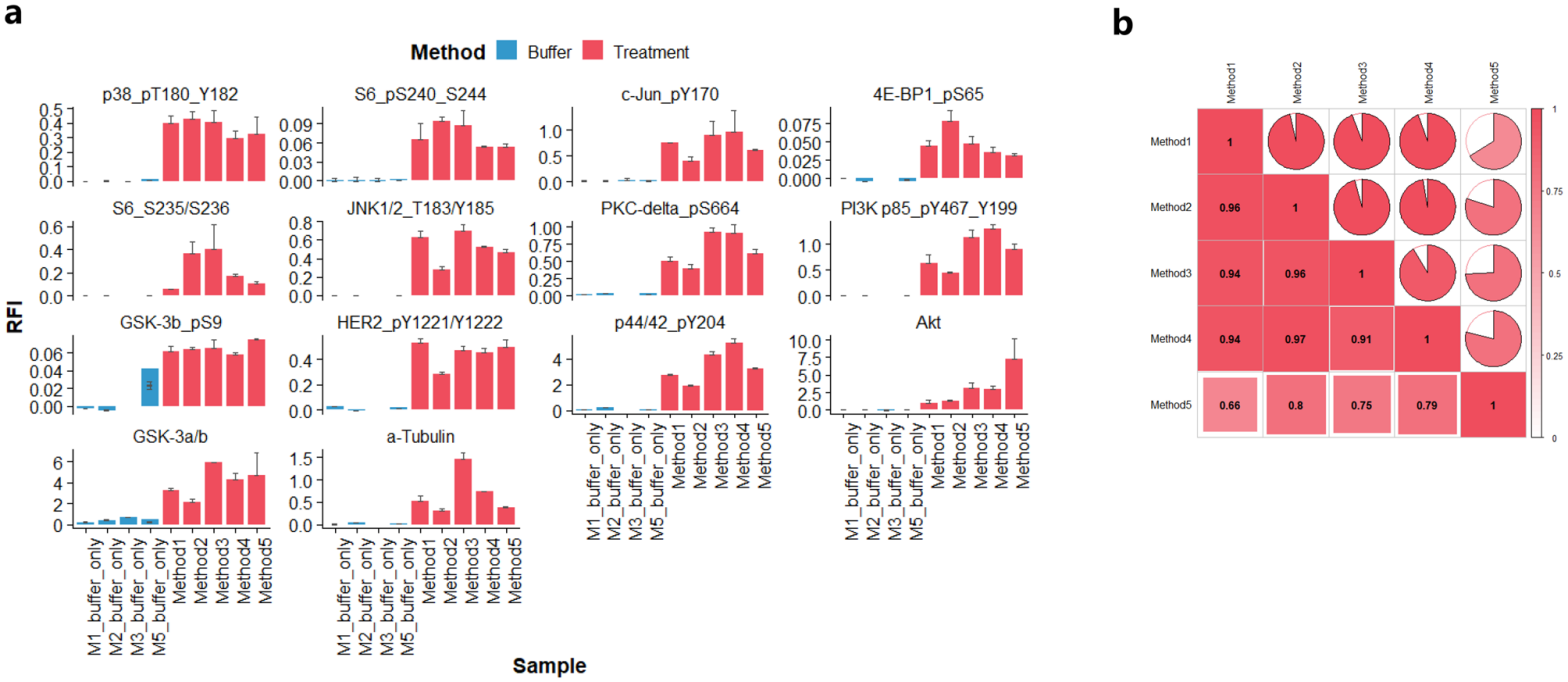
RPPA profiling of multiple targets using 4 extraction methods on FFPE samples. a. Expression profiling of 4 extraction methods (method 1–4) presented by arbitrary units (relative fluorescent intensity RFI) and error bars represent standard deviation from 3 independent extractions. Method 5 is under the same condition as Method 4 without alkaline phosphatase inhibitor. Blue bars are on-chip buffer only controls assessed within the same experiments. b. Inter-method correlation based on 14 protein markers and R^2^ are shown from white to red (0–1).

Upon determining the AP treatment-derived differential expression value as a critical parameter for antibody performance, we then tested the suitability of the buffer based extraction for clinical samples. Using breast-derived FFPE samples, five different lysis methods were compared (Supplementary Table 1a): A panel of 14 pre-validated antibodies (11 anti-phosphorylation antibodies and 3 total protein antibodies) was used to compare between different extraction methods. None of the extraction buffers produced considerable background signals. The correlation between the RPPA signals generated from samples prepared using the five different extraction methods varied but was acceptable for all tested targets (Fig. 2a). The inter-method correlations for methods 1–4 all showed correlation coefficients R^2^ > 0.9 and a p-value of p < 0.01) (Fig. 2b). Interestingly, method 4 showed a higher correlation with the other three methods than with method 5, despite the presence/absence of the AP inhibitors being the only difference between method 4 and method 5.

**Figure 3:**
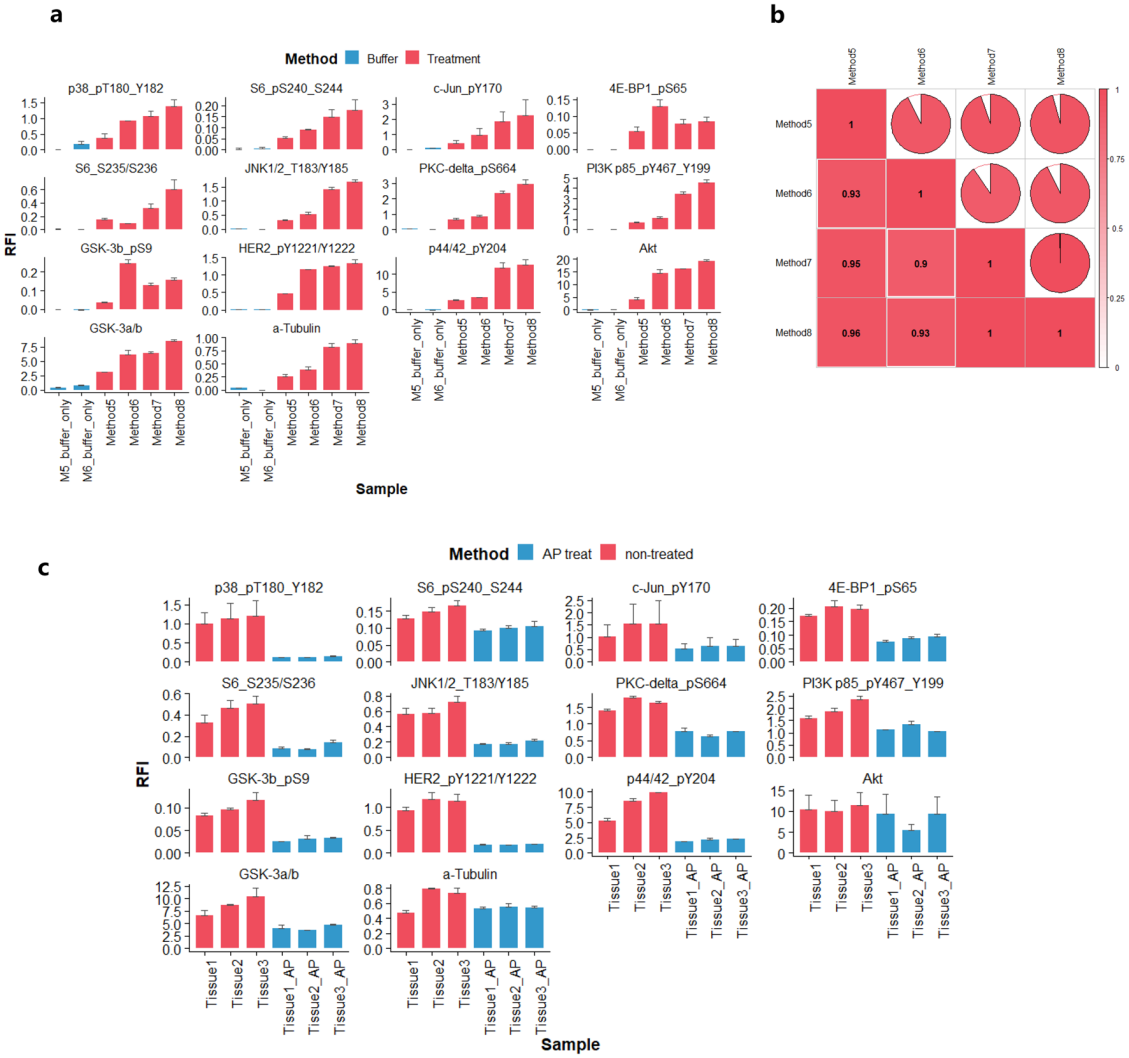
RPPA profiling of multiple targets using 4 extraction methods on FF samples. (**a**) Expression profiling of 3 extraction methods (method 5–8) presented by arbitrary units (relative fluorescent intensity RFI) and error bars represent standard deviation from 3 independent extractions. Blue bars are on-chip buffer only controls assessed within the same experiments. (**b**) Inter-method correlation based on 14 protein markers and R^2^ are shown from white to red (0–1). (**c**) Comparison of AP-treated and non-treated FF samples using RPPA profiling on independent replicates. Non-treated controls are depicted in red and AP-treated are in blue. Each bar represents 3 individual extractions from parallel slides derived from same tissue origins. Error bars are standard deviation between 3 experimental replicates.

For FF samples, well characterized CLB1 lysis buffer was compared with method 5 (Supplementary Table 1b): the same antibodies were used to compare extraction methods 5–8 on the FF tissue samples. Generally, method 5 yielded lower fluorescence signals compared to CLB1 buffer (Fig. 3a). However, the correlation of the readout of the lysates prepared from FF tissue samples was very high for all 14 antibodies (R^2^ ≥ 0.93, p < 0.01, Fig. 3b). Therefore, we continued to apply AP treatment with the established method 5 (AGLyse without phosphatase inhibitor) in three parallel breast tissue selections and each with 3 independent replicates. Using the AGLyse buffer (without the phosphatase inhibitor), a signal reduction was observed for all antibodies used. Of those, pP38 (Thr^180^/Tyr^182^), pHER2 (Tyr^1221^/Tyr^122^) and pGSK3β (Ser^9^) and pERK1/2 (Tyr^204^) showed pronounced reductions of signals whereas negative controls (total antibodies against Akt, GSK-3β and α-tubulin) had much less reduction of the signal intensity in response to AP treatment (Fig. 3c).

**Figure 4:**
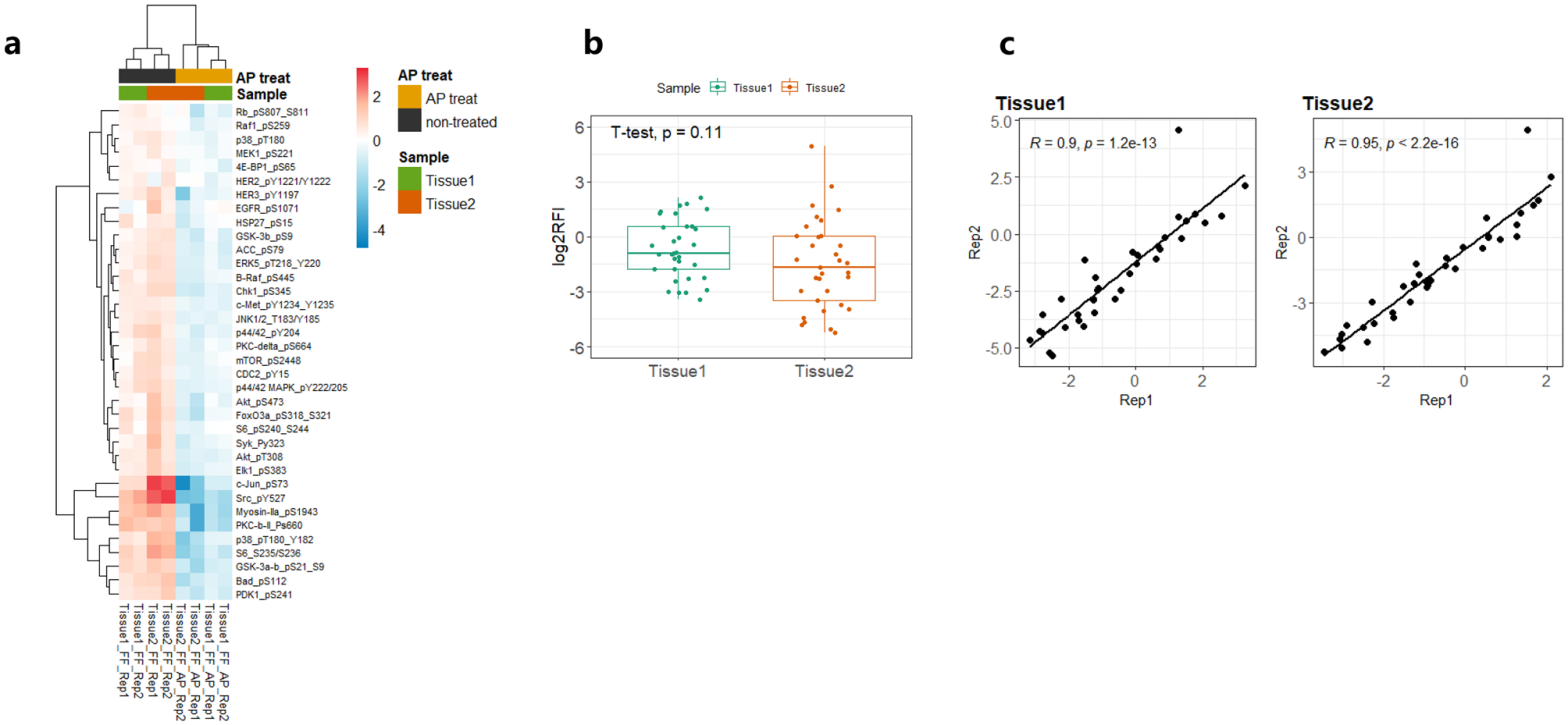
RPPA assessment of AP treatment in FF tissue samples. (**a**) Unsupervised hierarchical clustering of tissue samples treated with (yellow) or without AP (black) based on RPPA profiling of 36 phospho-markers. Rep1 and Rep2 represent two independent tissue sites derived from the same patient and for each site, extraction was performed on two parallel sections separately. (**b**) Overall expression profiling between two tissue sites (Rep1 and Rep2). Data are shown in log2 transformed RFI (relative fluorescence intensity) and unpaired t-test was used to assess the differential expression between two tissue sites. c. Inter-replicate expression correlation presented by RFI from individual tissue sites. Pearson correlation coefficients are shown.

We then expanded analysis with additional FF samples from two independent breast tissue origins each with two parallel extraction replicates using 36 phosphor-antibodies validated in the previous cell line screening. A general reduction of those phospho-signals in response to AP treatment was observed (Fig. 4a) (paired t-test p < 0.05) and the overall phosphorylation abundance between two tissues was very similar (Fig. 4b). (non-paired t-test p = 0.11) and high expression concordance was maintained significantly between experimental replicates (Fig. 4c) (R^2^ > 0.9 and p < 0.001).

These data together provided initial evidence for the suitability of the AGLyse-based protein extraction method for FFPE and FF tissue specimen prior to RPPA profiling. It also demonstrates the feasibility of an generalizable AP treatment workflow for FF tissues that is of great potential for phosphor protein characterization in tissue samples.

### Evaluation of RPPA performance in melanoma FFPE samples

**Figure 5:**
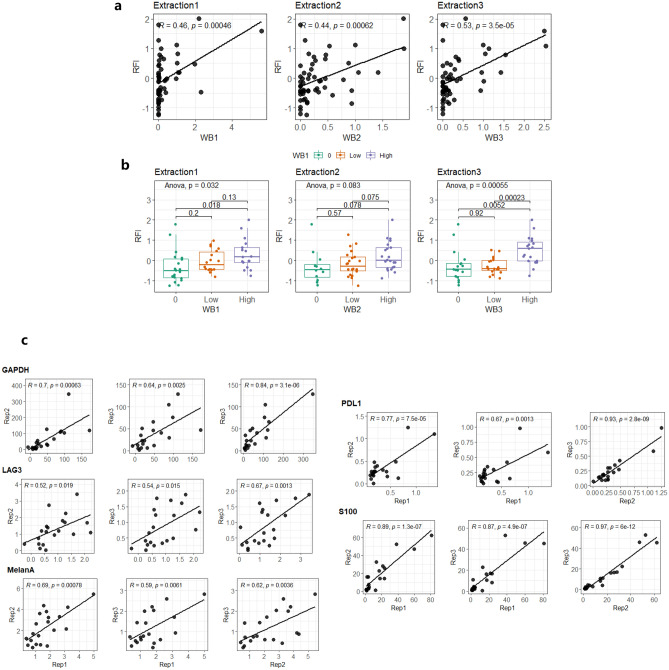
Expression correlation between RPPA and western blot in melanoma 63 FFPE patient samples. (**a**) Direct plotting of expression levels between western blot (arbitrary unit) and RPPA (RFI) from three independent experimental replicates. R^2^ and Pearson correlation are shown accordingly. (**b**) Boxplots representing RFI from three equally divided expression categories based on western blot data. Statistical test results are shown (ANOVA). (**c**) RPPA expression correlation between individual experimental replicates. Five protein markers (GAPDH, PDL1, LAG3, S100, MelanA) in 63 melanoma FFPE patients were tested and R^2^ and Pearson correlation coefficients are shown respectively.

As most clinically available samples are FFPE derived, we applied our approach using a set of melanoma FFPE specimen. Three serially sectioned samples from a total of 63 patients were lysed. Extracted protein lysates were either analyzed using RPPA or using western blotting with a pre-validated Melan-A antibody, a specific melanocyte marker for routine histopathological diagnosis. Although extraction replicate No. 2 had relatively higher yields in comparison to other two replicates, the overall expression correlations between RPPA and western blotting were all retained at similar levels (R^2^ = 0.44–0.53, p < 0.05) (Fig. 5a and Supplementary Fig. S2).

Due to the semi-quantitative nature of western blotting and its sub-optimal reproducibility for inter-experimental quantification, we also equally divided western blot data into three categories based on their signal intensities (0, low and high) and plotted against RPPA data. Two out of three extraction replicates showed significantly different expression at least between low and high groups (ANOVA p < 0.05) indicating largely the consistency between these two methods (Fig. 5b). We then interrogated further with 4 additional antibodies validated for RPPA (GAPDH, LAG3, PD-L1 and S100) and performed inter-experimental comparison for all targets tested where strong correlations were observed (R^2^ between 0.54 to 0.97, p < 0.05) (Fig. 5c). Therefore, by assessing a cohort of samples, we further warranted the robustness of the established methodology for FFPE samples application that is also compatible for downstream RPPA analysis.

### Probing lung cancer FFPE tissues with RPPA

**Figure 6:**
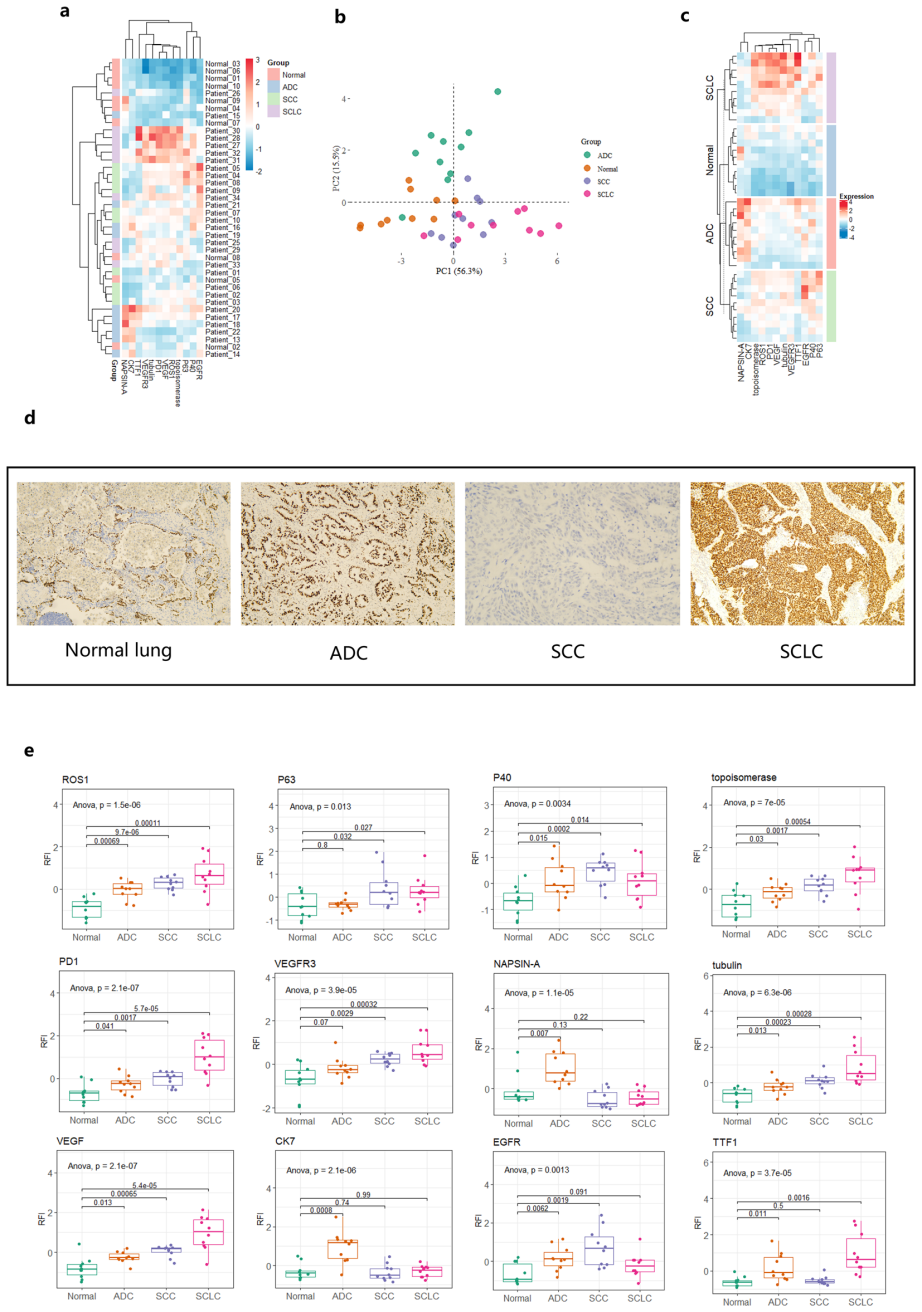
Clustering analysis based on 12-protein based RPPA profiling of lung cancer patient subtypes. (**a**) Unsupervised hierarchical clustering of three lung cancer subtypes (adenocarcinoma ADC, squamous cell carcinoma SCC, small cell lung cancer SCLC) and para-tumor FFPE samples. (**b**) PCA plot of three lung cancer subtypes and normal lung tissues and only PC1 and PC2 are presented. (**c**) Clusteing heatmaps grouped by individual subtypes. (**d**) Immunohistochemistry staining of TTF1 across three lung cancer subtypes and adjacent normal tissues. Images were taken at ×100 resolution. (**e**) Boxplots of individual protein expression shown by RFI in three lung cancer subtypes. Adenocarcinoma ADC, squamous cell carcinoma SCC, small cell lung cancer SCLC and para-tumor FFPE samples are compared and statistical test results are shown (ANOVA).

Upon gathering those data, we designed a test using three subtypes of lung cancers versus normal lung tissue samples. A panel of 13 histopathological protein biomarkers was used and some of which are routinely used for lung cancer subtyping. 20 Non-Small Cell Lung Cancer (NSCLC) patients, of which ten were adenocarcinoma (ADC) and 10 were squamous cell carcinoma (SCC) and ten small cell lung cancer (SCLC) patients were analyzed. 10 adjacent non-cancerous lung epithelium samples were included. A total of 120 FFPE slides were acquired comprising three serial sections per patient. Protein quantification showed similar extraction efficiency between parallel sectioned samples despite one set (rep2) having significantly lower protein yields than others (p < 0.05) (Supplementary Fig. S3). For RPPA, adenocarcinoma markers Napsin A, cytokeratin7, TTF1, squamous cell carcinoma markers p40, p63 and small cell lung cancer markers TTF1 were used. Additionally, EGFR, VEGFR3, VEGF, PD-1, topoisomerase, tubulin-βIII and ROS1 were also used to obtain in-depth protein expression profiles on the same sample sets. Unsupervised clustering revealed distinct expression patterns of proteins in three lung cancer subtypes separating them from non-cancerous epithelium. This was supported by displaying the score plot in PCA analysis indicative of a clear separation of non cancerous tissue extracts from cancer tissue as well as a partial separation of the three lung cancer subtypes from each other (Fig. 6a,b). This can be more clearly appreciated when the tissue sample sets are grouped according to their clinical subtypes (Fig. 6c). ADC tissues showed high expression levels of Ck7 and Napsin A and reduced protein expression of p63 and p40. SCC tissues in contrast showed the opposite pattern with minimal protein expression levels of CK7 and Napsin A and comparatively higher levels of p40 and p63. Both subtypes, ADC and SCC had EGFR expression levels that were significantly higher than those observed in SCLC extracts and control tissues. SCLS extracts displayed higher expression levels of TTF1 than others, which was also confirmed in IHC (Fig. 6d). Of interest, all other markers developed a similar pattern with baseline expression in normal lung tissues, slight up-regulation in ADC and further elevated level in SCC and highest expression in SCLC suggesting a potential expression signature associated with the aggressiveness of the disease (Fig. 6e). For EGFR and p40, as two antibody strains were available for RPPA respectively, both were tested against each other and showed optimal consistency (R^2^ = 0.94, p < 0.001) again reconfirming the technical robustness and compatibility for FFPE derived tissue specimen (Supplementary Fig. S4).

## Discussion

The application of novel multi-plex proteomic technologies for clinical tissue sample profiling has been significantly impeded by stringent sample pre-processing, preservation and other technical factors. Although numerous efforts have been made to develop clinical-compatible methodologies, such analytical approaches are yet not widely available. RPPA has great advantages for quantitative assessment of clinical tissue specimens due to its simple preparation workflow, minimal sample consumption, high sensitivity and parallel high-plexing capacity^23^. Therefore, many others have made early breakthroughs in optimizing pre-analytical variables in order to obtain reliable data for clinical interpretation especially on FFPE samples^13,15,16,18,19,21,24,25^. Herein we developed an efficient workflow by employing an in-house buffer (AGLyse) and comprehensively tested its reliability on both sectioned FF and FFPE tissue materials.

AGLyse contains a milder denaturing condition allowing alkaline phosphatase to function and thus it has great potential for phospho-antibody screening which is critical for RPPA application. This method was preliminarily used in previous experiments where the specificity of pP53 (Ser^15^) antibody was tested^26^. By non-discriminative stripping of all phosphor-groups on protein analytes, 106 selected phospho-antibodies were evaluated alongside with 7 antibodies targeting total proteins as controls. For all cell line lysates, AP treatment induced a clear pattern reflected by perfect clustering. Ranked fold-change further describedthe performance of individual phospho-antibodies and counterpart total antibodies. Notably, a portion of phospho-antibodies had even elevated expression upon AP induction probably due to antibody specific reasons either via interaction with alkaline phosphatase non-specifically or intrinsic incompatibility with RPPA workflow and therefore were classified as incompatible antibodies for RPPA application. The on-slide antibody performance is another analytical factor in RPPA antibody evaluation especially for fluorescence-based detection approaches and therefore multiple parameters are routinely used mainly by checking signal-to-background ration, spot quality and reproducibility, dilution linearity, positive reference spot quality and signal saturation^8,27,28^. These are sometimes tedious and require manual interpretation causing unavoidable errors. We sought to evaluate whether the cell line-based approach can serve as an independent deterministic parameter regardless of others analytical factors. Under a LogFC = −0.792 and antibody score = 8 (AUC = 0.825), Chi-square tests revealed a strong association between multi-parameter evaluation and LogFC alone (p < 0.001) providing initial evidence for antibody quality determination without considering other decisive parameters, however this approach still needs validation with more phospho-antibodies. As we termed this as a bottom-up screening method, western blots were performed consequentlty on antibodies categorized with LogFC ≤ −0.792. Of the 42 phospho-antibodies tested, 36 have successfully showed target-specific bands by whereas 6 had non-specific detection in contrast stressing the need of orthogonal WB validation for downstream RPPA. Nevertheless, our approach still provide an efficient strategy for high throughput phospho-antibody screening using RPPA and selected candidates can also be used for downstream RPPA profiling speeding up the analysis of phospho-proteomics profiling.

In the followup study, two types of tissue (FFPE and FF) were assessed against a range of tissue protein extraction protocols. For FFPE, commercial Qproteome extraction (method 1) and Tris-SDS based extraction (method 2/3) both generated decent signals over the buffer only background across 14 selected antibodies (phospho and total). Though certain degrees of variation on some targets were observed (GSK3β, α-tubulin, Akt and pPI3K-p85(Tyr^467/199^) in particular) with or without sonication in method 2, their correlation was generally acceptable. This could be partially attributed to the inter-slide variation of tissues or alteration of post-translational modification prior to fixation and paraffin embedding. Our AGLyse based methods (Method 4/5) both produced comparative signals with slight reduction of some phosphorylated proteins (Method 5), which was probably caused by removal of phosphatase inhibition. Despite these facts, their overall concordance with other established methods was strikingly high (R^2^ between 0.75–0.96, p < 0.05) suggesting the reliability of the methodology for FFPE derived sample profiling. As for FF samples, previously characterized CLB1 buffer was used in various extraction conditions (Method 6–8) as positive controls^21,22,29^. All of them have generated analyzable signals over the buffer background with heat-induced methods (method 7/8) having highest signal levels throughout on the same set of antibodies. This suggested heat-induction may also be critical in FF tissue protein extraction to untangle protein molecules. AGLyse mediated extraction (method 5) had again generated decent signals above the buffer only background (method 5 buffer only), despite their global lower signals compared to other CLB1 based methods. Apart from that, the inter-method correlation was remarkably high (R^2^ 0.93–0.96) indicating FF samples may be more stable in general for RPPA phospho-proteomic profiling. Of note, under both extraction conditions (FFPE and FF), tissue autofluorescence was negligible reconfirming the feasibility of the data. By incorporating AP treatment in FF sample setting, all phospho-antibodies exhibited expected reduction upon AP treatment whereas total antibodies (GSK3β, α-tubulin and Akt) remained unaffected. Data from the inter-extraction replicates were reproducible suggesting the consistency of the method and notable variation on some targets (pS6(Ser^235/236^) and p44/42(Tyr^204^)) could be explained by heterogeneity between different sample origins. Extended analysis on 36 pre-validated phospho-antibodies also proved the robustness and fidelity of AGLyse with AP treatment for phospho-antibody screening in FF tissues. Antibodies used for RPPA need thorough validation across sample types and for samples from tissue origins, high complexity may result in immune cross-reactivities leading to misinterpretation of data^8,24,30^. As phosphorylation is the most extensively studies post-translational modification in cancer biology and plays crucial roles throughout tumor progression, our method may vastly accelerate phospho-antibody validation efficacy for tissue proteomics in clinical translational research.

Our tentative work from melanoma FFPE samples exhibited comparable and reproducible data for Melan-A. As either WB or IHC are semi-quantitative and digitally transformed quantitative readouts from WB are extremely difficult for inter-experimental comparison, direct assessment of RPPA data with conventional WB and IHC might be implausible and many others have also shown moderate side-by-side comparison results^16,17,19,31^. RPPA in that sense as a bulk sample high-plex analytical approach may serve as an assistive analytical tool due to its incapability of resolving heterogeneities that have significant impact on expression profiles especially in tumor purity low samples. Despite these, RPPA still has great power for multi-target proteomic profiling reflected by consistant data generated from PDL1, LAG3, S100B and GAPDH on a single tissue section.

In a final setup, we tested the capability of the approach in cancer subtyping. On a set of lung cancer samples (ADC, SCC and SCLC and paratumor), we demonstrated .the ability of RPPA to classify patients using conventional pathological markers (Napsin A, cytokeratin7, TTF1, p40/p63 and EGFR).. Although p40 (a SCC differentiation marker) was up-regulated in SCC and ADC in part as compared to normal lung (ANOVA p < 0.05), it failed to discriminate SCC and ADC (p > 0.05) and oppositely, although p63 (a SCC differentiation marker) was able to distinguish SCC and ADC, it failed to separate from normal epithelium. p40 was discovered as a more sensitive marker for SCC subtyping than p63, however in RPPA profiling its performance was inferior to the latter one^32^. Despite intrinsic variability amongst all patients tested, this could be attributed to the defects of RPPA in dealing heterogeneous samples. Of interest, all other protein markers tested (ROS1, PD-1, VEGFR3, VEGF, topoisomerase and tubulinβ-III) constituted an elevated expression signature with normal lung tissues having the lowest expression, NSCLC showing intermediate expression and the neuroendocrine subtype SCLC presenting the highest levels. This was in agreement with the aggressiveness of the clinical phenotypes and prevalent expression pattern of topoisomerase, tubulinβ-III, VEGF, VEGFR3 and PD-1 in SCLC were previously documented at histopathological or cellular levels^33–37^. ROS1 rearrangement was extensively characterized in lung cancer and our finding is largely in line with the findings previously^38^. Given that the C-terminal kinase domain function was likely retained (antibody targeting C-terminal of ROS1) and small cell transformation results in loss of fusion or mutation in kinase domains conferring resistance to tyrosine kinase inhibitors (TKIs), our data indicate yet a functional competent ROS1 throughout tumor progression and strengthens the need to disentangle ill-defined mechanisms of truncated ROS1 isoforms during tumor transformation^39^. Though our ROS1 antibody was not designed specifically for fusion detection in IHC, RPPA has eminent potentials to serve as a fast and efficient pre-screening tool using mutant specific antibodies prior to IHC and fluorescence in-situ hybridization (FISH)^40^. Conclusively, by utilizing an in-house developed buffer system, our systematic work provides a novel RPPA workflow that is universally compatible for fast phospho-antibody screening in cell lines and FF tissues. Albeit phosphoprotein quantification is challenging in FFPE due to pre-analytical factors, our validation using melanoma and lung cancer FFPE samples delivered promising potential of RPPA as a robust translational tool for targted proteomics.

## Methods

### Cell lines and tissue sample acquisition

For RPPA screening, 8 cell lines (Huh7, HFC, MCF7, SEM, 293T, LNCaP, LX-7, U2OS) were obtained from various vendors and identities were confirmed using Short Tandem Repeat assays (STR). All the rest of cells used for western blotting were also STR confirmed (all cell line information is included in Supplementary S-1). Cells were routinely cultured in recommended medium with 5% CO_2_ and 10% fetal calf serum (FCS). EGF, IGF and Insulin treatment for phosphor-antibody validation were obtained from ProSpec (CYT-217, CYT-216) and Signa (12643) respectively. Transiently transfected over-expression clones in 293T cells were conducted by VigeneBio (https://www.vigenebio.cn). Breast tumor tissue specimens used for extraction optimization, buffer comparison and AP treatment were obtained from Department of Pathology in Beijing Cancer Hospital. 189 Melanoma patient FFPE slides derived from 63 patients and 120 lung cancer patient and normal FFPE tissues from total of 40 individuals were also obtained from Beijing Cancer Hospital upon internal review board approval with written consent from patients for research purpose use. Tissue samples were all confirmed with neoplastic cellular content of over 50% as passing criteria.

### Protein extraction and alkaline phosphatase treatment for phospho-antibody characterization

For in vitro cell line work, in-house developed extraction buffer (AGLyse) mainly containing high percentage of SDS and Tris with protease inhibitor and phosphatase inhibitor (Roche Applied Science) were used^26^. Counterpart buffer AGLyse without phosphatase inhibitor was prepared to allow AP to function at an expected level. Cells were harvested following 2 times of cold PBS washes and then followed by 4 °C incubation with agitation for 30 min. Supernatants were kept for downstream application. To generate paired AP treated samples, buffer adjusted 200 μg of total protein lysate (per sample) was incubated with 10 μg alkaline phosphatase (Sigma-Aldrich P0114-10KU) with NaCl (10 mM), MgCl_2_ (0.1 M), Tris (5 mM) and Dithiothreitol DTT (10 mM) at 37 °C for 1 h before RPPA processe. To generate a balanced AP reaction condition, protein concentrations were adjusted to3μg/μl with AGLyse and diluted with ultrapure water to 2.2 μg/μl before reaction keeping all ratio of buffer components within range.

### Lysis condition comparation and optimization for RPPA (FF and FFPE)

For fresh frozen sections (10 μm thickness and ranging between 1cm^2^ size across all samples), we compared our extraction method with CLB1 buffer (7 M urea, 2 M thiourea, 4% CHAPS, 1% Dithiotretol, 4 mM spermidine, 2% pharmalyte, Roche protease inhibitor cocktail) under various conditions used previously that are compatible with Zeptosens array detection as well as other tissue-based extraction methods for RPPA^19,22,41^. Generally, AGLyse buffer and CLB1 buffer were applied directly on tissues at approximately 50-100 μl and tissues were incubated for 30 min at room temperature or on ice and followed by centrifugation (5 min at 15,000*g*). Supernatants were stored at −80 °C until downstream applications. The extraction processes were followed with or without extra sonication steps to facilitate the homogenization of tissues. For FFPE samples, we compared the standard CLB1 buffer with a range of lysing conditions (Supplementary Table 1a). We used the Qproteome FFPE protein extraction buffer accordingly to manufacturer instruction and literatures^17,24^. We also used a Tris-SDS based buffer (20 mM Tris–HCl, pH 9 with 2% SDS) optimized for RPPA previously with or without sonication in different combinations^25^. For fresh frozen samples, CLB1 and AGLyse buffers (-AP) were used with additional intermediate processes and all methods are summarized in Supplementary Table 1b.

### Reverse phase protein arrays

All extracted and processed lysates were quantified using Bradford assay (Sigma-Aldrich) and diluted with array spotting buffer (CSBL1) to a final concentration of 0.2 μg/μl prior to array deposition. Zeptosens hydrophobic chips (ZeptoCHIP) were used (NMI-TT GmbH) and serial diluted samples (100%, 75%, 50% and 25%) were prepared using a liquid handling robot (Tecan) and followed by piezo-electric printing (Nanoplotter NP2.0 GeSiM). Chips were blocked with ZeptoFOG blocking station using Bovine Serum Albumin containing BB1 buffer for 1 h and followed by primary antibody incubation overnight at 4 °C. Alexa-Fluor647-conjugated secondary antibodies (Abcam) were then incubated for 2 h at 4 °C prior to imaging with a ZeptoREADER instrument. All antibodies used for RPPA are listed in Supplementary S-2. All antibody incubations were carried out in CAB1 assay buffer. For on-chip protein loadings, duplicated slides were incubated with 0.45 μm membrane-filtered freshly-prepared SyproRuby staining solutions (Invitrogen) for 30 min. Chips were then washed with ultra-pure water for 3 times prior to imaging on ZeptoREADER. To evaluate the non-specific signals potentially generated under buffer only conditions, buffer negative controls (buffer only) were evaluated using the same procedure for parallel comparison. For assay negative controls, spare arrays were incubated with secondary antibody only to assess non-specific binding and potential tissue derived autofluorescence. All images were analyzed with ZeptoVIEW software using optimized setting.

### Western blotting

Samples were resolved in SDS-PAGE and transferred to PVDF membranes 1 h at 4 °C (Amersham Hybond). Membranes were blocked by 5% BSA and hybridized with different primary antibodies at optimized dilutions indicated (Supplementary S-2). Chemiluminescent signals were captured by horse radish peroxidase (HRP) conjugated secondary antibodies for rabbit or mouse (SA00001-1/SA00001-2, Proteintech Groups) and visualized by enhanced chemiluminescence detection reagents (34578, ThermoFisher). The abundances of signal were digitally quantified by Image Lab software with a gel imaging system (ChemiDoc XRS + Bio-Rad) and presented as arbitrary units of density. All antibody western blotting data are provided in Supplementary S-3 and Supplementary S-5.

### Immunohistochemistry

Immunohistochemistry (IHC) was performed on formalin-fixed paraffin-embedded (FFPE) patient sections with an automated staining system (Dako Link48) using TTF1 primary antibodies (ZSGB-Bio Cat: ZM-0270). After deparaffinization and rehydration in xylene and ethanol, antigen retrieval was performed in 1× EDTA retrieval solution (pH 8.0) (E-5134, Sigma) with heating. Inactivation of endogenous peroxidase was performed by adding enough drops of 3% hydrogen peroxide (H324-500, Fisher) to cover the whole section for 10 min and followed by primary antibody incubation at recommended dilution. After 60 min incubation at 37 °C, slides were washed twice with PBS, incubated with goat-anti-rabbit antibody (E046201, Dako) and then developed with DAB substrate (k3468, Dako) for 5–8 min depending on the antibodies. Slides were counterstained with hematoxylin (CTS-1090, Biotechnologies) and were scanned with brightfield pathology microscope at 20× magnification (Aperio CS2, Leica) and processed by ImageScope software (Leica). Information on all other chemicals and reagents used in the article can be found in Supplementary S-2.

### Data analysis and statistical rationale

To compare the performance of phospho-antibodies in multiple cell lines, data were median-centered across samples and processed with unsupervised hierarchical clustering. For correlation analysis, pearson correlation was used. All inter-group comparisons were statistically analyzed by student’s t-test, Mann–Whitney test or one-way ANOVA depending on the experimental contexts and p-value of 0.05 was considered significant unless elsewhere stated. The evaluation of “good” and “bad” antibodies was performed with an equal-weighted linear combination of spot quality, signal-to-noise ratio, dilution linearity, AP fold reduction multiplied by two binary factors assessed manually: the detailed formula calculation method and scoring description are presented in Supplementary Table 2. We used different antibody scores (between 5 and 8) to define “good” and “bad” and their corresponding logFC to assess the predictive accuracies (AUC). We used an intermediate score considering the balance between AUC and the “good” and “bad” antibodies in real practice and performed Chi-square tests to evaluate the association between AP induced reduction (LogFC) and binary classified antibody categories (“good” or “bad”). All data analysis was carried out using R statistical and graphical interface.

All methods were carried out in accordance with relevant guidelines and regulations.

### Ethics approval and consent to participate

The study included tumor specimens from all patients who underwent surgical resection (in Beijing Cancer Hospital). The study was approved by Beijing Cancer Hospital Ethics Committee for research purpose.

### Statement for methodology

All methods were carried out in accordance with relevant guidelines and regulations.

## Supplementary Information

Supplementary Figures.

Supplementary Information 1.

Supplementary Information 2.

Supplementary Information 3.

Supplementary Information 4.

Supplementary Information 5.

Supplementary Information 6.

Supplementary Tables.

## Data Availability

The datasets used and/or analysed during the current study are available from the corresponding author on reasonable request (nan.wang@fynnbio.com). These include and not limited to original RPPA data.

## Abbreviations

ADC: Adenocarcinoma
AP: Alkaline phosphatase
AUC: Area under curve
BSA: Bovine serum albumin
CAB1: Cell assay buffer1
CK7: Cytokeratin7
CLIA: Clinical laboratory improvement amendments
CLB1: Cell lysis buffer1
CSBL1: Spotting buffer for lysates
EGF: Epidermal growth factor
EGFR: Epidermal growth factor receptor
ER: Estrogen receptor
FC: Fold-change
FCS: Fetal calf serum
FF: Fresh frozen
FFPE: Formalin-fixed and paraffin-embedded
FISH: Fluorescence in-situ hybridization
HER: Epidermal growth factor receptor
IGF: Insulin growth factor
IHC: Immunohistochemistry
NSCLC: Non-small cell lung cancer
PD-1: Programmed cell death protein-1
PR: Progesterone receptor
PTM: Post-translational modification
RFI: Relative fluorescence intensity
RNFI: Relative normalized fluorescence intensity
RPPA: Reverse phase protein arrays
SCC: Squamous cell carcinoma
SCLC: Small cell lung cancer
SDS: Sodium dodecyl sulfate
TKI: Tyrosine kinase inhibitor
TTF1: Thyriod transcription factor-1
VEGF: Vascular endothelial growth factor
VEGFR: Vascular endothelial growth factor receptor
WB: Western blots
