# Supplementary material for: A reverse phase protein array based phospho-antibody characterization approach and its applicability for clinical derived tissue specimens
Source: Sci Rep. 2022 Dec 26;12:22373. doi: 10.1038/s41598-022-26715-9 (PMC9792559; doi:10.1038/s41598-022-26715-9)
Supplement: Supplementary file 1 — Supplementary Figures. [file 41598_2022_26715_MOESM1_ESM.pptx]

## Slide 1
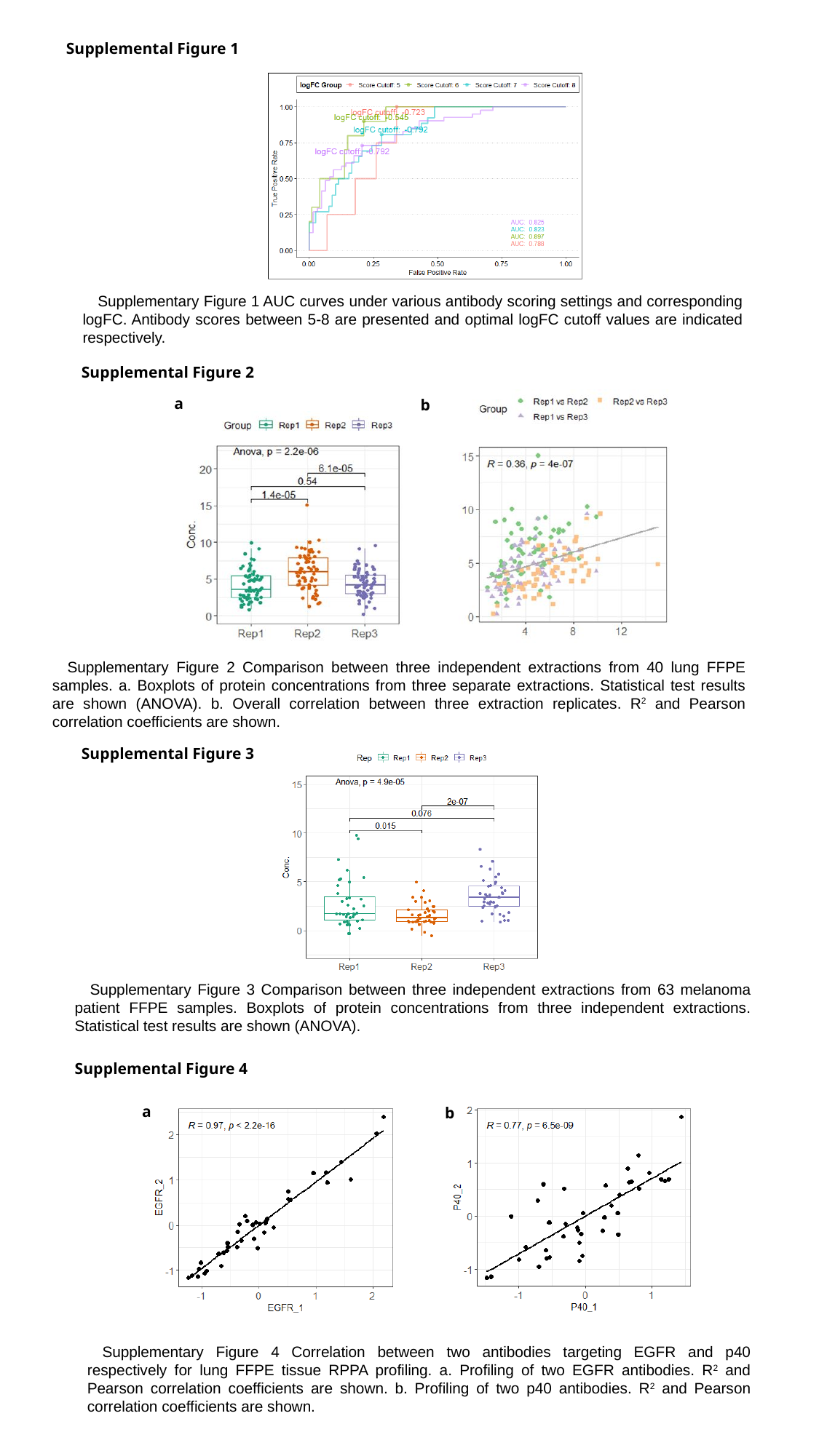

Supplemental Figure 1
Supplementary Figure 1 AUC curves under various antibody scoring settings and corresponding logFC. Antibody scores between 5-8 are presented and optimal logFC cutoff values are indicated respectively.
Supplemental Figure 2
a
b
Supplementary Figure 2 Comparison between three independent extractions from 40 lung FFPE samples. a. Boxplots of protein concentrations from three separate extractions. Statistical test results are shown (ANOVA). b. Overall correlation between three extraction replicates. R2 and Pearson correlation coefficients are shown.
Supplemental Figure 3
Supplementary Figure 3 Comparison between three independent extractions from 63 melanoma patient FFPE samples. Boxplots of protein concentrations from three independent extractions. Statistical test results are shown (ANOVA).
Supplemental Figure 4
a
b
Supplementary Figure 4 Correlation between two antibodies targeting EGFR and p40 respectively for lung FFPE tissue RPPA profiling. a. Profiling of two EGFR antibodies. R2 and Pearson correlation coefficients are shown. b. Profiling of two p40 antibodies. R2 and Pearson correlation coefficients are shown.
