# Supplementary material for: A reverse phase protein array based phospho-antibody characterization approach and its applicability for clinical derived tissue specimens
Source: Sci Rep. 2022 Dec 26;12:22373. doi: 10.1038/s41598-022-26715-9 (PMC9792559; doi:10.1038/s41598-022-26715-9)
Supplement: Supplementary file 4 — Supplementary Information 3. [file 41598_2022_26715_MOESM4_ESM.pptx]

## Slide 1
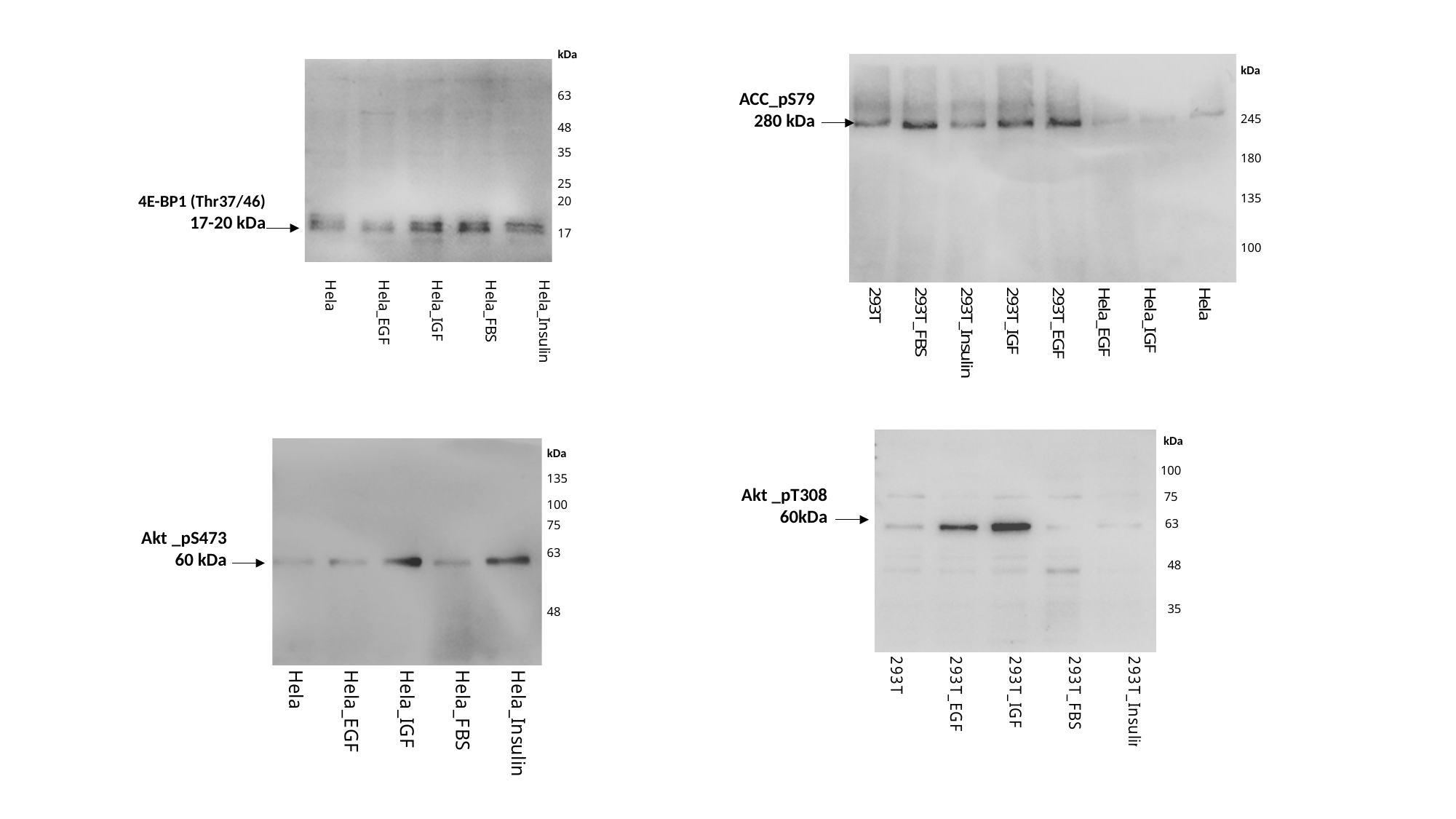

kDa
63
48
35
25
4E-BP1 (Thr37/46)
17-20 kDa
20
17
kDa
ACC_pS79
280 kDa
245
180
135
100
kDa
Akt _pT308
60kDa
75
63
48
35
kDa
135
100
75
Akt _pS473
60 kDa
63
48
100

## Slide 2
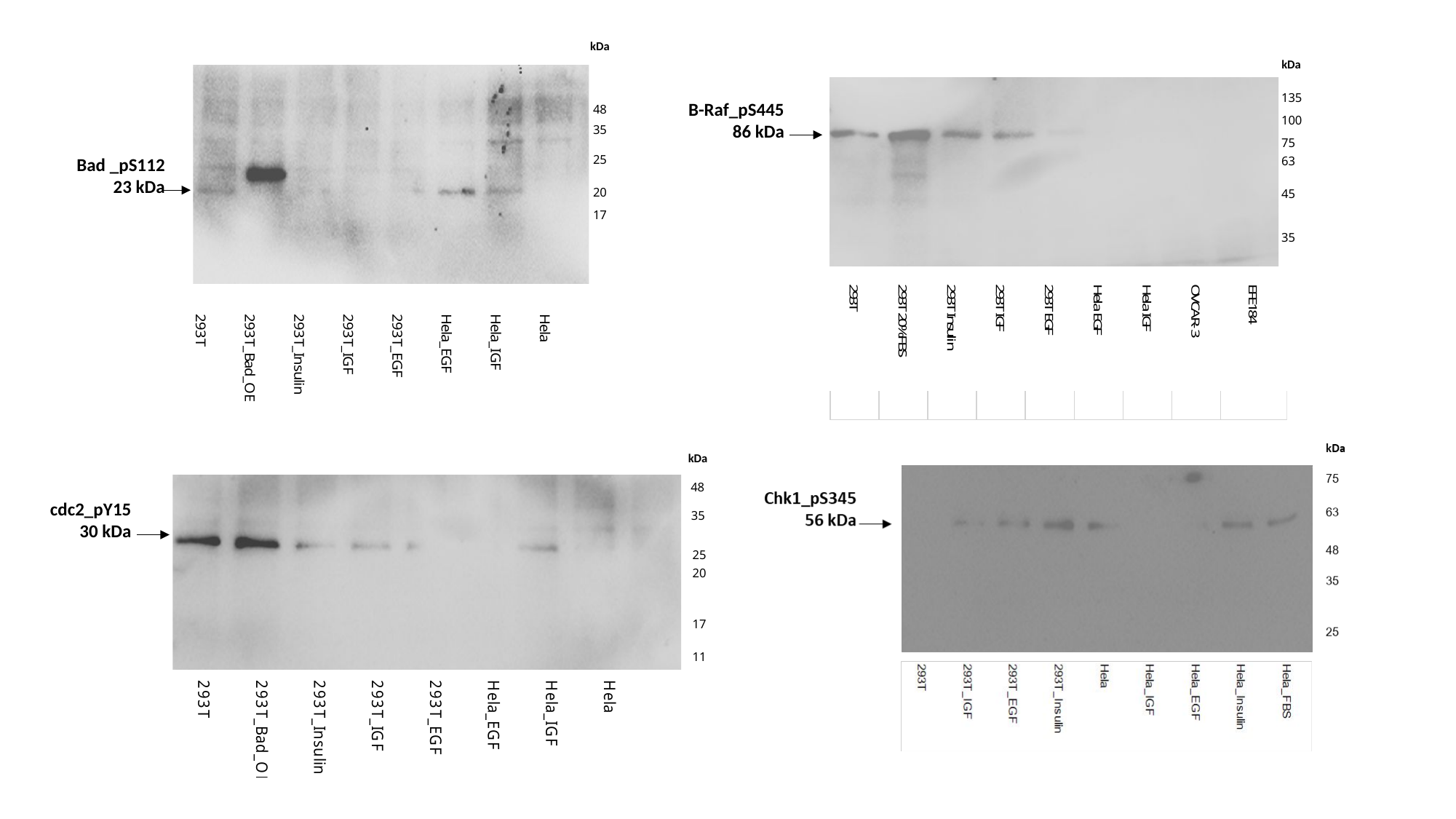

kDa
48
35
25
Bad _pS112
23 kDa
20
17
kDa
135
B-Raf_pS445
86 kDa
100
75
63
45
35
kDa
48
cdc2_pY15
30 kDa
35
25
20
17
11

## Slide 3
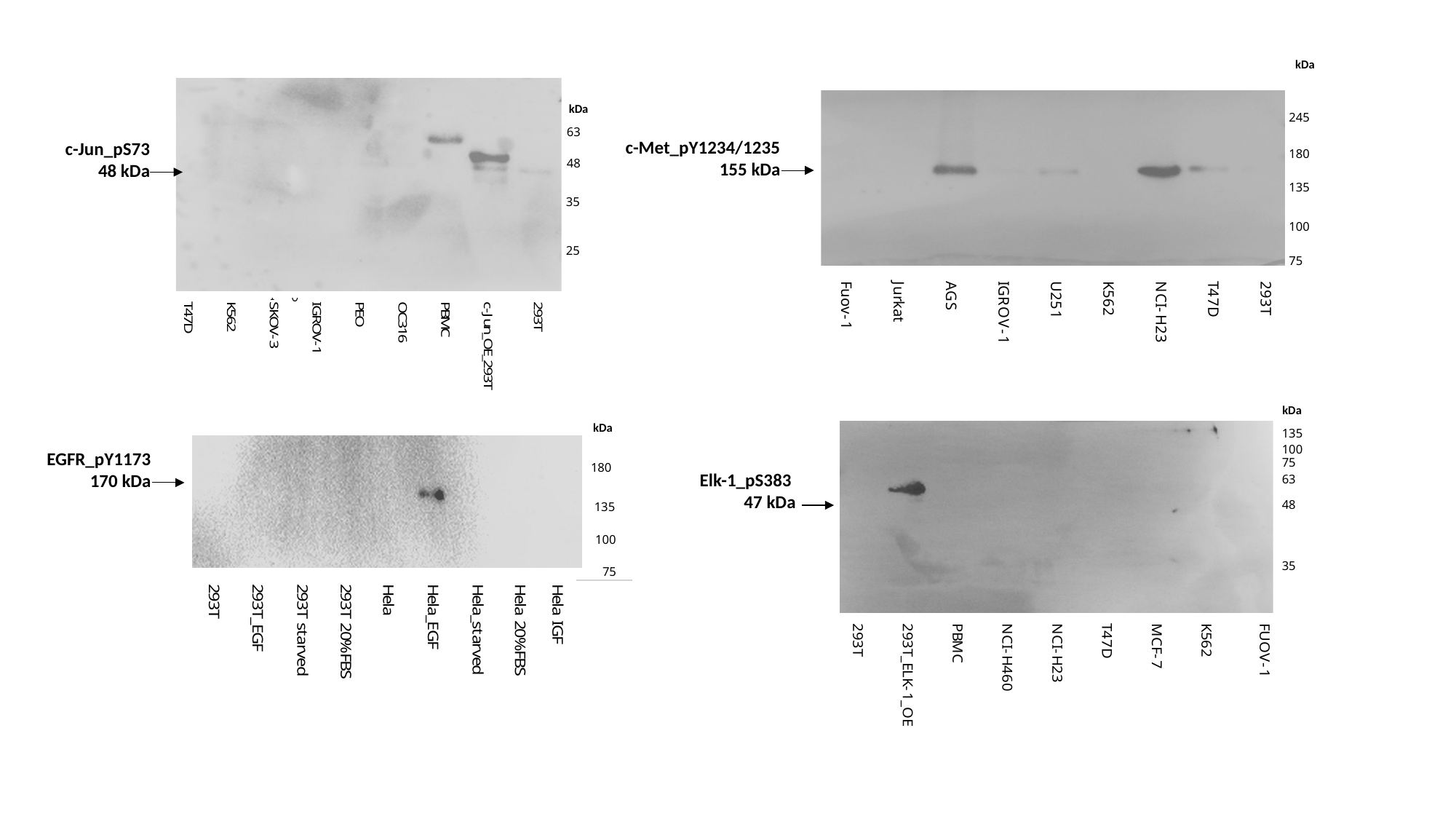

kDa
245
c-Met_pY1234/1235
155 kDa
180
135
100
75
kDa
63
c-Jun_pS73
48 kDa
48
35
25
kDa
135
100
75
Elk-1_pS383
47 kDa
63
48
35
kDa
180
135
100
75
EGFR_pY1173
170 kDa

## Slide 4
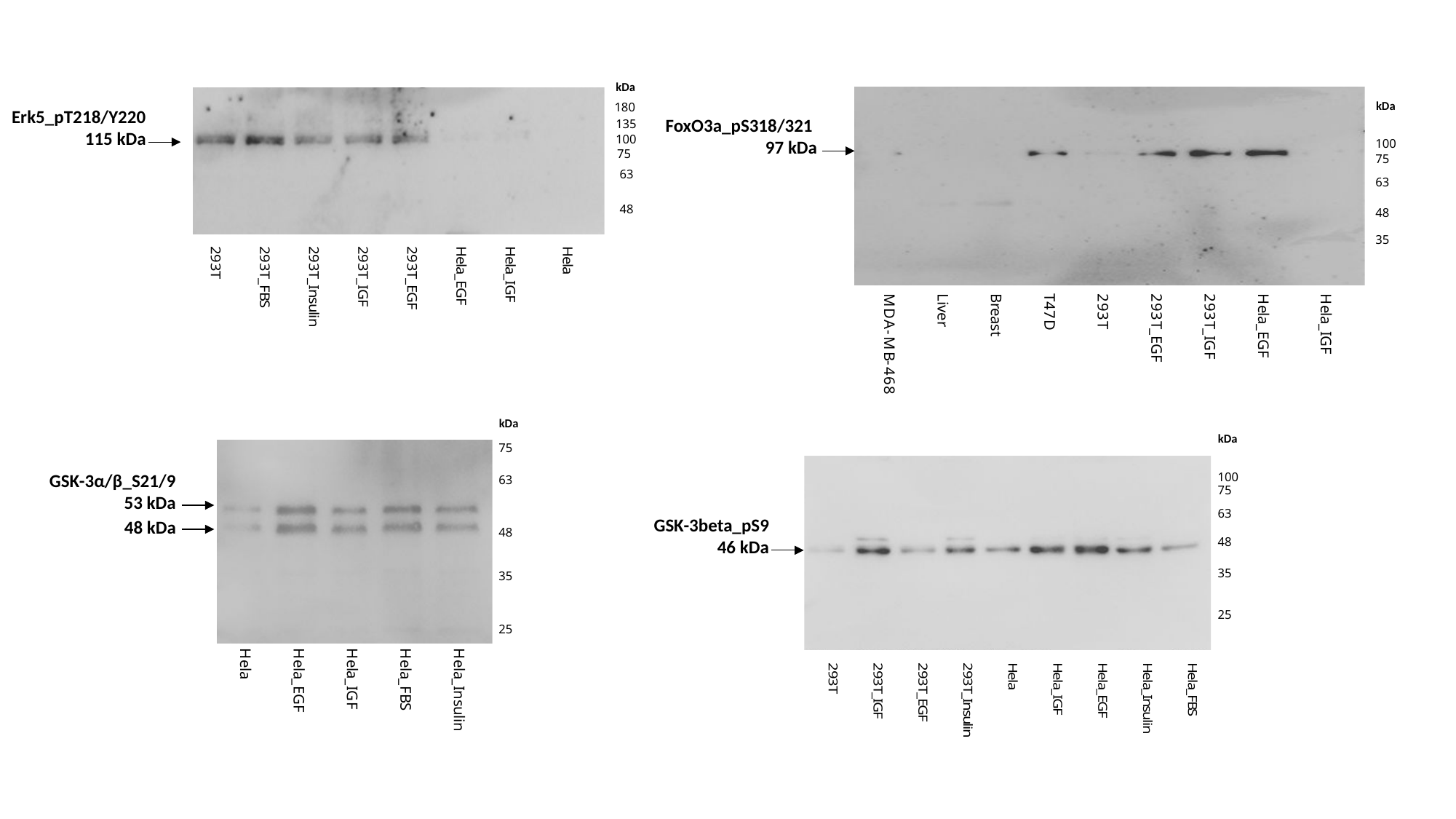

kDa
180
Erk5_pT218/Y220
115 kDa
135
100
75
63
48
kDa
100
75
63
48
35
FoxO3a_pS318/321
97 kDa
kDa
75
GSK-3α/β_S21/9
53 kDa
63
48 kDa
48
35
25
kDa
100
75
63
GSK-3beta_pS9
46 kDa
48
35
25

## Slide 5
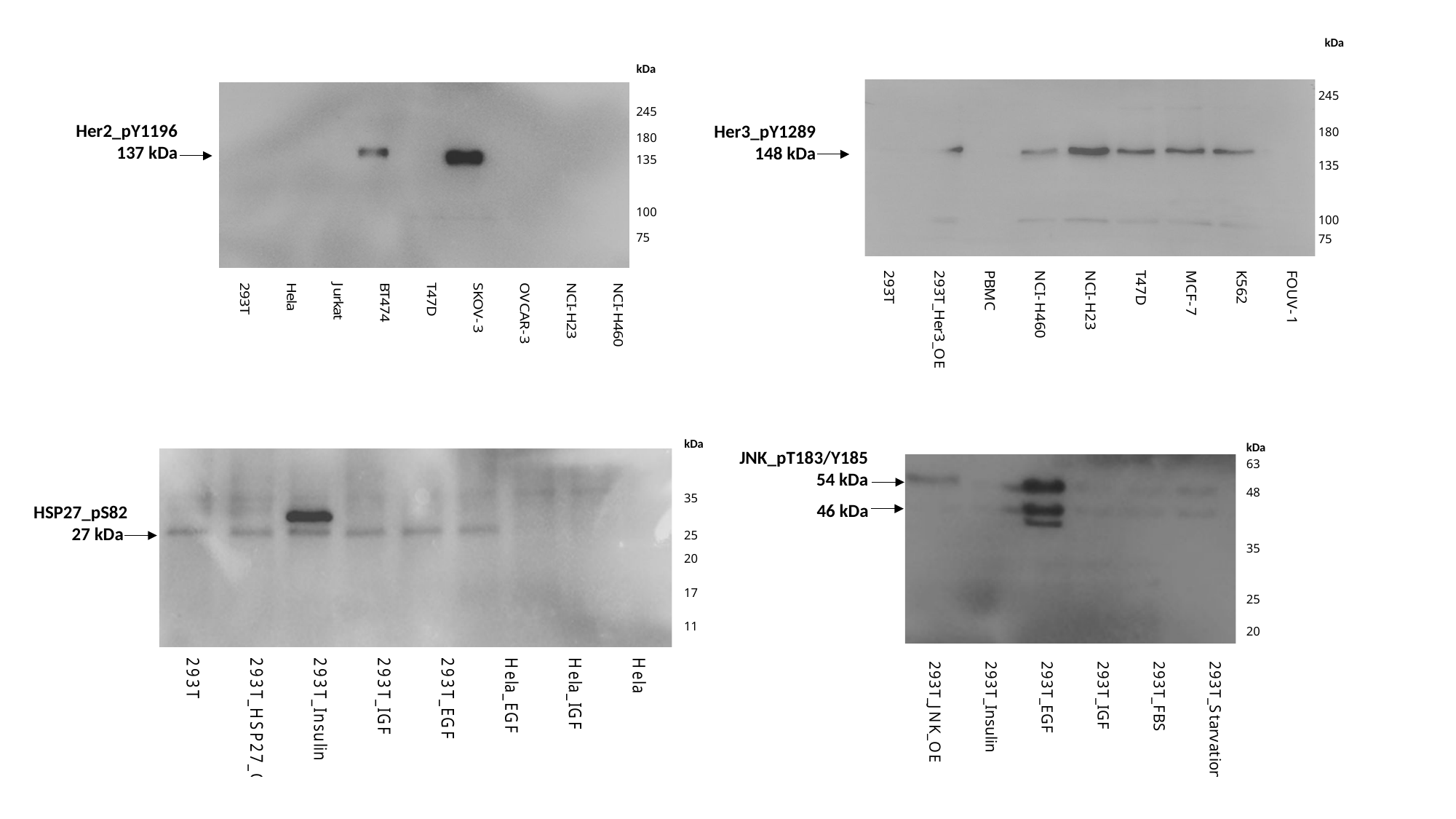

kDa
245
Her3_pY1289
148 kDa
180
135
100
75
kDa
245
Her2_pY1196
137 kDa
180
135
100
75
kDa
35
HSP27_pS82
27 kDa
25
20
17
11
kDa
JNK_pT183/Y185
54 kDa
63
48
46 kDa
35
25
20

## Slide 6
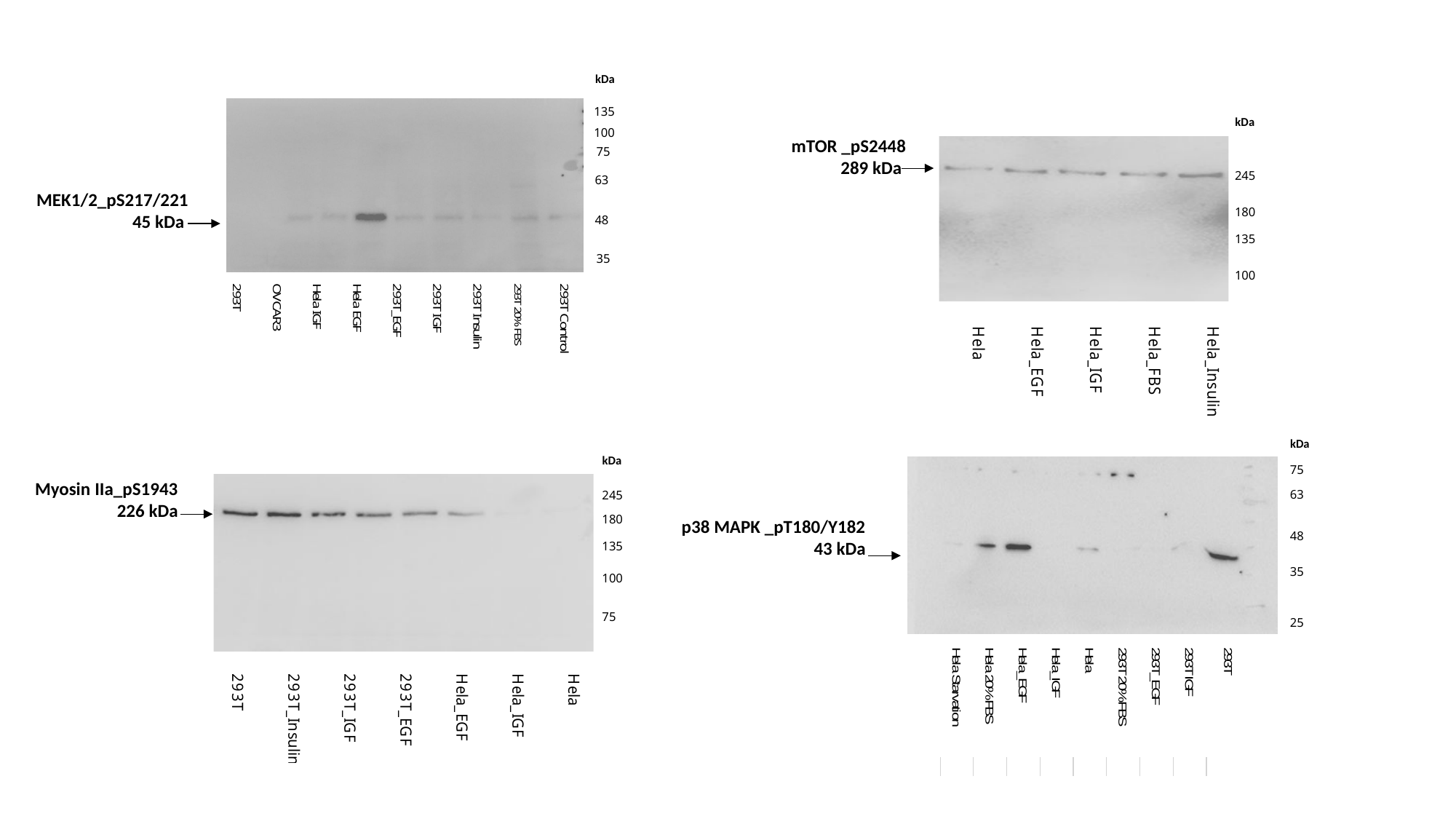

kDa
135
100
75
63
MEK1/2_pS217/221
45 kDa
48
35
kDa
mTOR _pS2448
289 kDa
245
180
135
100
kDa
75
63
p38 MAPK _pT180/Y182
43 kDa
48
35
25
kDa
Myosin IIa_pS1943
226 kDa
245
180
135
100
75

## Slide 7
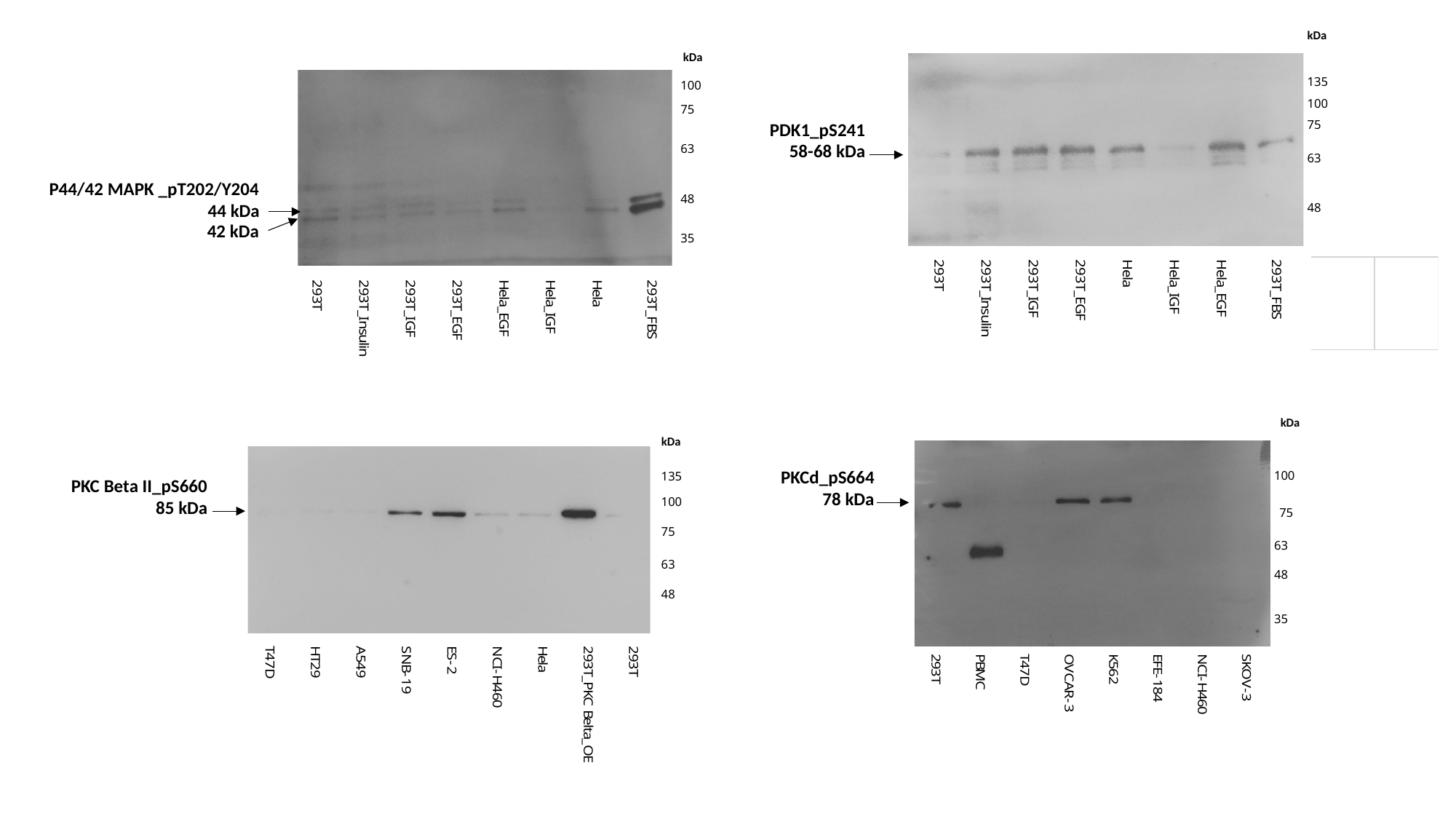

kDa
135
100
75
PDK1_pS241
58-68 kDa
63
48
kDa
100
75
63
P44/42 MAPK _pT202/Y204
44 kDa
48
42 kDa
35
kDa
PKCd_pS664
78 kDa
100
75
63
48
35
kDa
135
PKC Beta II_pS660
85 kDa
100
75
63
48

## Slide 8
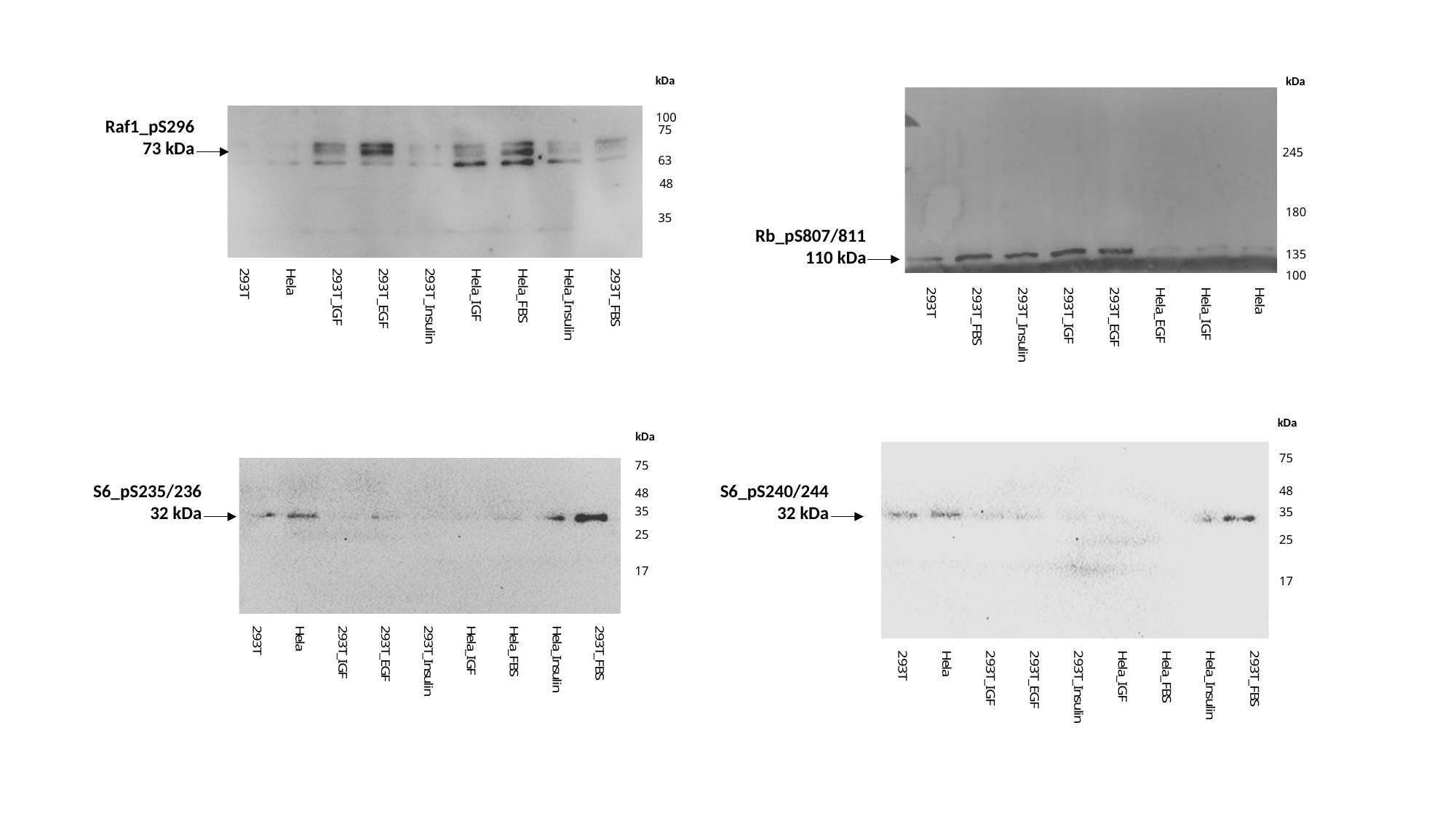

kDa
100
Raf1_pS296
73 kDa
75
63
48
35
kDa
245
180
Rb_pS807/811
110 kDa
135
100
kDa
75
S6_pS240/244
32 kDa
48
35
25
17
kDa
75
48
35
25
17
S6_pS235/236
32 kDa

## Slide 9
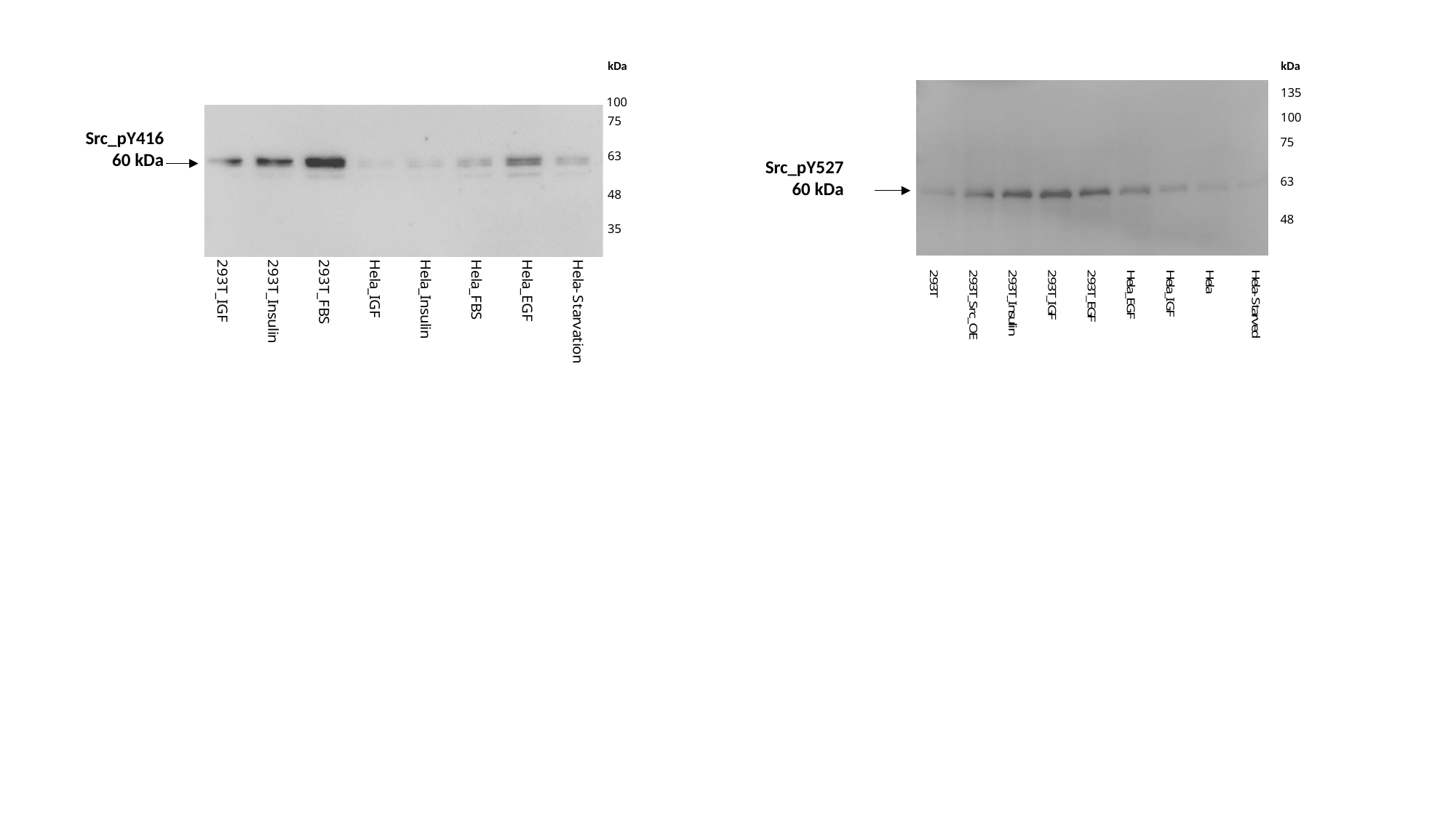

kDa
100
75
Src_pY416
60 kDa
63
48
35
kDa
135
100
75
Src_pY527
60 kDa
63
48

## Slide 10
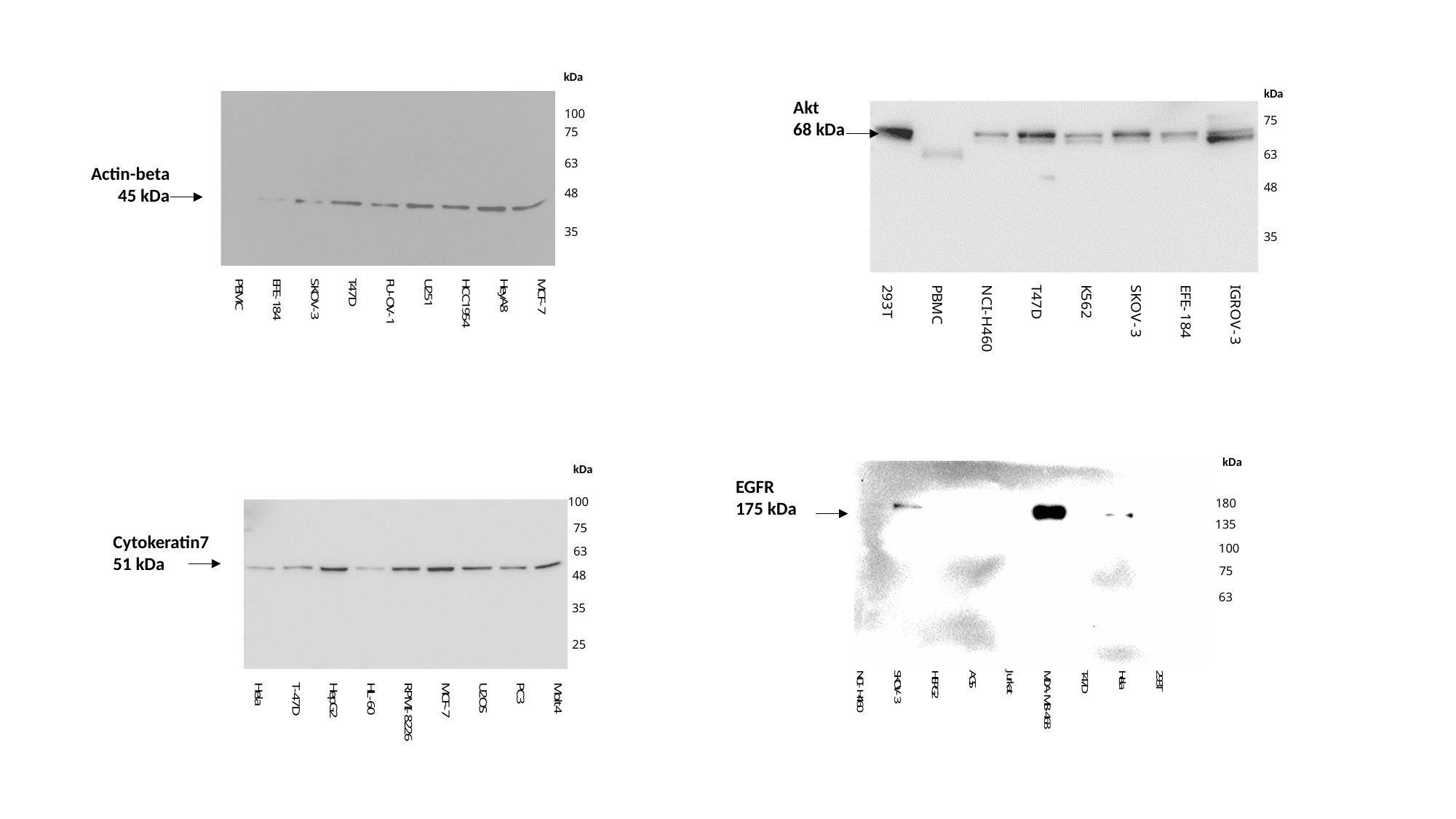

kDa
100
75
63
Actin-beta
45 kDa
48
35
kDa
Akt
68 kDa
75
63
48
35
kDa
EGFR
175 kDa
180
135
100
75
63
kDa
100
75
Cytokeratin7
51 kDa
63
48
35
25

## Slide 11
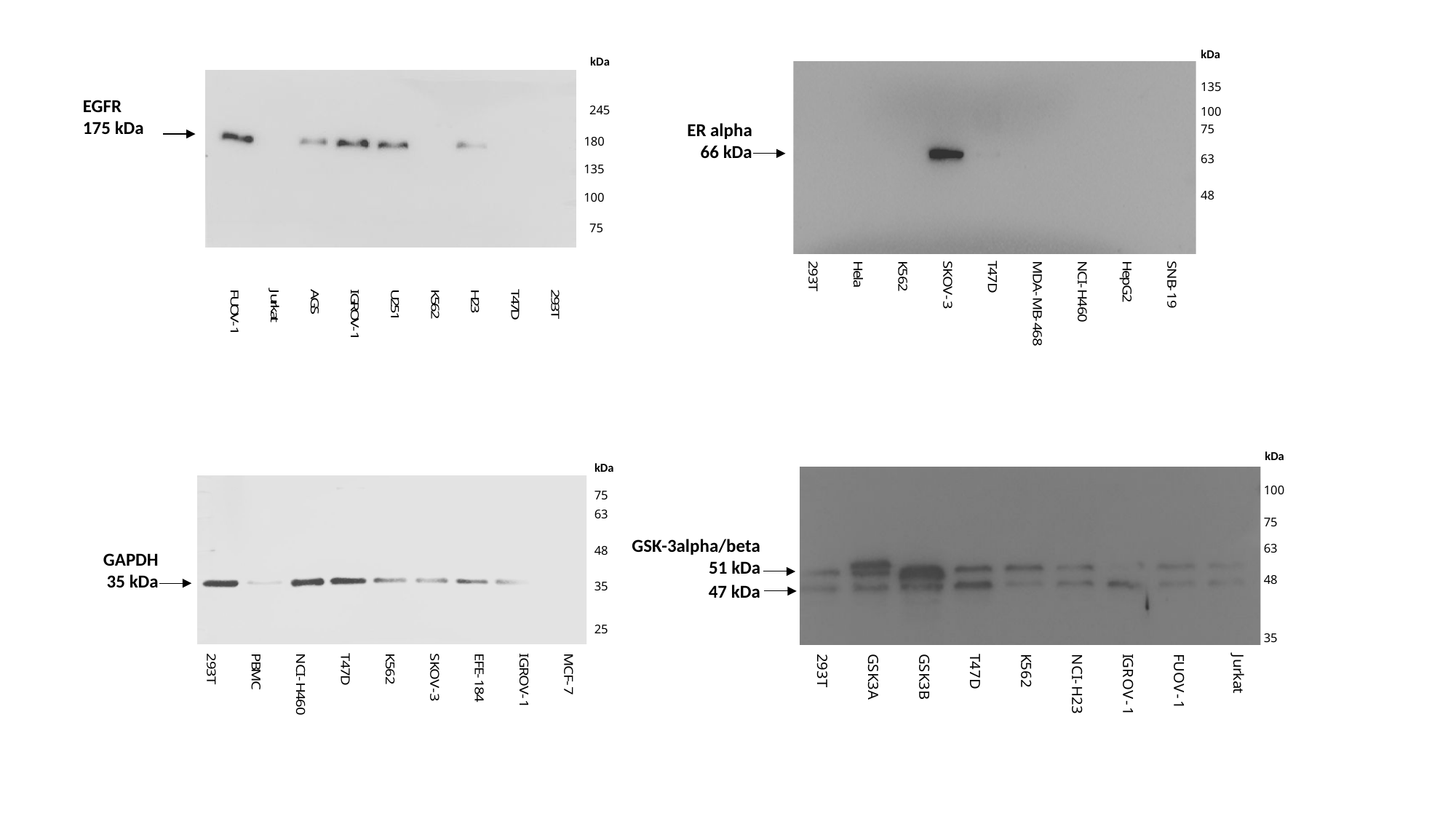

kDa
135
100
75
63
48
ER alpha
66 kDa
kDa
EGFR
175 kDa
245
180
135
100
75
kDa
100
75
GSK-3alpha/beta
51 kDa
63
48
47 kDa
35
kDa
75
63
48
GAPDH
35 kDa
35
25

## Slide 12
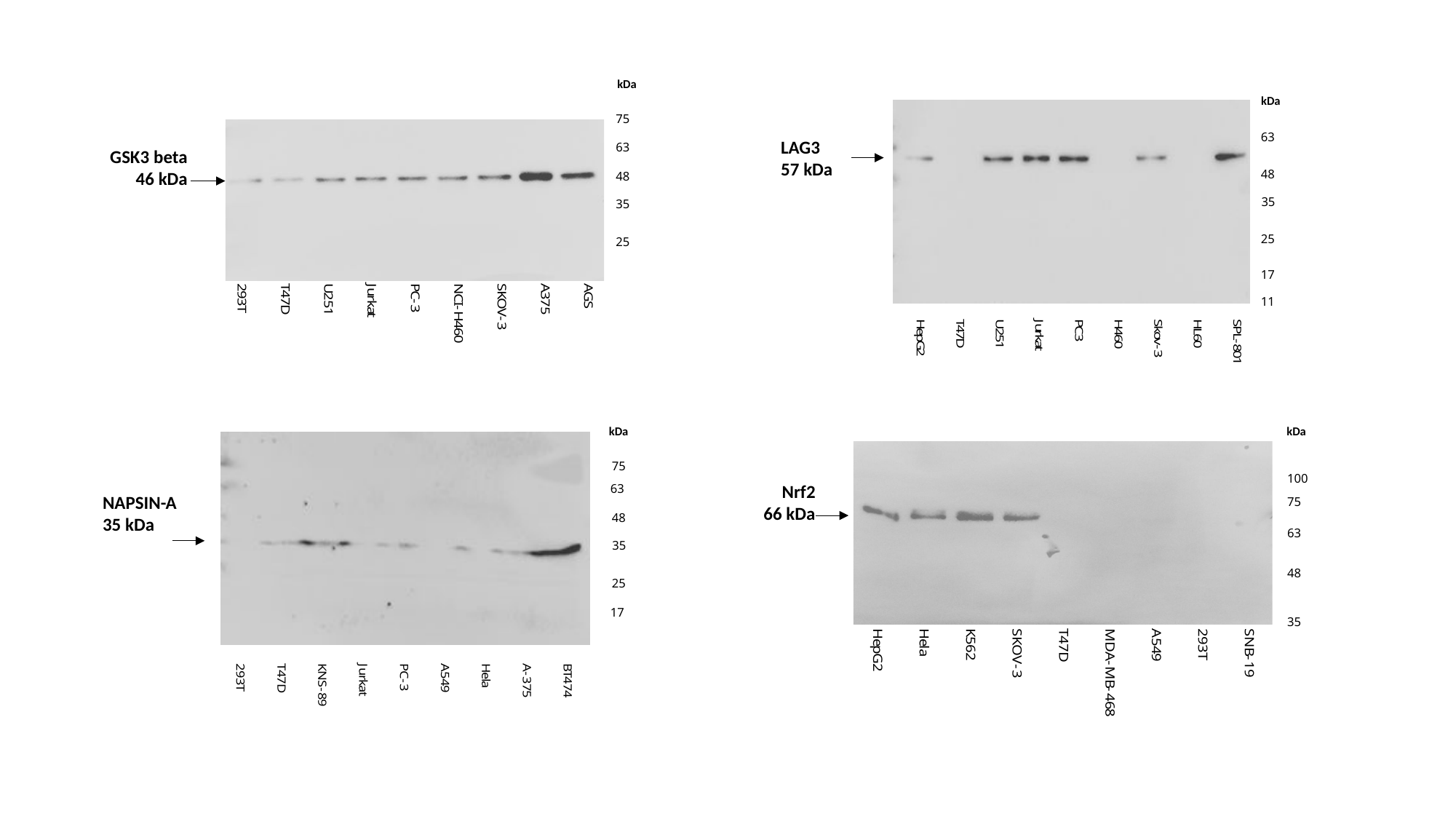

kDa
75
63
GSK3 beta
46 kDa
48
35
25
kDa
63
LAG3
57 kDa
48
35
25
17
11
kDa
75
63
NAPSIN-A
35 kDa
48
35
25
17
kDa
100
Nrf2
66 kDa
75
63
48
35

## Slide 13
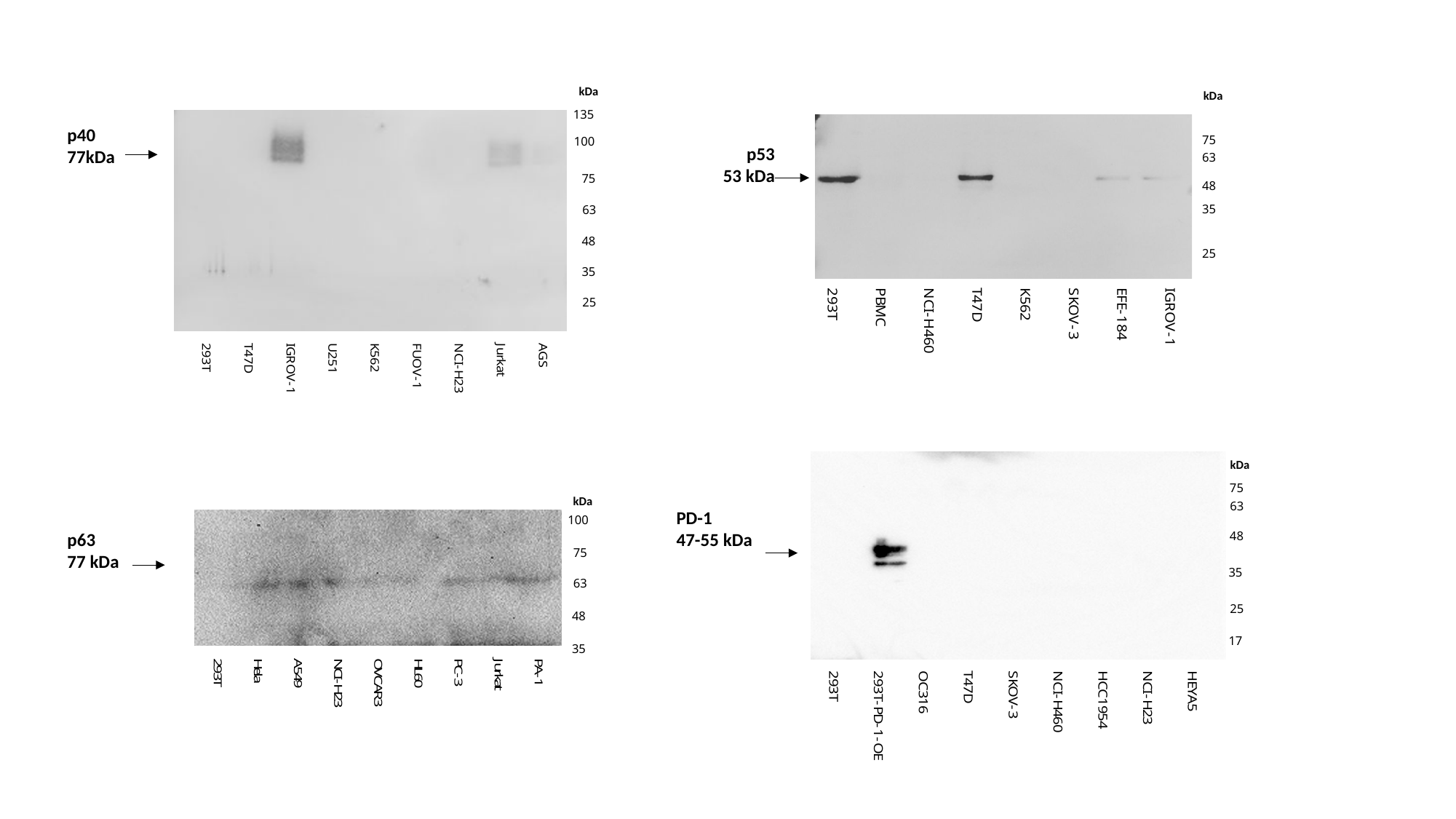

kDa
135
p40
77kDa
100
75
63
48
35
25
kDa
75
63
48
35
25
p53
53 kDa
kDa
75
63
PD-1
47-55 kDa
48
35
25
17
kDa
100
p63
77 kDa
75
63
48
35

## Slide 14
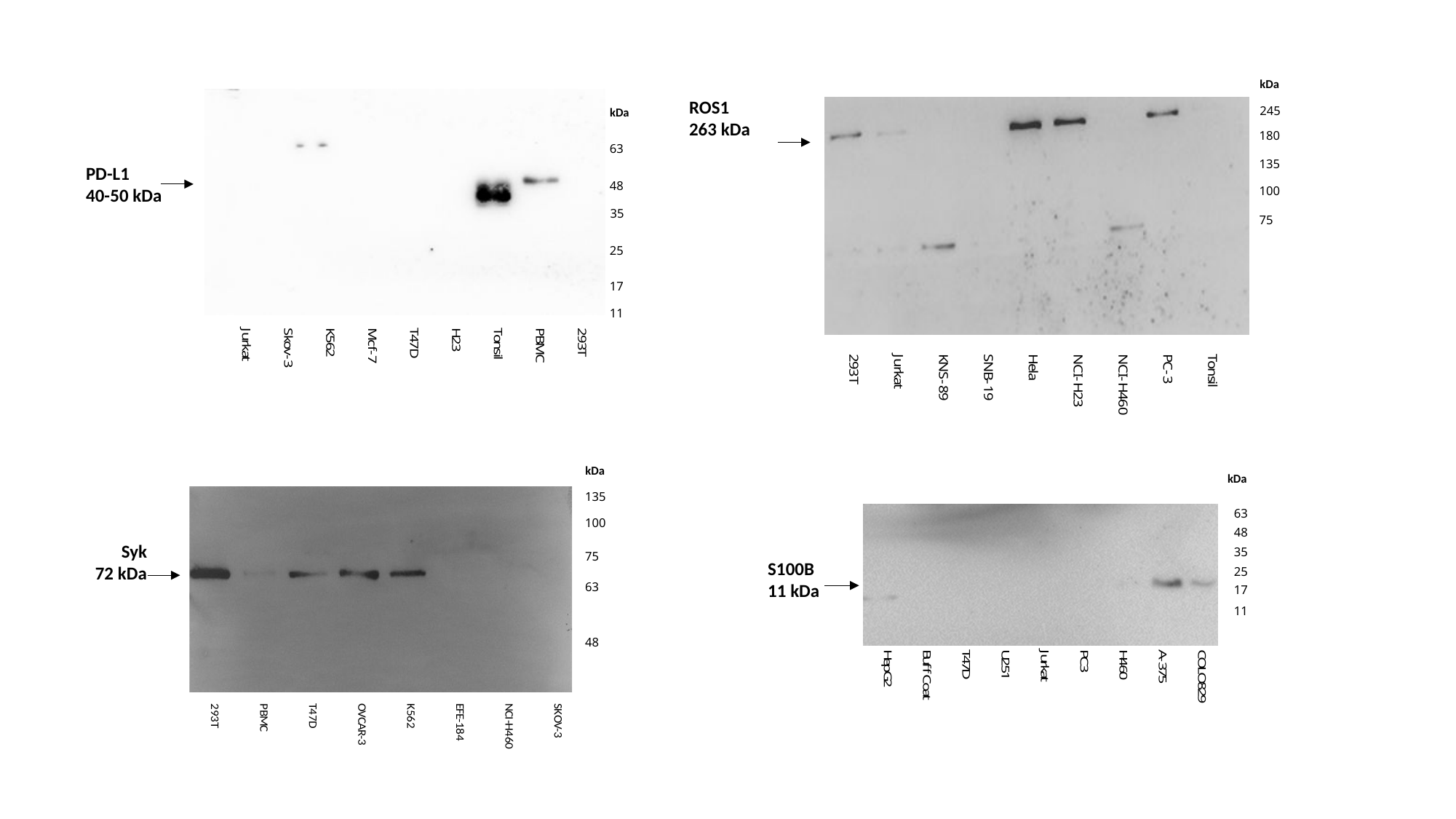

kDa
ROS1
263 kDa
245
180
135
100
75
kDa
63
PD-L1
40-50 kDa
48
35
25
17
11
kDa
135
100
Syk
72 kDa
75
63
48
kDa
63
48
35
S100B
11 kDa
25
17
11

## Slide 15
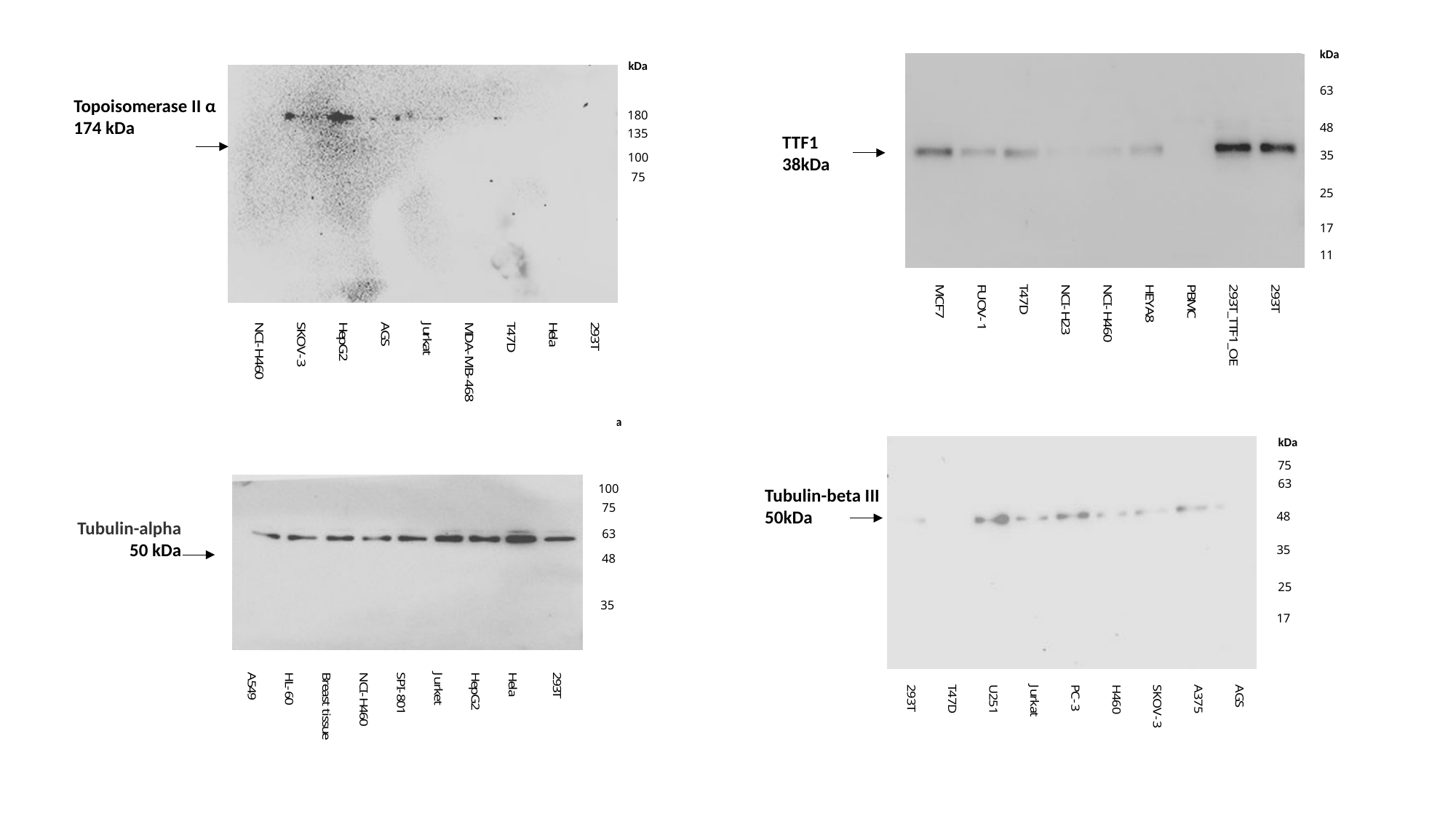

kDa
63
48
TTF1
38kDa
35
25
17
11
kDa
Topoisomerase II α
174 kDa
180
135
100
75
kDa
100
75
63
48
35
Tubulin-alpha
50 kDa
kDa
75
63
Tubulin-beta III
50kDa
48
35
25
17

## Slide 16
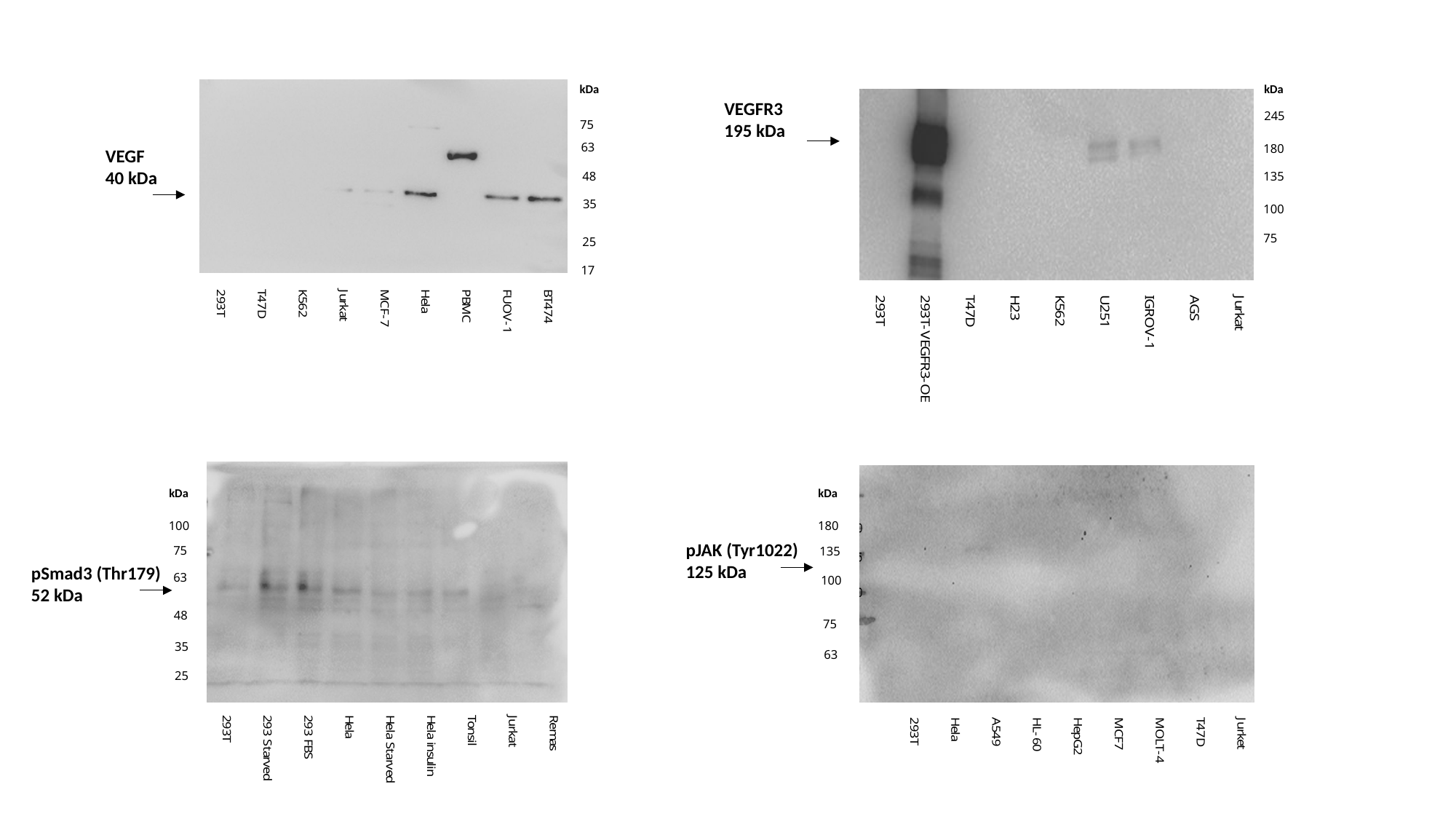

kDa
75
63
VEGF
40 kDa
48
35
25
17
kDa
VEGFR3
195 kDa
245
180
135
100
75
kDa
kDa
100
180
pJAK (Tyr1022)
125 kDa
75
135
pSmad3 (Thr179)
52 kDa
63
100
48
75
35
63
25

## Slide 17
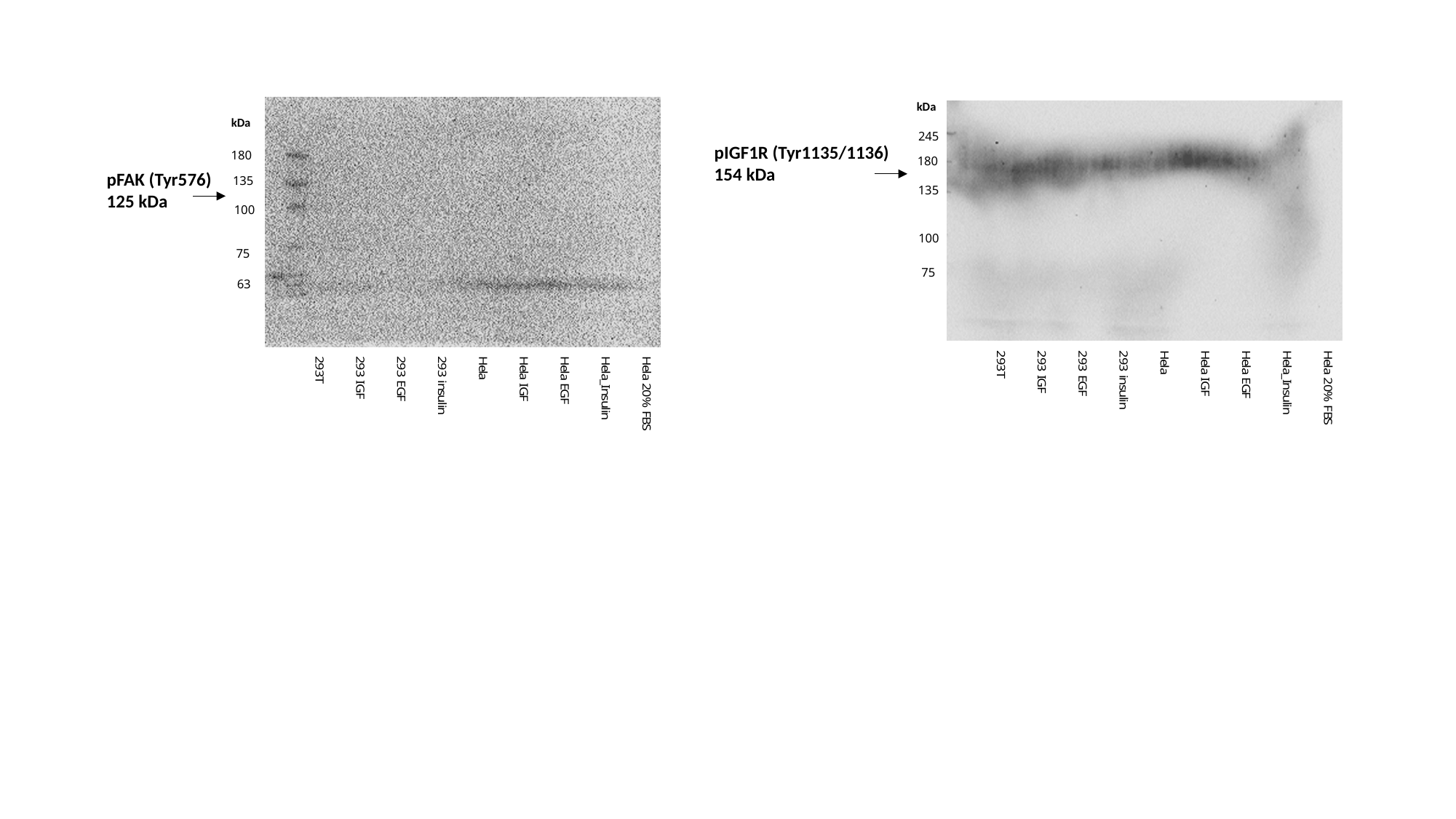

kDa
kDa
245
pIGF1R (Tyr1135/1136)
154 kDa
180
180
pFAK (Tyr576)
125 kDa
135
135
100
100
75
75
63

## Slide 18
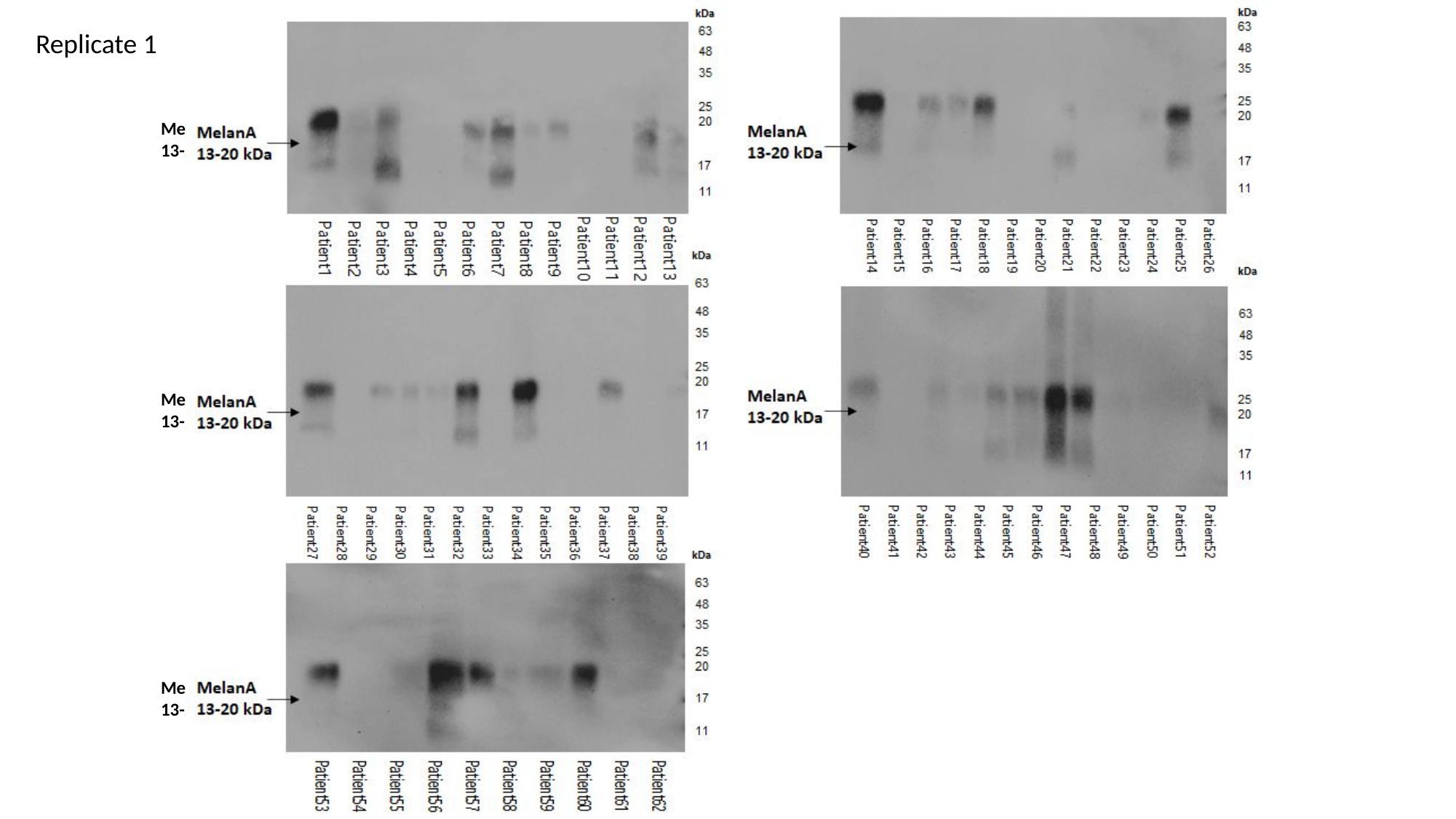

kDa
kDa
63
63
Replicate 1
48
48
35
35
25
25
20
20
MelanA
13-20 kDa
MelanA
13-20 kDa
17
17
11
11
kDa
kDa
63
48
63
35
48
35
25
20
MelanA
13-20 kDa
MelanA
13-20 kDa
25
17
20
11
17
11
kDa
63
48
35
25
20
MelanA
13-20 kDa
17
11

## Slide 19
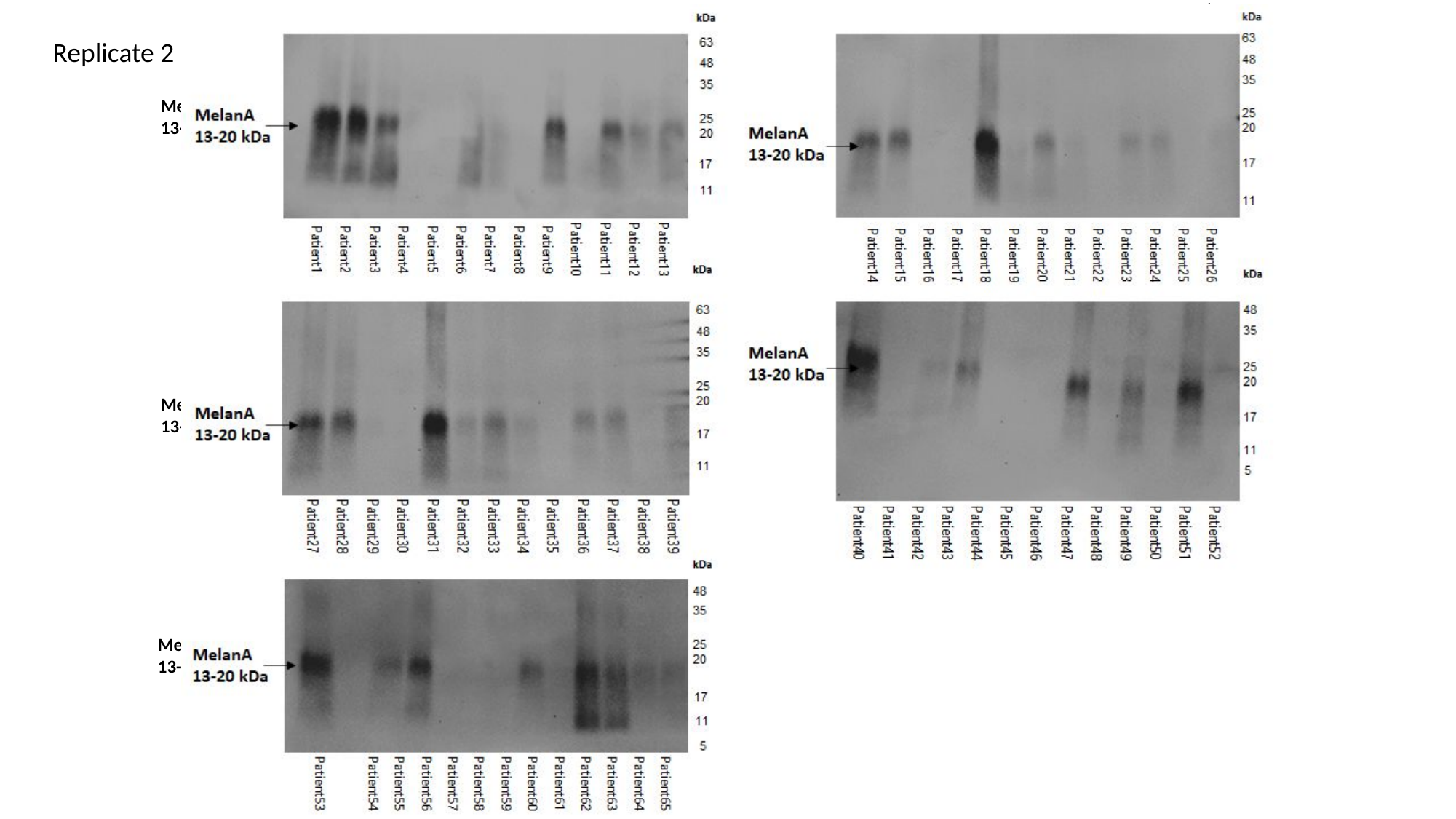

kDa
kDa
63
63
Replicate 2
48
48
35
35
MelanA
13-20 kDa
25
25
20
MelanA
13-20 kDa
20
17
17
11
11
kDa
kDa
48
63
35
48
MelanA
13-20 kDa
35
25
20
25
20
MelanA
13-20 kDa
17
17
11
11
5
kDa
48
35
25
MelanA
13-20 kDa
20
17
11
5

## Slide 20
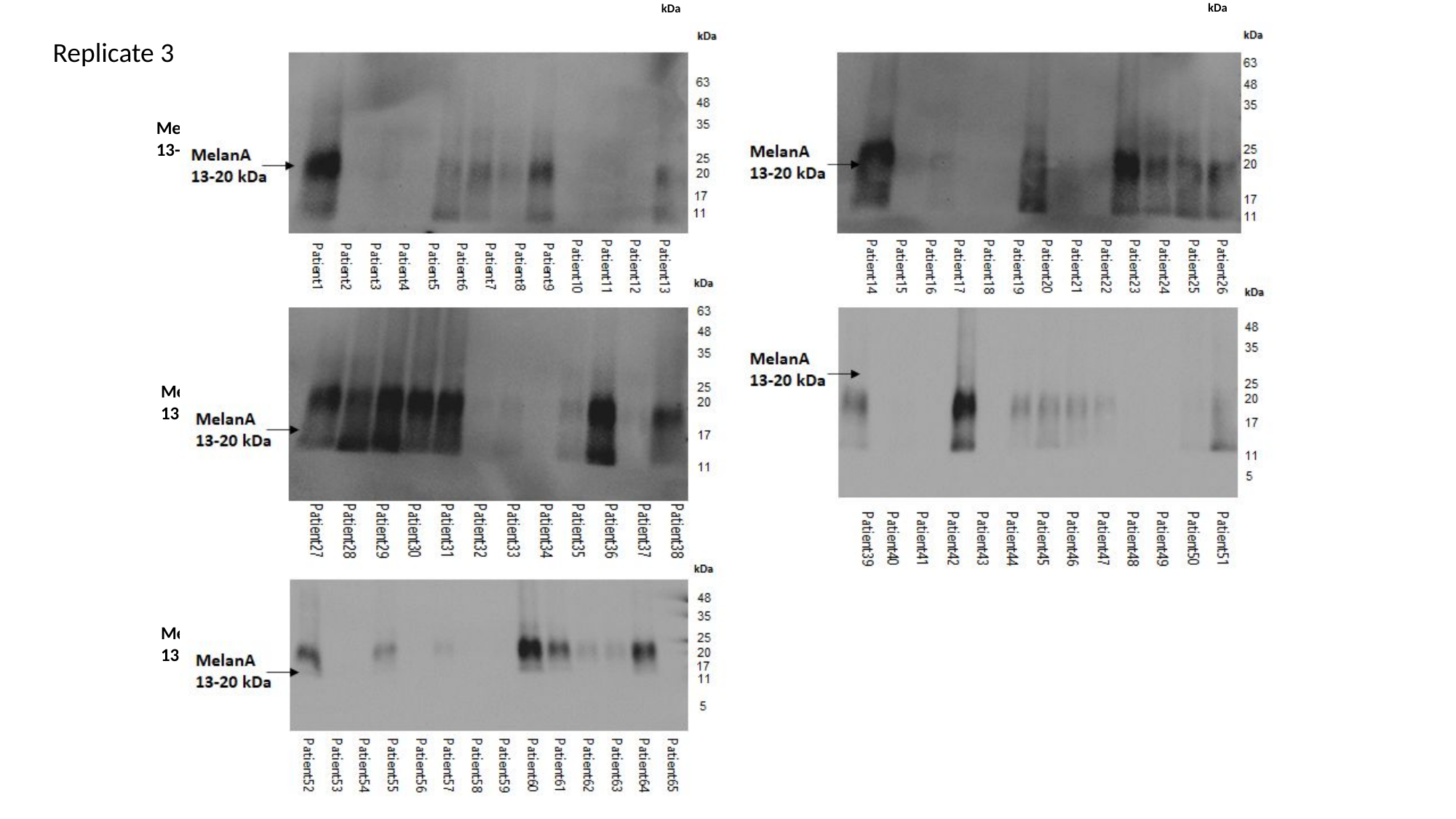

kDa
kDa
63
Replicate 3
63
48
48
35
35
MelanA
13-20 kDa
25
MelanA
13-20 kDa
25
20
20
17
17
11
11
kDa
kDa
63
48
48
35
35
MelanA
13-20 kDa
25
25
20
20
MelanA
13-20 kDa
17
17
11
11
5
kDa
48
35
25
20
MelanA
13-20 kDa
17
11
5
