# Supplementary material for: A reverse phase protein array based phospho-antibody characterization approach and its applicability for clinical derived tissue specimens
Source: Sci Rep. 2022 Dec 26;12:22373. doi: 10.1038/s41598-022-26715-9 (PMC9792559; doi:10.1038/s41598-022-26715-9)
Supplement: Supplementary file 7 — Supplementary Information 6. [file 41598_2022_26715_MOESM7_ESM.pptx]

## Slide 1
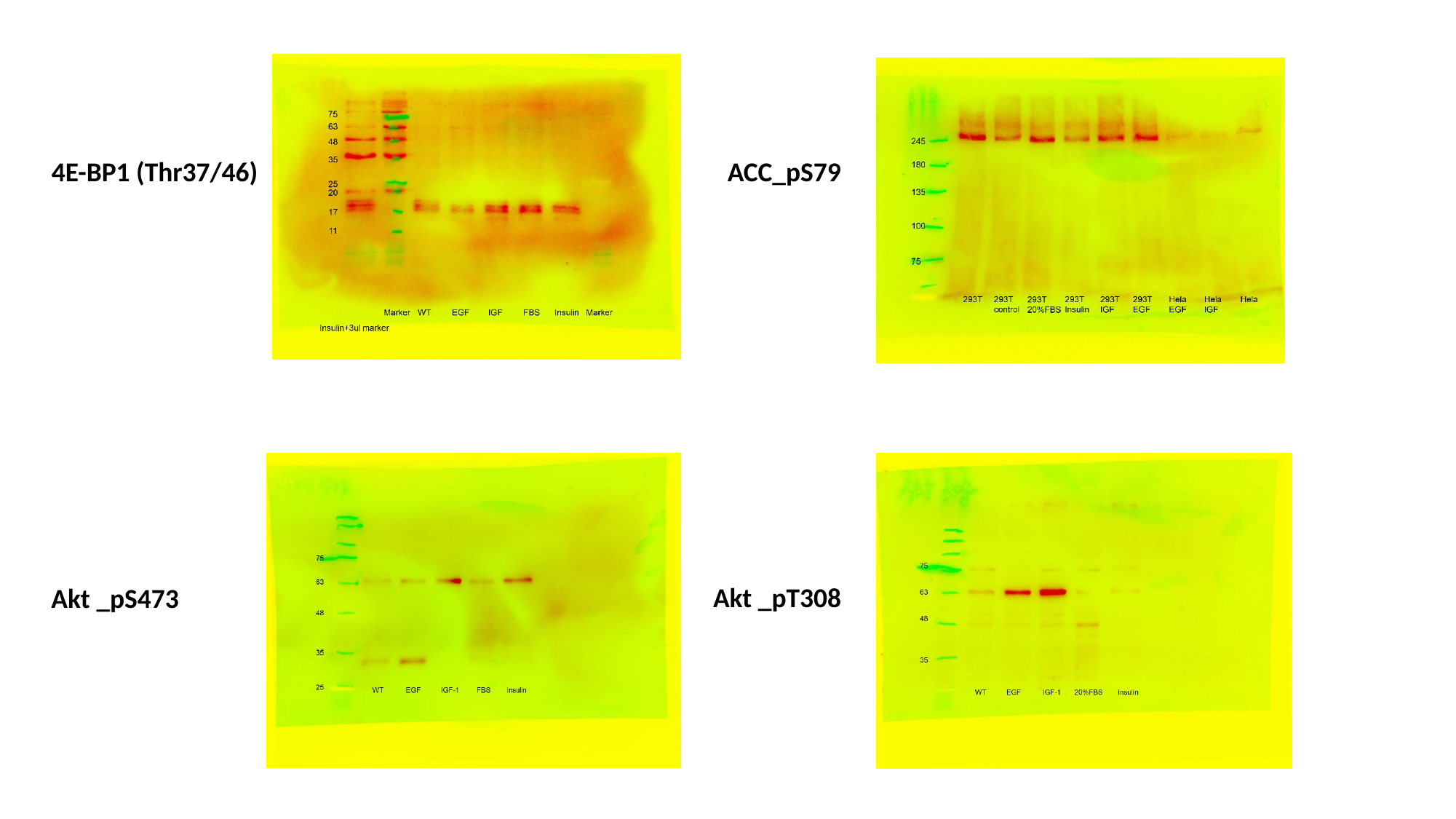

4E-BP1 (Thr37/46)
ACC_pS79
Akt _pT308
Akt _pS473

## Slide 2
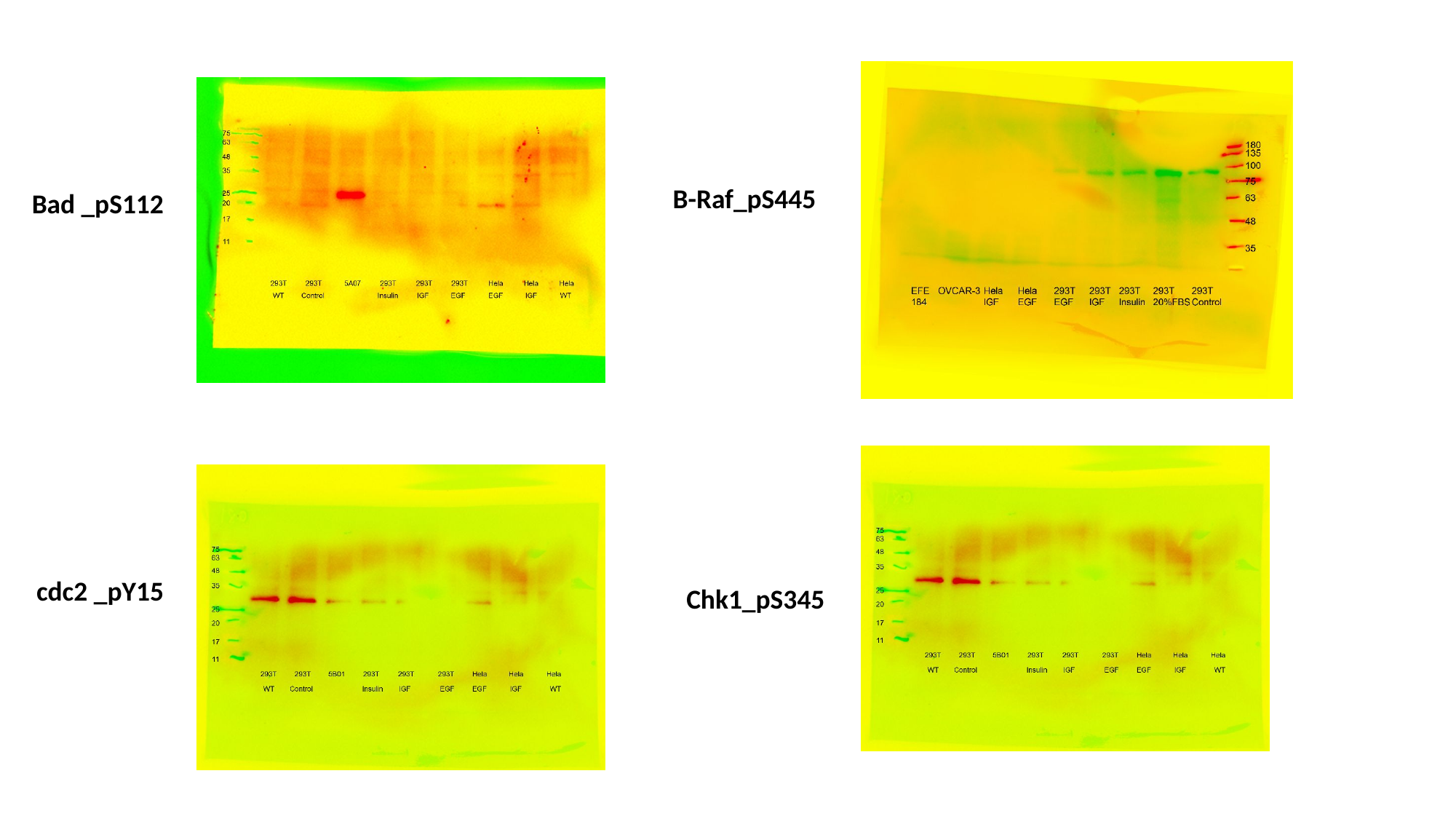

B-Raf_pS445
Bad _pS112
cdc2 _pY15
Chk1_pS345

## Slide 3
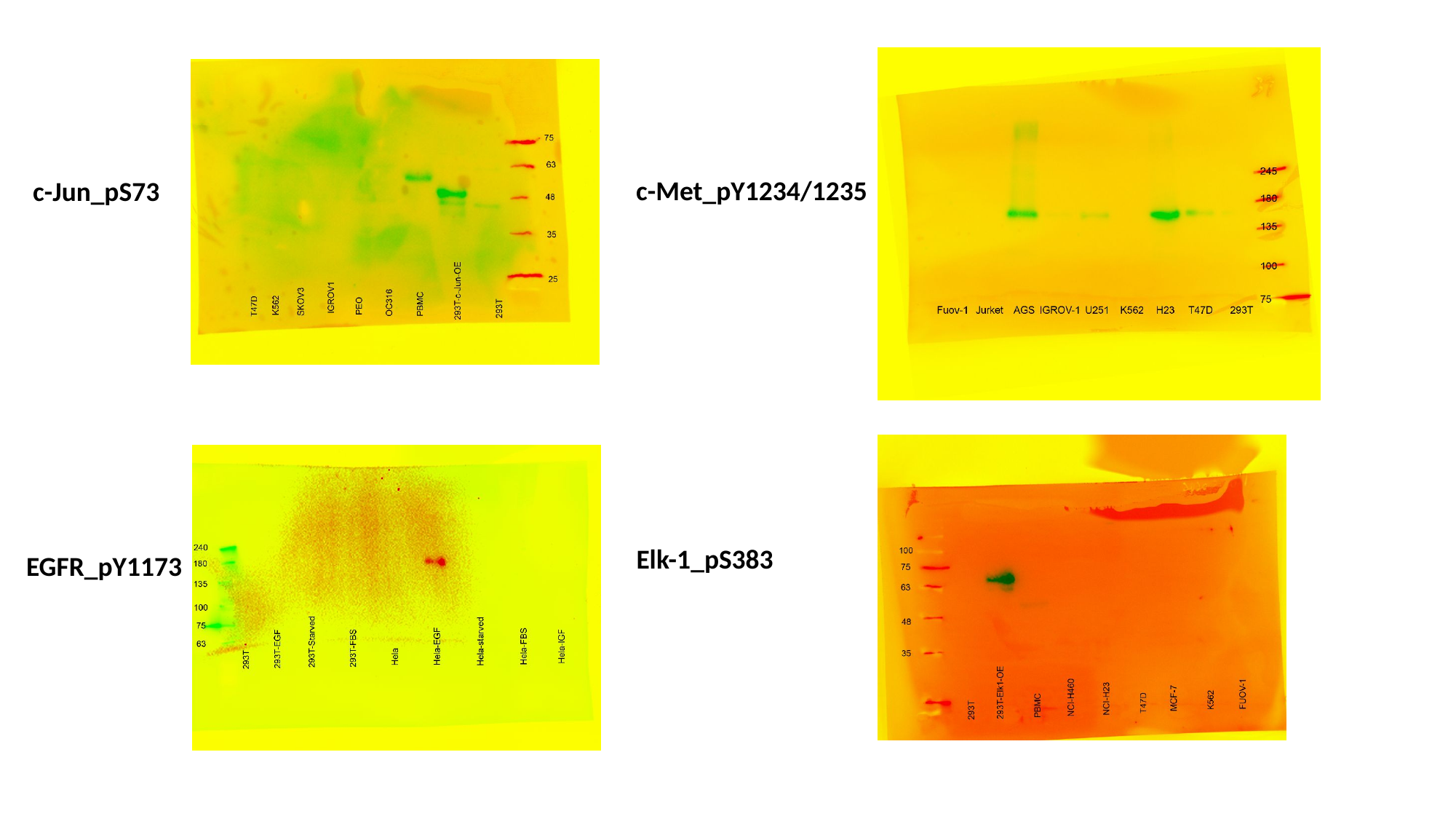

c-Met_pY1234/1235
c-Jun_pS73
Elk-1_pS383
EGFR_pY1173

## Slide 4
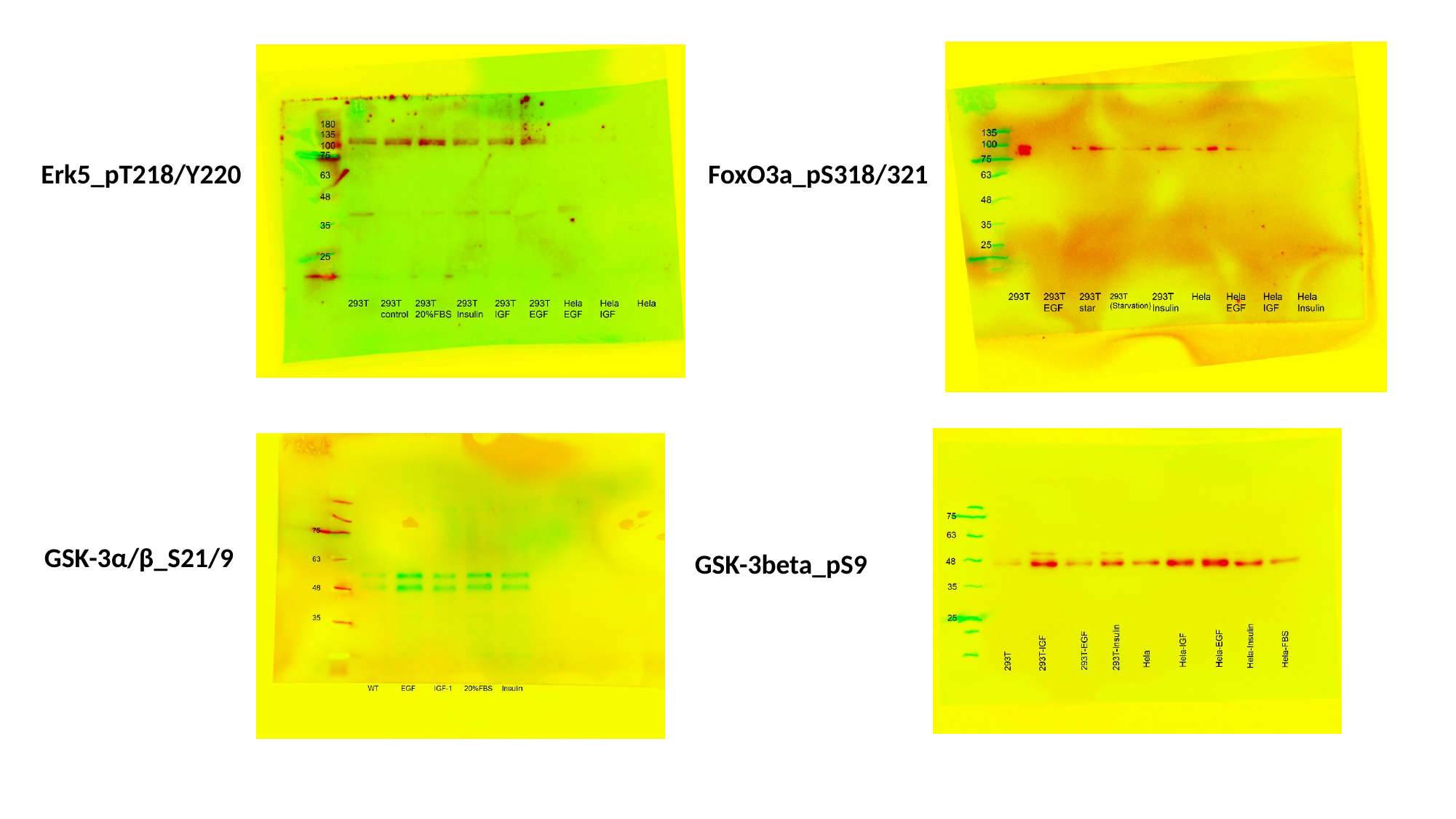

Erk5_pT218/Y220
FoxO3a_pS318/321
GSK-3α/β_S21/9
GSK-3beta_pS9

## Slide 5
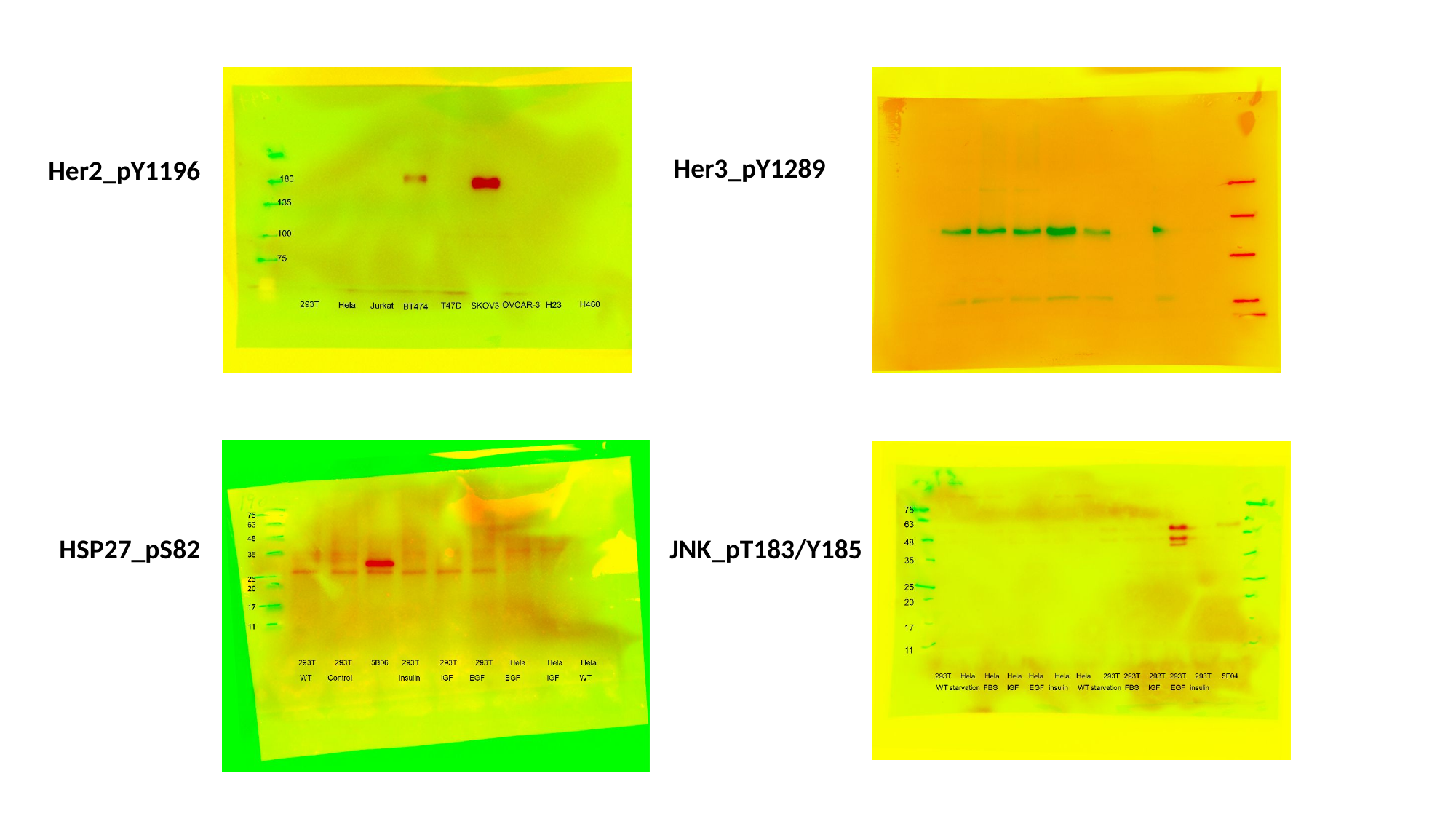

Her3_pY1289
Her2_pY1196
HSP27_pS82
JNK_pT183/Y185

## Slide 6
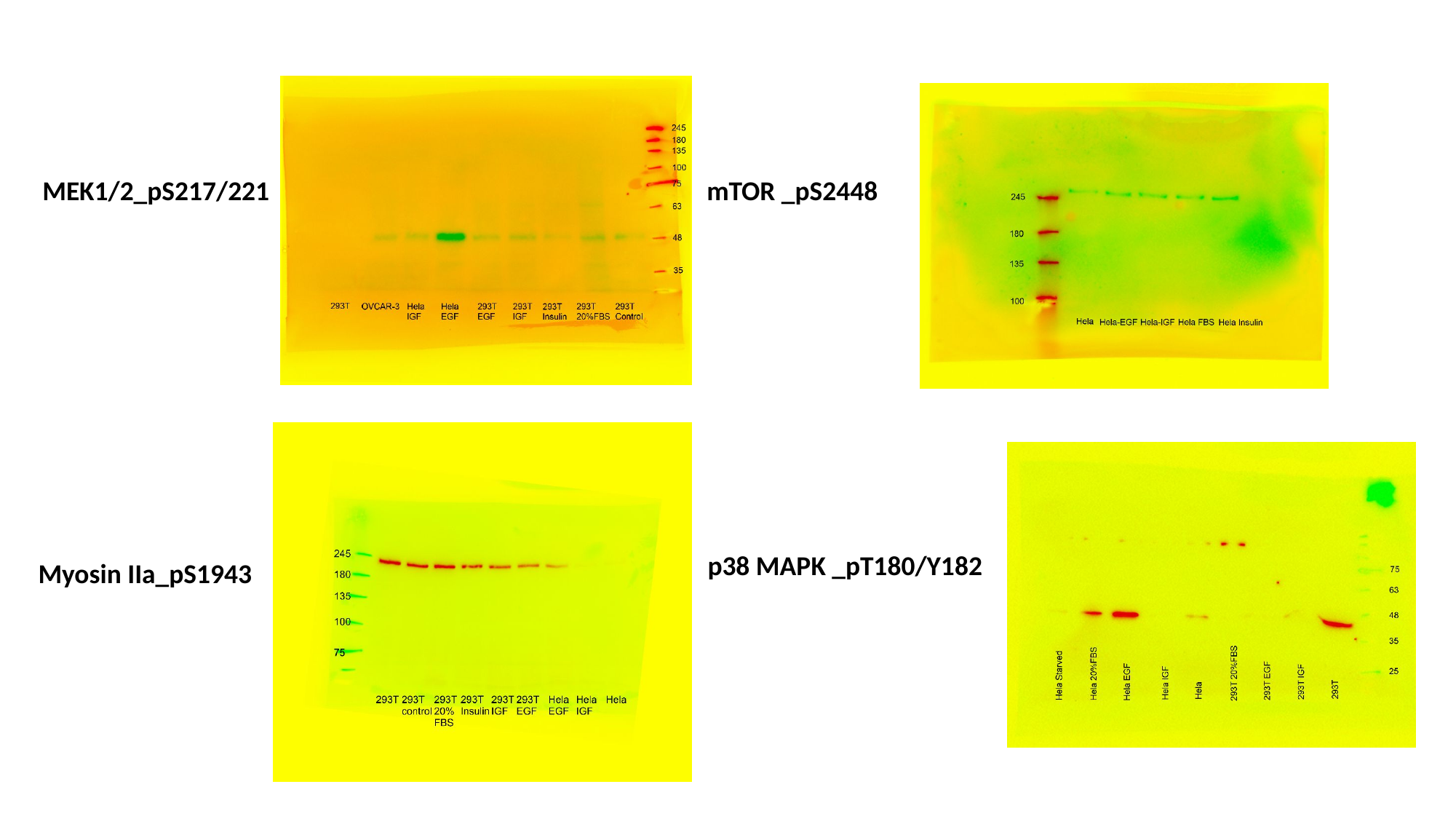

MEK1/2_pS217/221
mTOR _pS2448
p38 MAPK _pT180/Y182
Myosin IIa_pS1943

## Slide 7
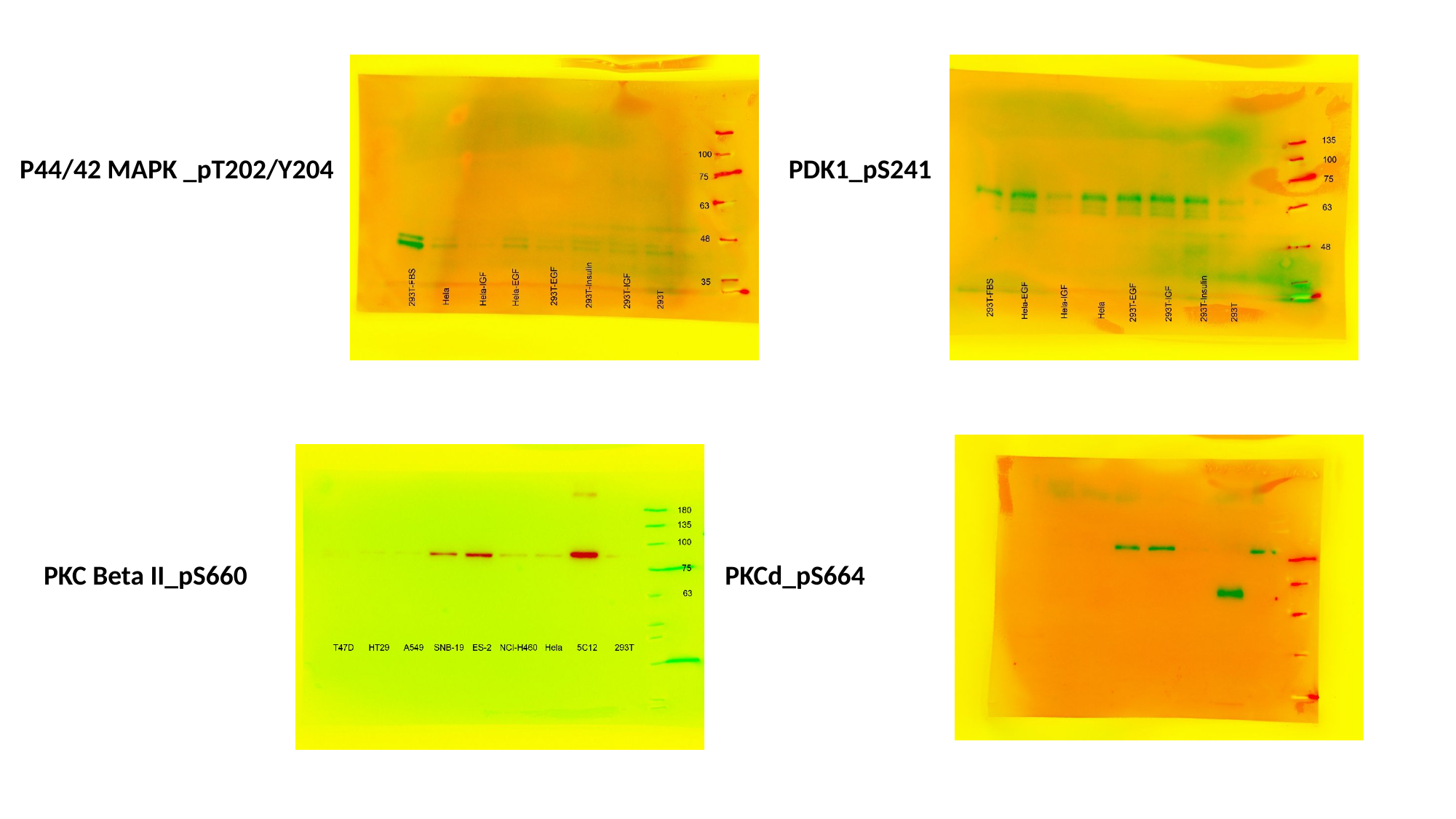

P44/42 MAPK _pT202/Y204
PDK1_pS241
PKC Beta II_pS660
PKCd_pS664

## Slide 8
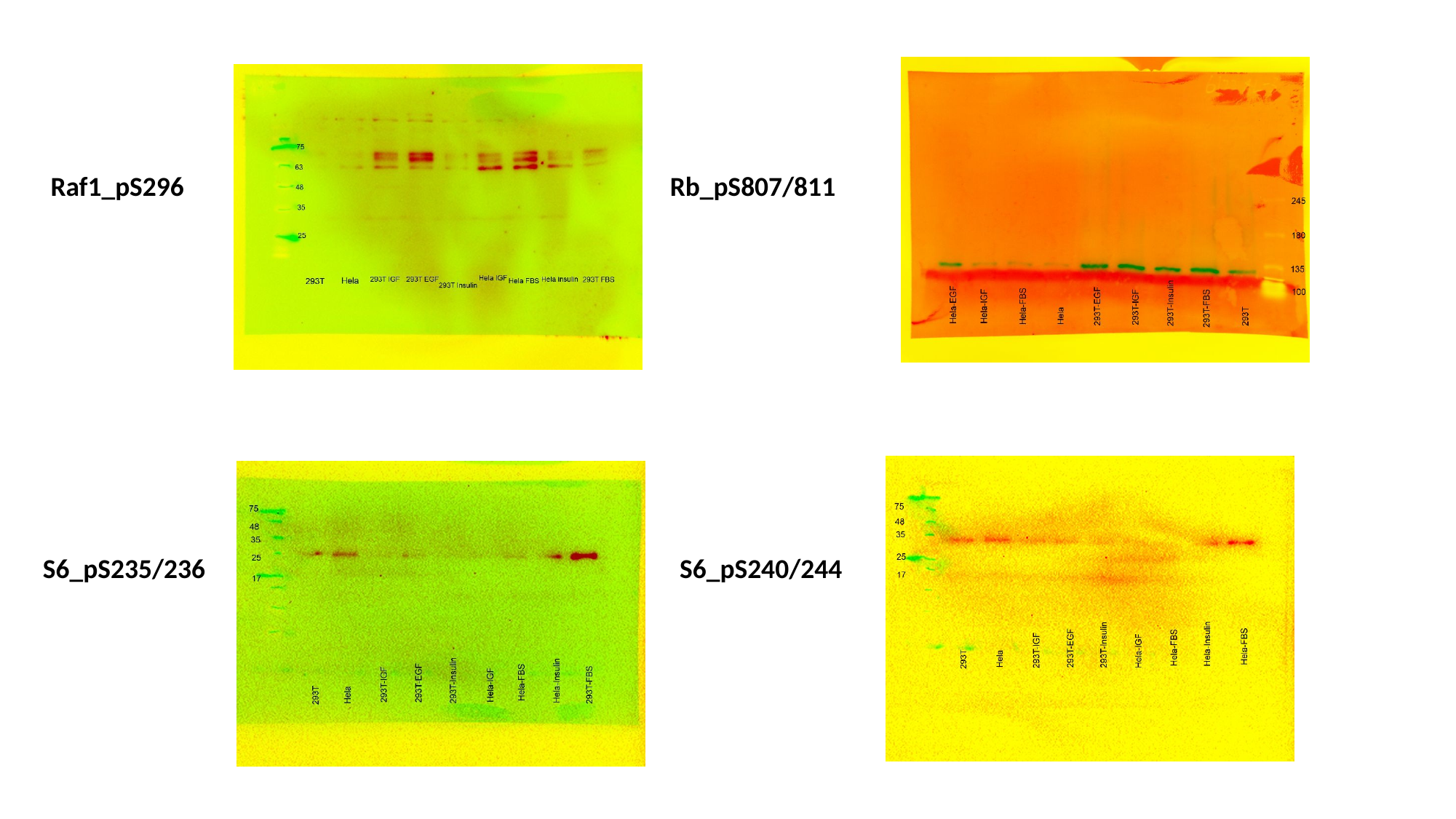

Raf1_pS296
Rb_pS807/811
S6_pS235/236
S6_pS240/244

## Slide 9
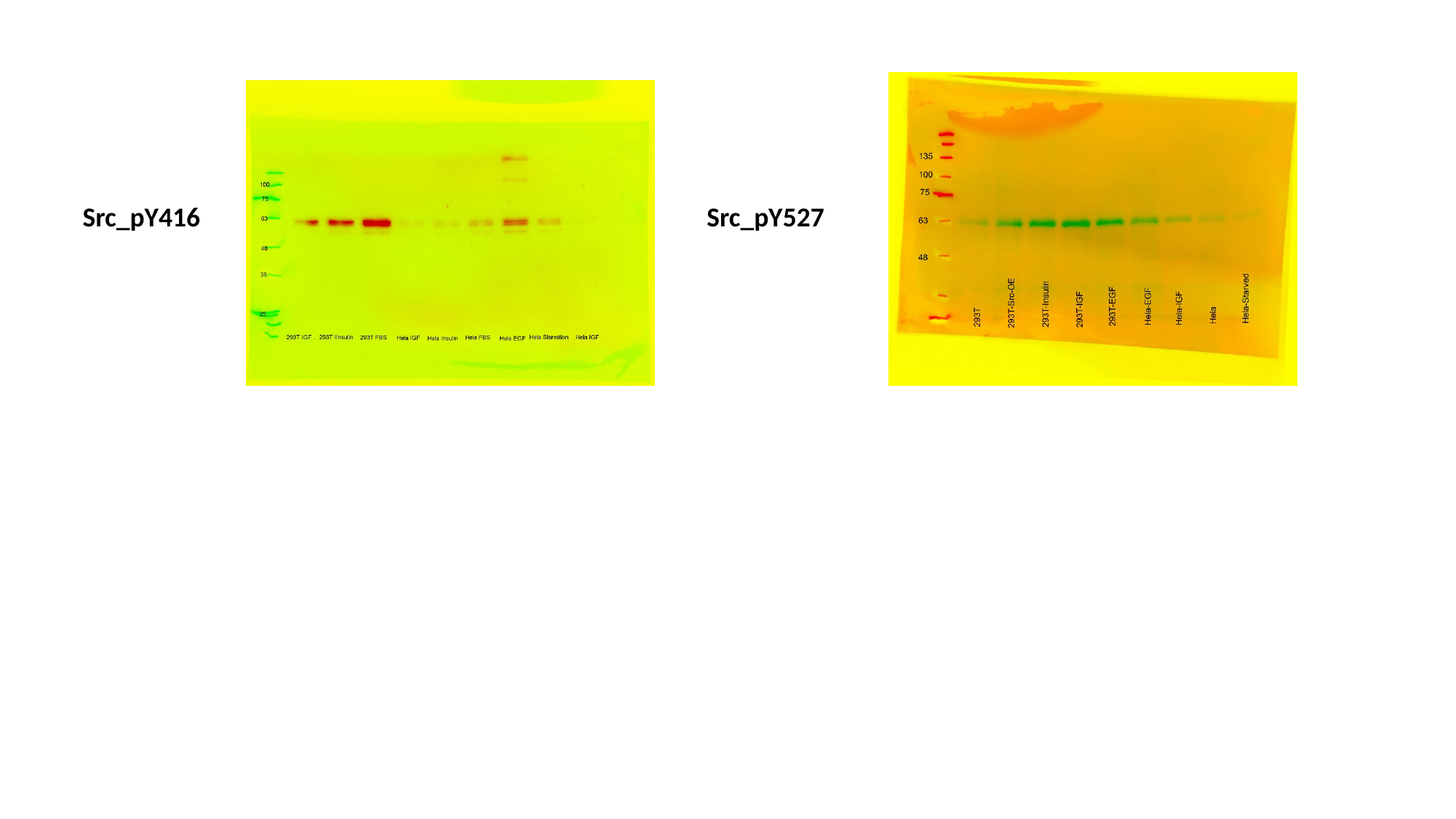

Src_pY416
Src_pY527

## Slide 10
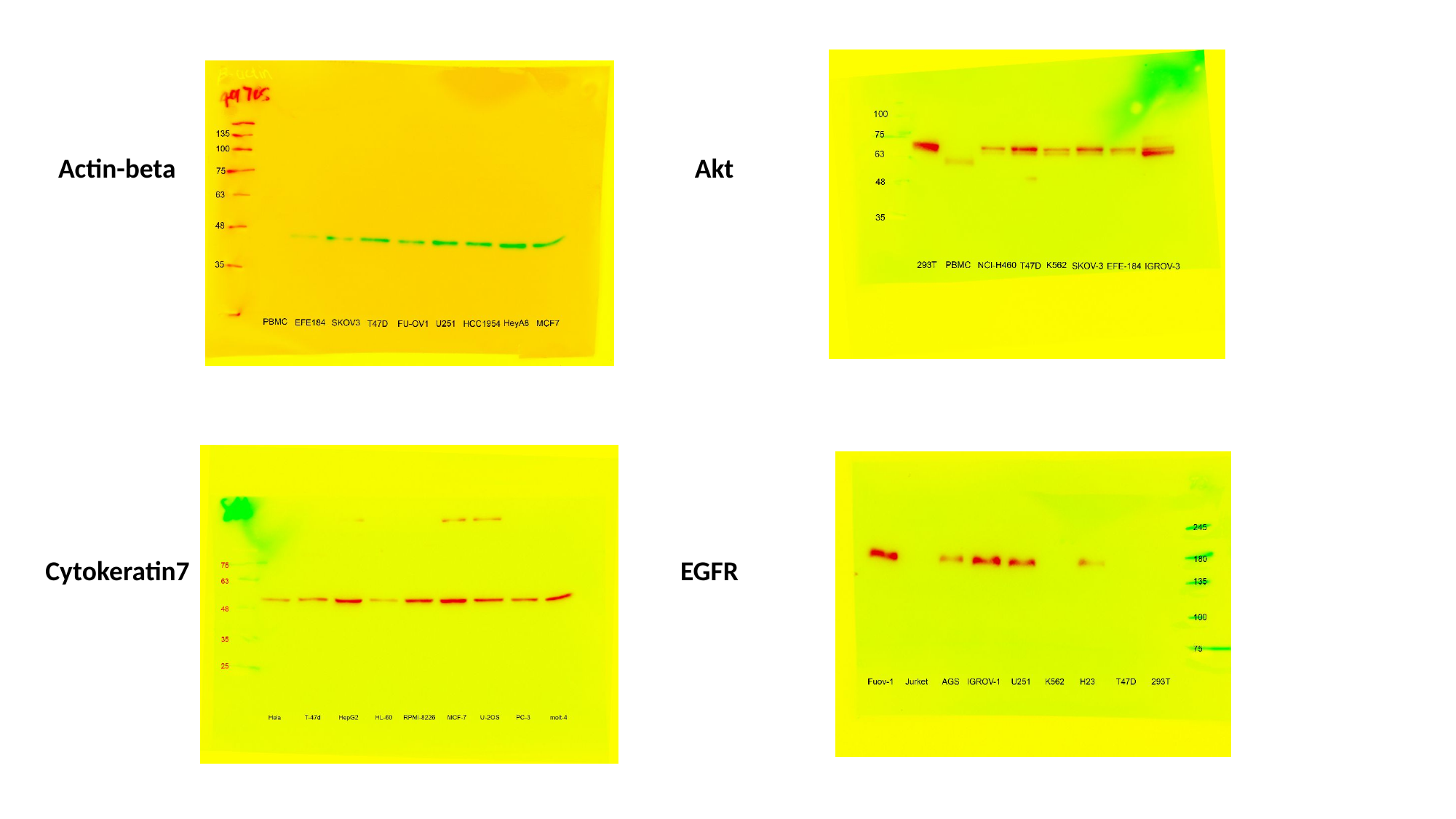

Akt
Actin-beta
Cytokeratin7
EGFR

## Slide 11
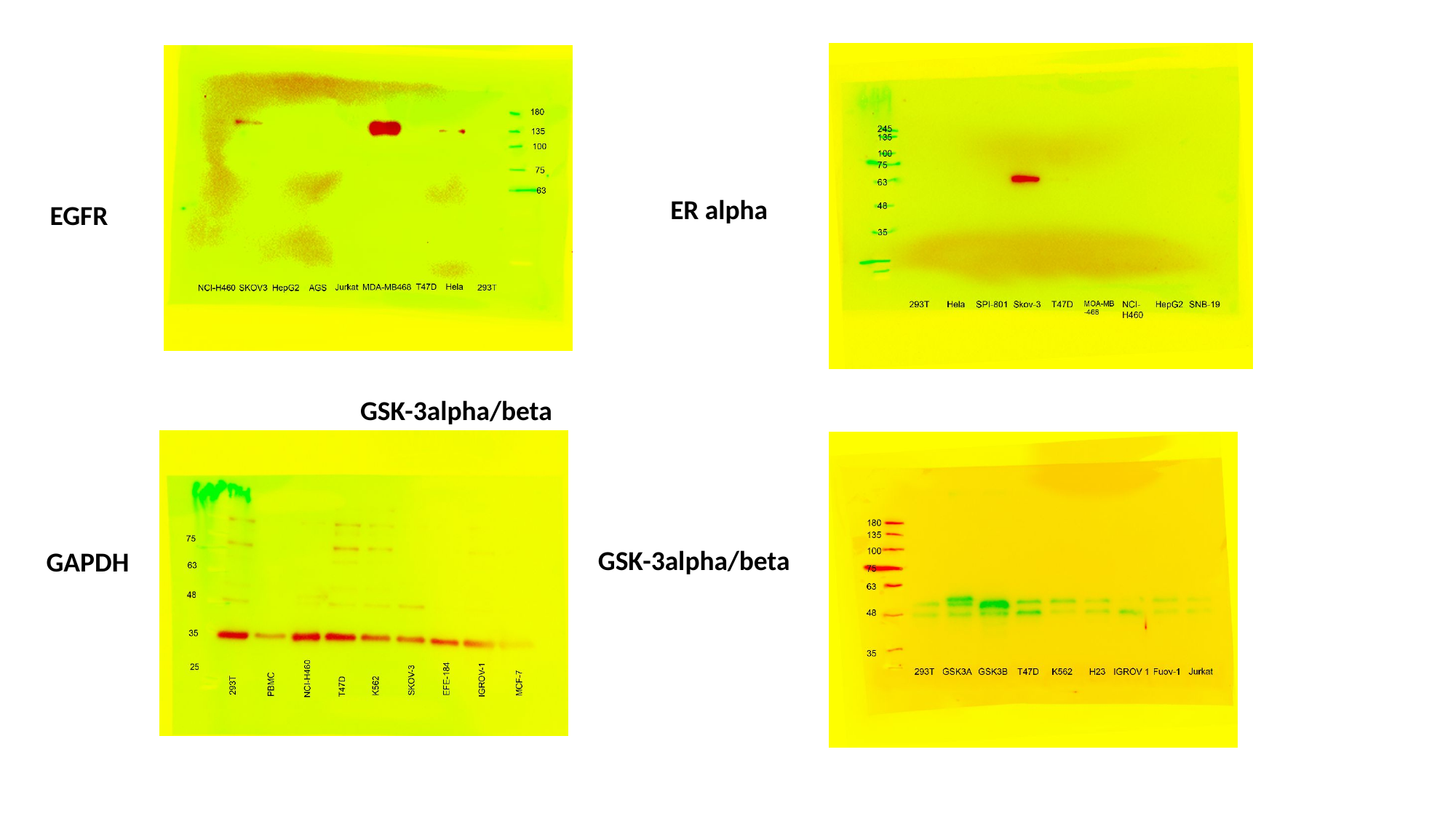

ER alpha
EGFR
GSK-3alpha/beta
GSK-3alpha/beta
GAPDH

## Slide 12
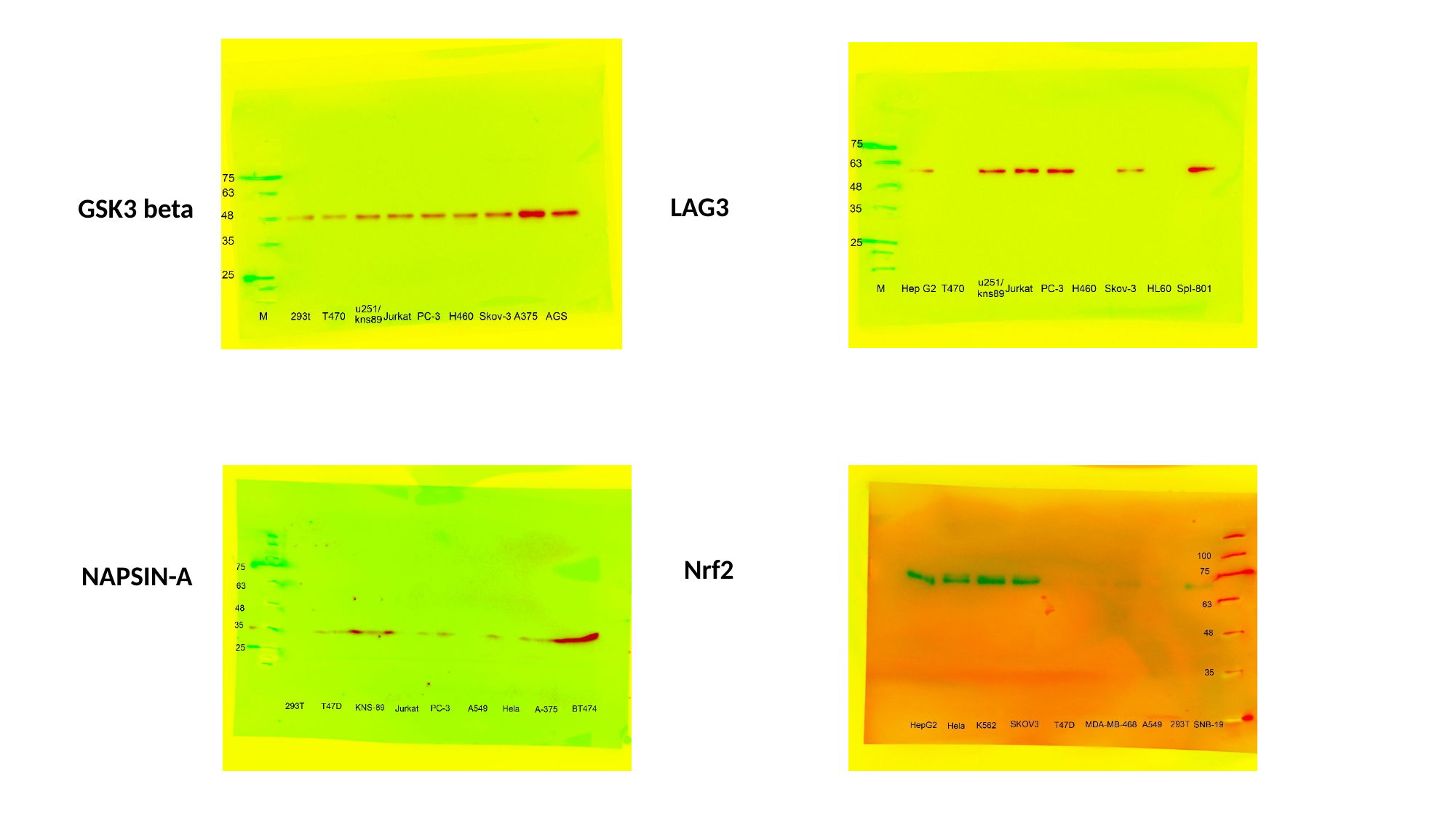

LAG3
GSK3 beta
Nrf2
NAPSIN-A

## Slide 13
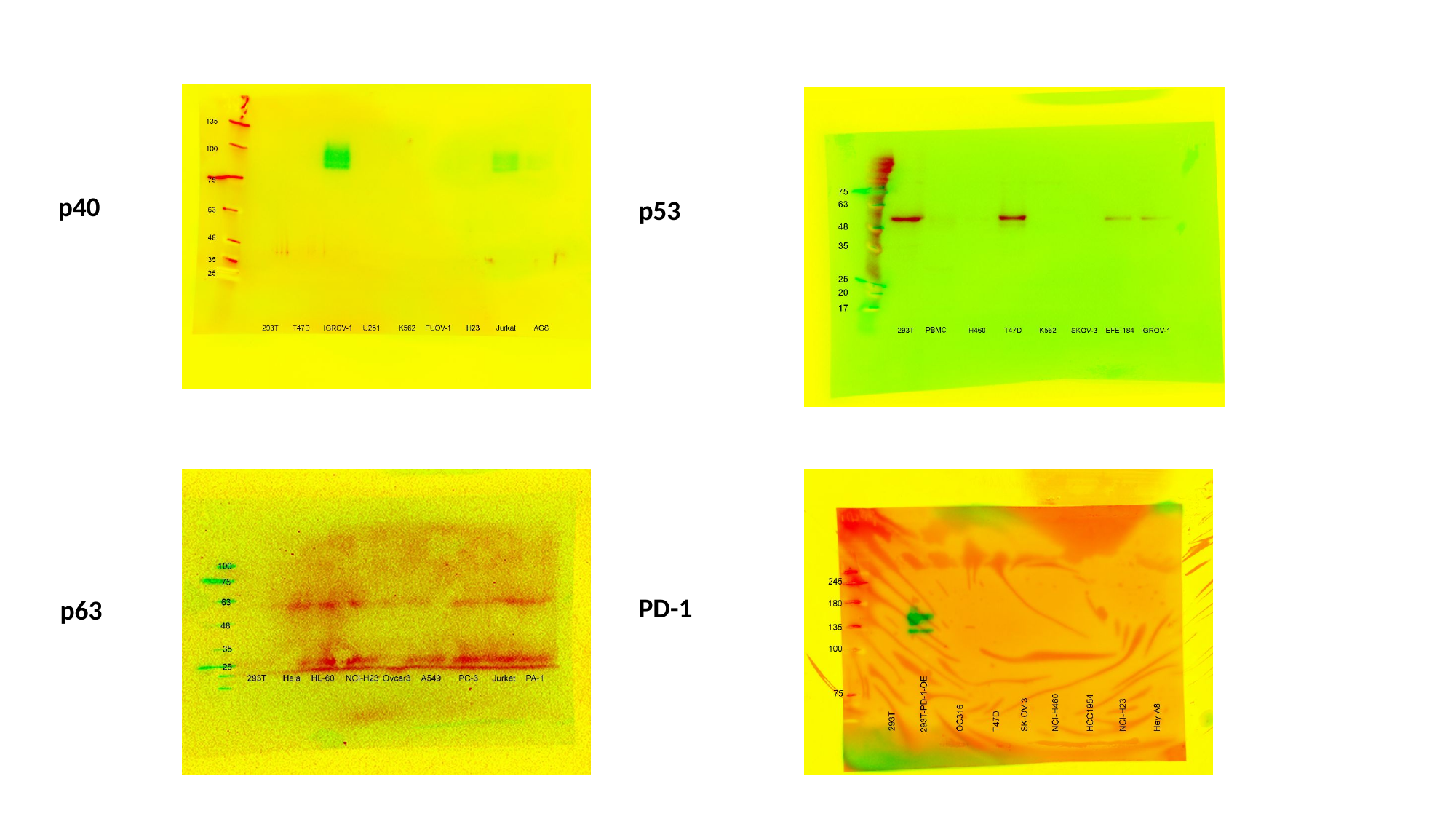

p40
p53
PD-1
p63

## Slide 14
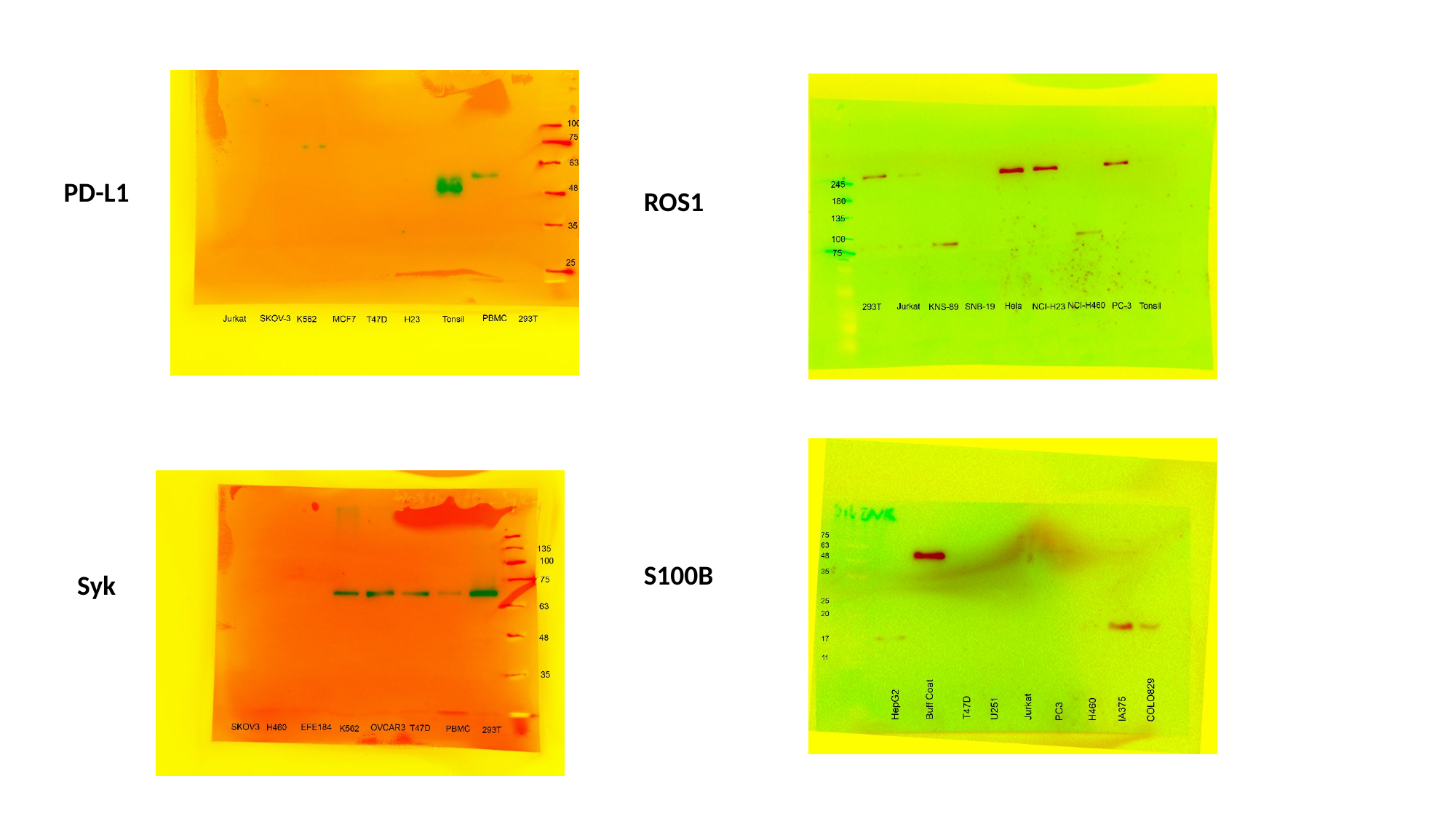

PD-L1
ROS1
S100B
Syk

## Slide 15
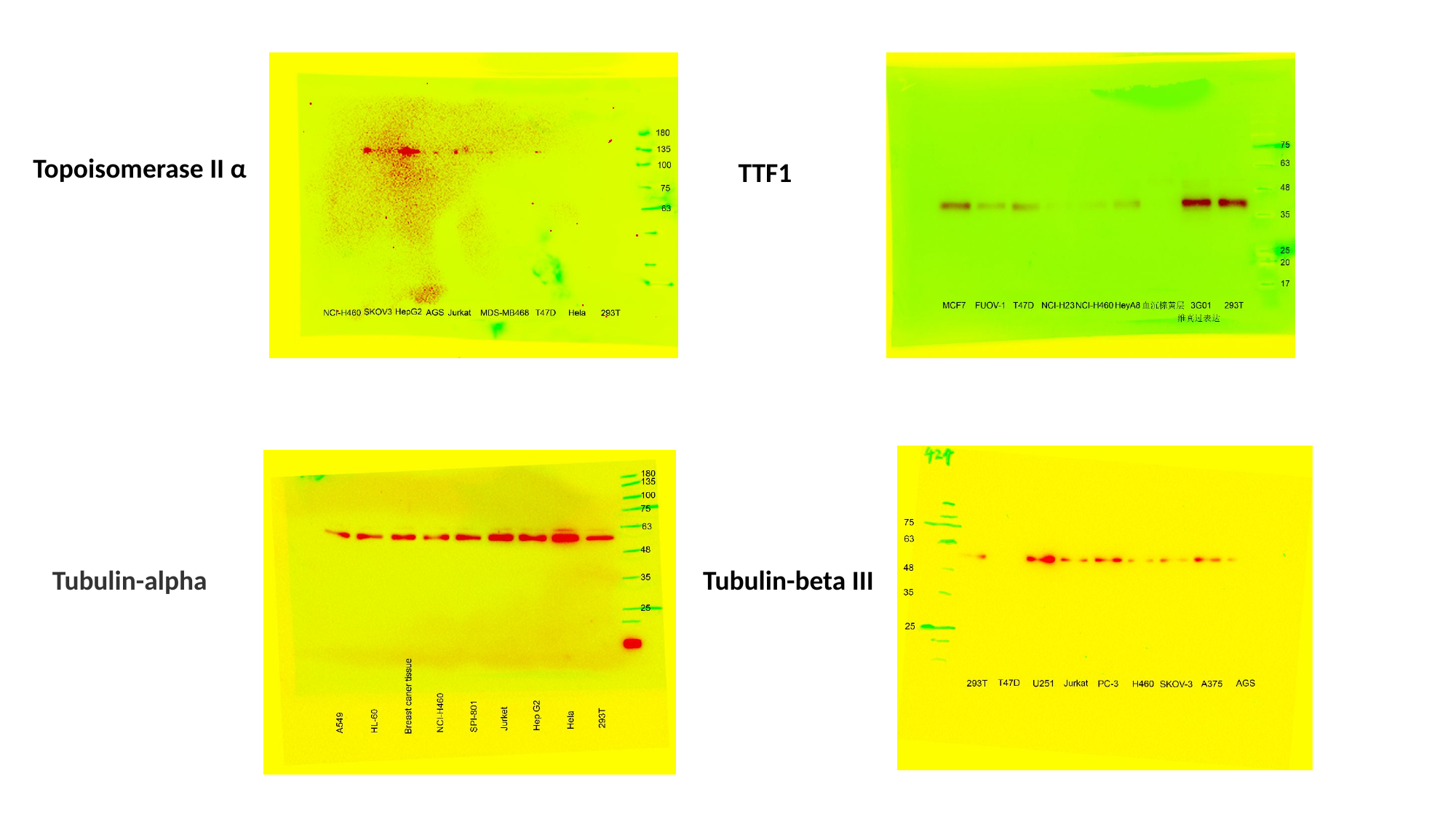

Topoisomerase II α
TTF1
Tubulin-alpha
Tubulin-beta III

## Slide 16
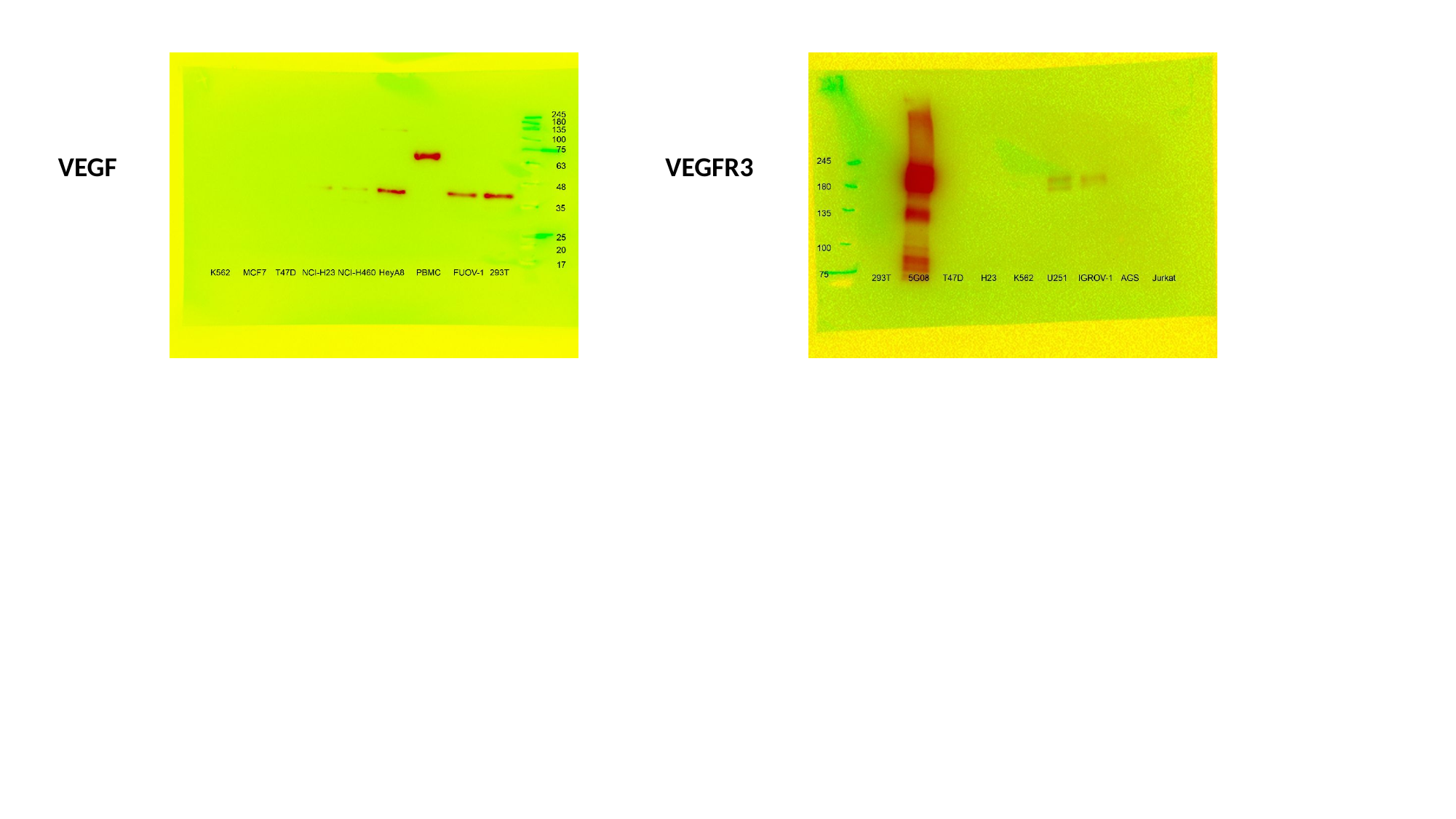

VEGF
VEGFR3

## Slide 17
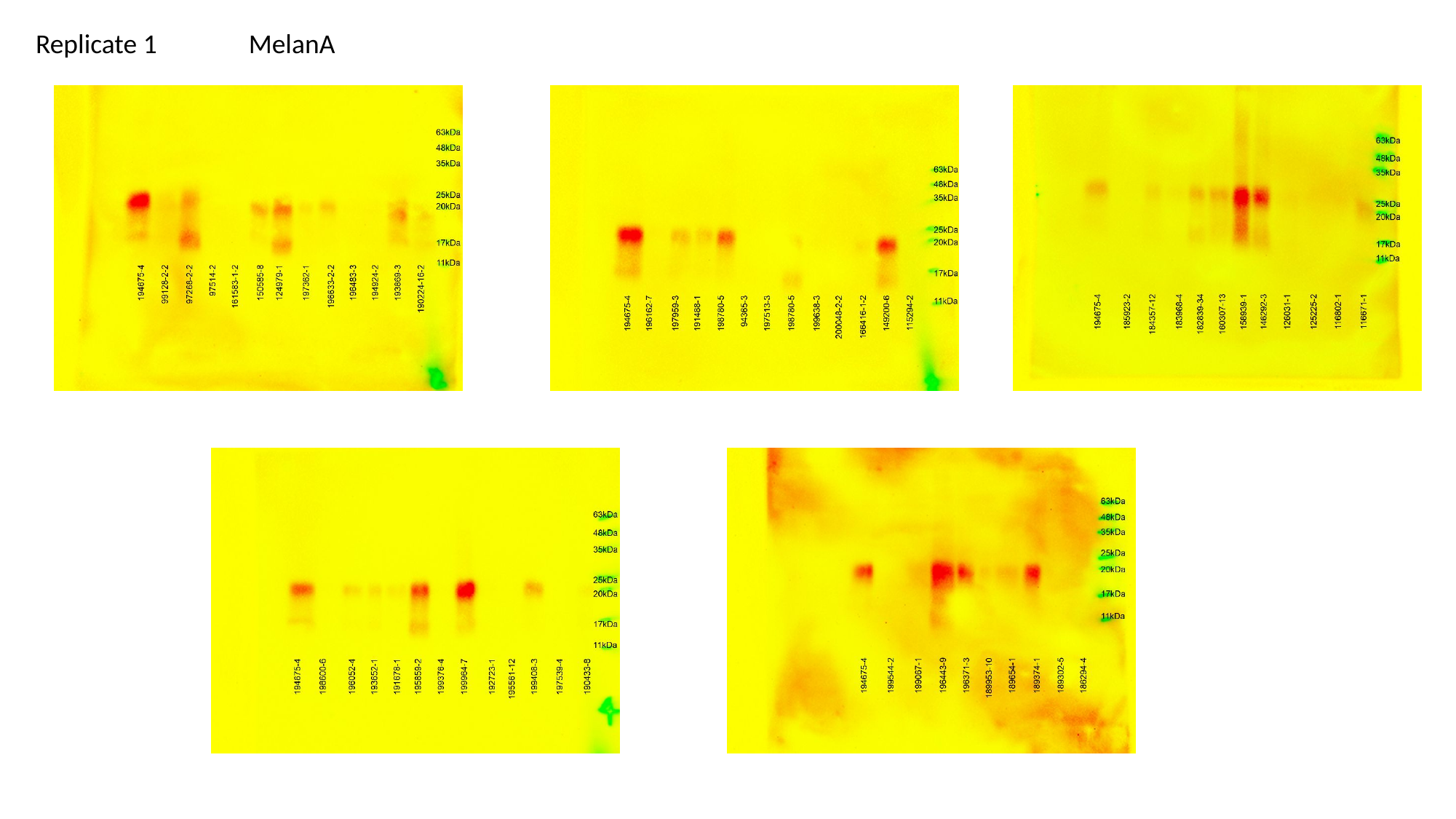

Replicate 1
MelanA

## Slide 18
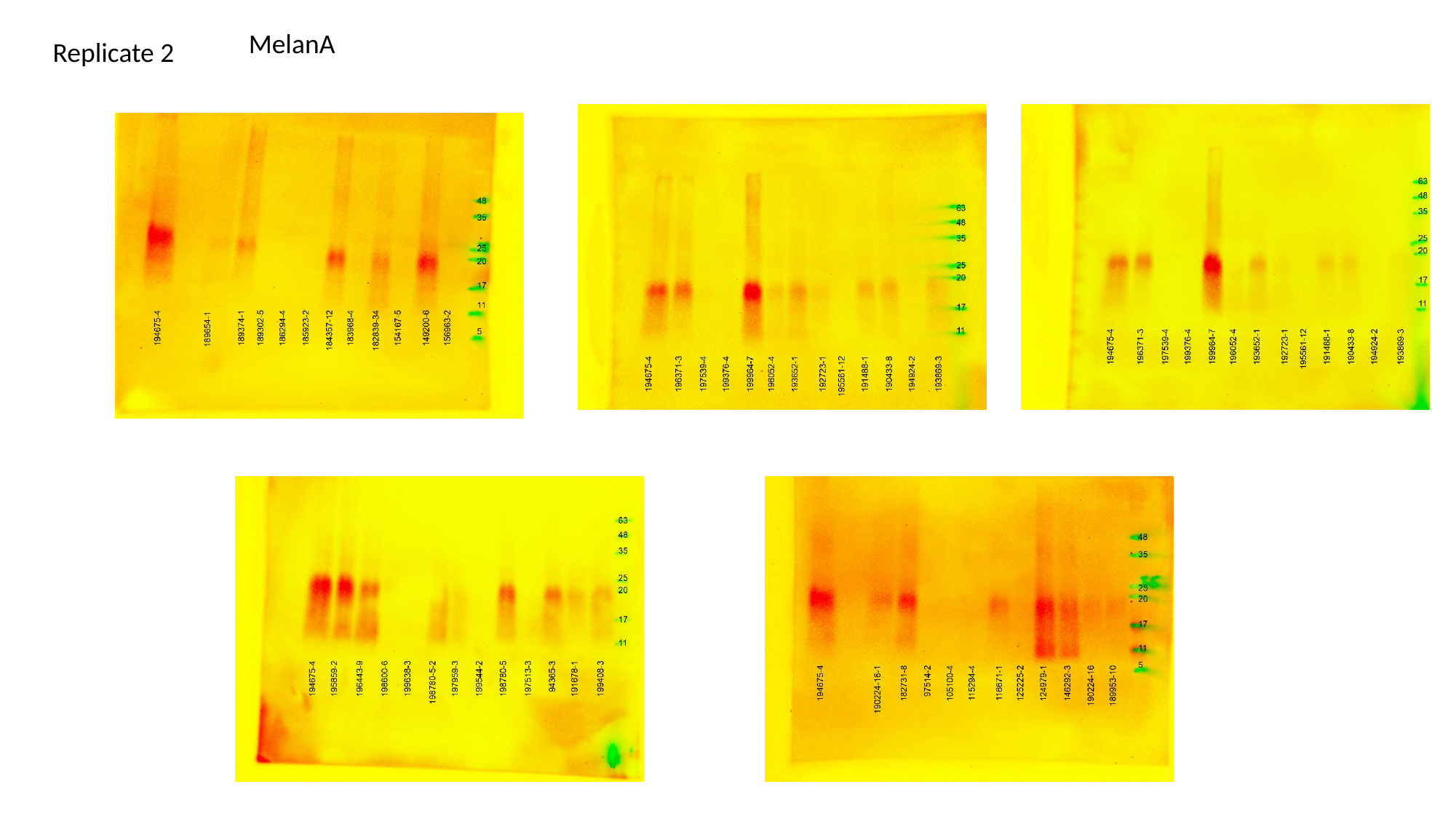

MelanA
Replicate 2

## Slide 19
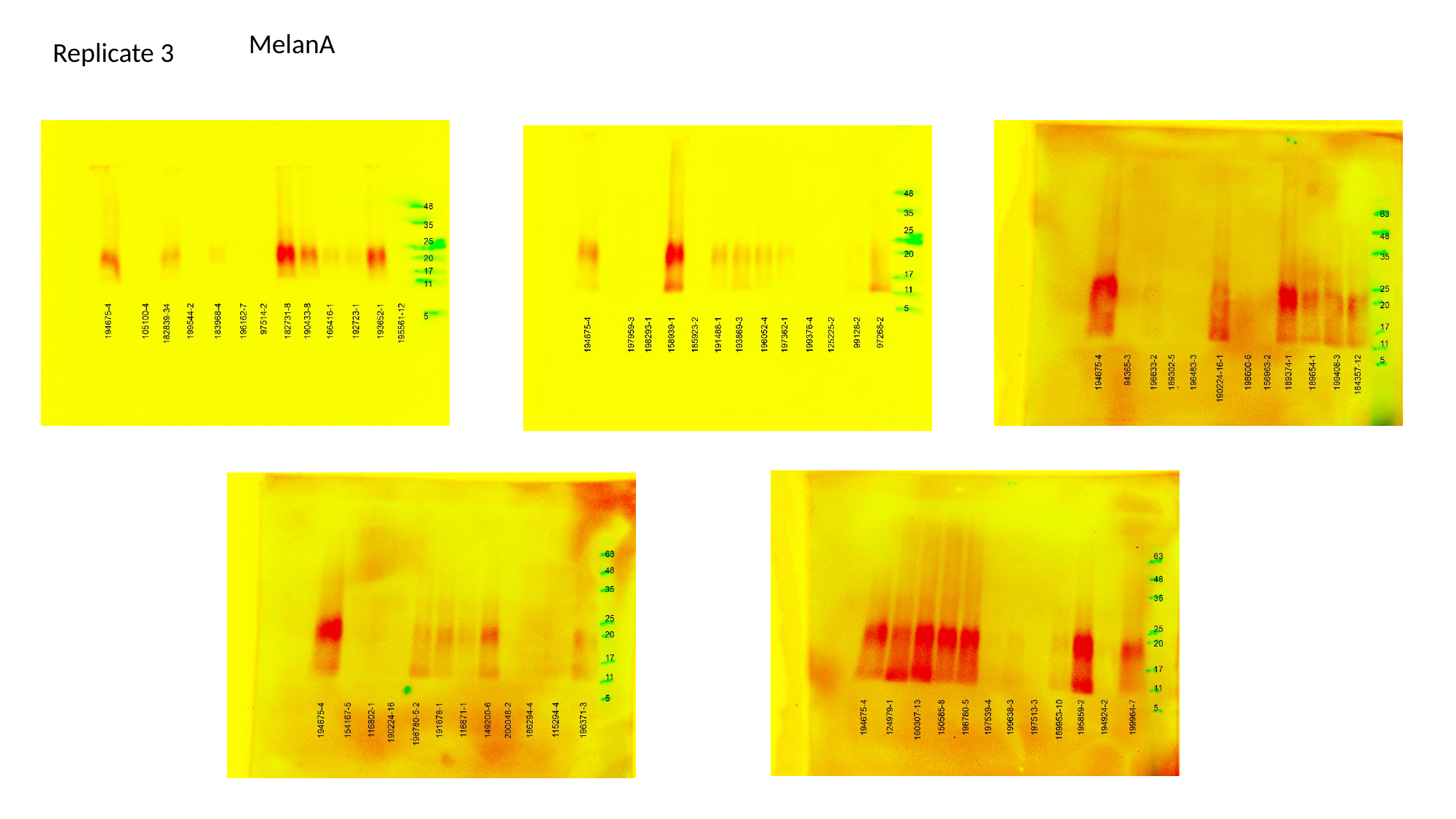

MelanA
Replicate 3
