# Supplementary material for: A reverse phase protein array based phospho-antibody characterization approach and its applicability for clinical derived tissue specimens
Source: Sci Rep. 2022 Dec 26;12:22373. doi: 10.1038/s41598-022-26715-9 (PMC9792559; doi:10.1038/s41598-022-26715-9)
Supplement: Supplementary file 8 — Supplementary Tables. [file 41598_2022_26715_MOESM8_ESM.pptx]

## Slide 1
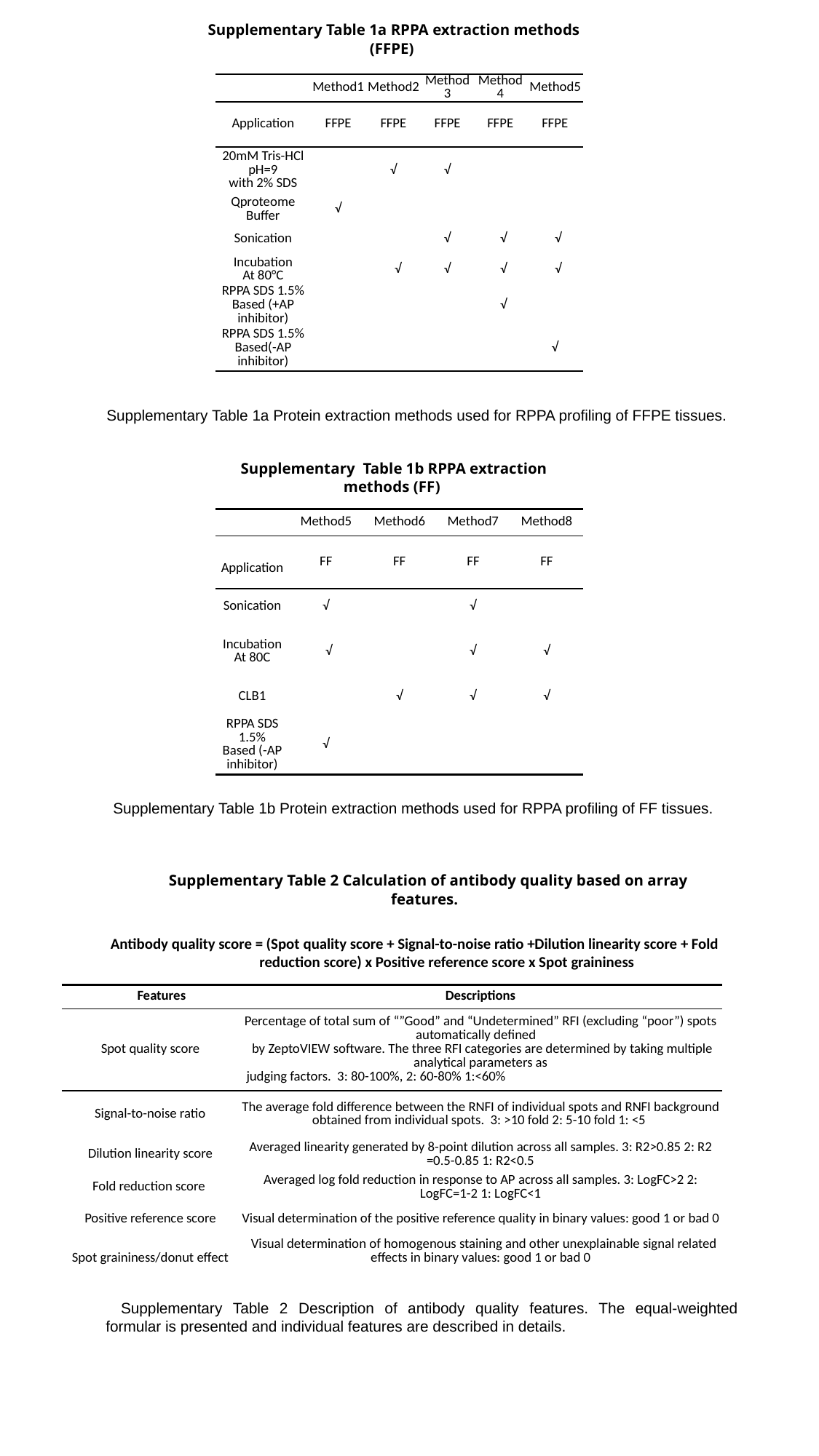

Supplementary Table 1a RPPA extraction methods (FFPE)
| | Method1 | Method2 | Method3 | Method4 | Method5 |
| --- | --- | --- | --- | --- | --- |
| Application | FFPE | FFPE | FFPE | FFPE | FFPE |
| 20mM Tris-HCl pH=9 with 2% SDS | | √ | √ | | |
| Qproteome Buffer | √ | | | | |
| Sonication | | | √ | √ | √ |
| Incubation At 80°C | | √ | √ | √ | √ |
| RPPA SDS 1.5% Based (+AP inhibitor) | | | | √ | |
| RPPA SDS 1.5% Based(-AP inhibitor) | | | | | √ |
Supplementary Table 1a Protein extraction methods used for RPPA profiling of FFPE tissues.
 Supplementary Table 1b RPPA extraction methods (FF)
| | Method5 | Method6 | Method7 | Method8 |
| --- | --- | --- | --- | --- |
| Application | FF | FF | FF | FF |
| Sonication | √ | | √ | |
| Incubation At 80C | √ | | √ | √ |
| CLB1 | | √ | √ | √ |
| RPPA SDS 1.5% Based (-AP inhibitor) | √ | | | |
Supplementary Table 1b Protein extraction methods used for RPPA profiling of FF tissues.
 Supplementary Table 2 Calculation of antibody quality based on array features.
Antibody quality score = (Spot quality score + Signal-to-noise ratio +Dilution linearity score + Fold
 reduction score) x Positive reference score x Spot graininess
| Features | Descriptions |
| --- | --- |
| Spot quality score | Percentage of total sum of “”Good” and “Undetermined” RFI (excluding “poor”) spots automatically defined by ZeptoVIEW software. The three RFI categories are determined by taking multiple analytical parameters as judging factors. 3: 80-100%, 2: 60-80% 1:<60% |
| Signal-to-noise ratio | The average fold difference between the RNFI of individual spots and RNFI background obtained from individual spots. 3: >10 fold 2: 5-10 fold 1: <5 |
| Dilution linearity score | Averaged linearity generated by 8-point dilution across all samples. 3: R2>0.85 2: R2 =0.5-0.85 1: R2<0.5 |
| Fold reduction score | Averaged log fold reduction in response to AP across all samples. 3: LogFC>2 2: LogFC=1-2 1: LogFC<1 |
| Positive reference score | Visual determination of the positive reference quality in binary values: good 1 or bad 0 |
| Spot graininess/donut effect | Visual determination of homogenous staining and other unexplainable signal related effects in binary values: good 1 or bad 0 |
Supplementary Table 2 Description of antibody quality features. The equal-weighted formular is presented and individual features are described in details.
